# Supplementary material for: Health economic evaluation of a serum-based blood test for brain tumour diagnosis: exploration of two clinical scenarios
Source: BMJ Open. 2018 May 24;8(5):e017593. doi: 10.1136/bmjopen-2017-017593 (PMC5988134; doi:10.1136/bmjopen-2017-017593)
Supplement: Supplementary file 1 [file bmjopen-2017-017593supp001.pdf]

SUPPLEMENTARY MATERIAL

Appendix 1 – Clinical expert group

Clinical expert group participants: Mr Michael D Jenkinson (Walton Centre NHS Trust & University of Liverpool), Dr Paul Brennan (University of Edinburgh), Prof. Anthony Chalmers (University of Glasgow & Beatson Centre), Prof. Willie Hamilton (University of Exeter Medical School), Mr Kevin O'Neill (Imperial College Healthcare NHS Trust), Dr Allan Janes (Beatson West of Scotland Cancer Centre), Dr Babar Vaqas (Imperial College Healthcare NHS Trust), Dr David Kernick (St Thomas Health Centre, Exeter), Dr James Livermore (University of Oxford), Dr Ruth Board (Lancashire Teaching Hospitals NHS Trust), Prof. Timothy Dawson (Lancashire Teaching Hospitals NHS Trust), Miss Elvira Lekka (Lancashire Teaching Hospitals NHS Trust), Mr John Goodden (Leeds Teaching Hospital NHS Trust) and Dr. Samantha Mills (Walton Centre NHS Trust)

Appendix 2 – Decision tree model

The decision node probabilities that populate the decision tree model are reported in Table S1. The model is simplified by assuming that the reference test (MRI/CT) has perfect accuracy (100% sensitivity and 100% specificity). This is desirable if we wish to evaluate the test against an MRI/CT only diagnostic pathway in which these imaging studies are considered as the 'gold standard'. It is also difficult to obtain valid estimates of MRI/CT sensitivity and specificity for this indication as these are not reported in a comparable manner in the literature. This simplification will not substantially alter the economic results compared to using more realistic values for the scenarios under consideration. This is because it is assumed confirmation of the diagnosis always requires imaging and therefore outcomes for patients that would hypothetically be classified correctly using this test but incorrectly by MRI/CT will remain the same whether or not the test is used. The effect of less than perfect accuracy of the reference test is therefore the same as a (small) decrease in prevalence as a proportion of patients with the disease cannot benefit from the additional test due to incorrect subsequent testing. Outcomes are calculated by 'rolling-back' the tree for both cancer cases and non-cases and taking an average of the two groups weighted by the prevalence of brain tumours in the scenario population.

Table S1. Decision node probabilities for decision tree model describing the integration of a serum spectroscopy test in the current diagnostic pathway

**Cancer cases:**	**Probability to upper branch**	**Probability to lower branch**
Serum Spectroscopy Test (test sensitivity)	0·928	0·072
Standard MRI/CT (reference test sensitivity)	1	0
Fast-track MRI/CT (reference test sensitivity)	1	0
Referral decision	S1:0·5, S2: 1	S1:0·5, S2: 0
Non-cases:		
Serum Spectroscopy Test (test specificity)	0·085	0·915
Standard MRI/CT (reference test specificity)	0	1
Fast-track MRI/CT (reference test specificity)	0	1
Referral decision	S1:0·5, S2: 1	S1:0·5, S2: 0

S1 & S2: Scenarios 1 &2

Appendix 3 – Assumptions and details of cost effective analysis model

Effect of testing on time-to-diagnosis and time-to-treatment
To estimate the expected time-to-diagnosis under a fast-track pathway it is assumed that this would match the current median time-to-diagnosis for patients presenting with brain tumour in emergency care as observed in Aggarwal et al [5]. This study reported a cohort of high grade glioma patients from a single hospital in London, UK. No other suitable estimates of time-to-treatment or time-to-diagnosis were identified in the literature. This is the most common primary brain tumour in the indications considered in this evaluation. Median time-to-diagnosis for the standard pathway is also taken from the same source.

Effect of testing on use of imaging studies
The effect of spectroscopic testing on imaging study decisions of clinicians and patients is uncertain. Based on clinical expert input it was assumed, in the base case, that in secondary care all patients would continue to imaging, while in primary care 50% would continue to imaging following a negative spectroscopy result.

Effect of early diagnosis on patient outcomes
A systematic review of the literature failed to identify any studies that directly estimated the effect of earlier (or later) diagnosis on outcomes for primary brain tumours. Instead, based clinical expert guidance, estimates of the effects of time-to-treatment were sourced from the literature. The estimated effects are based on fitting natural history models to observational datasets of high grade glioma patients.[27,28] It is necessary to use a natural history model to produce survival estimates rather than using the survival data stratified by time-to-treatment directly, in order to adjust for potential confounding factors influencing both time-to-treatment and survival.

A one-to-one correspondence is assumed for the effects of an additional day added to the time-to-diagnosis and an additional day added to the time-to-treatment. Median survival by weeks between diagnosis and treatment are displayed in Table 2. The hazard ratio per additional day delay calculated in Do et al of 1·015 was applied to each day between diagnosis and treatment up to 28 days to calculate median survival time. Time between diagnosis and treatment beyond 28 days were fixed at the same median survival as 28 days.

Table S2. Relationship between time-to-diagnosis and survival

Time-to-diagnosis (weeks)	Median survival (weeks)
0	46
1	41·4
2	37·3
3	33·6
4	30·3

Source: Do et al 2000

Resource use and cost

It is assumed that there is no additional cost for converting a small proportion of standard referral to additional urgent referrals. This could be justified on the basis that patients receiving negative results allows more flexibility in the scheduling for these patients. This could create more opportunity to schedule patients with positive spectroscopy results sooner. As a larger proportion of patients are referred for immediate imaging it was assumed costs would increase because achieving this would require additional capacity. It was assumed that unit costs for MRI and CT grow exponentially with increasing proportion of immediate referrals such that costs double if 50% of patients are referred for immediate imaging.

Treatment costs are assumed to be the same for both fast-track and standard referrals that are diagnosed as cases. This may be a conservative assumption as treatment costs are likely to increase as disease progresses and therefore earlier treatment may, on average, be less costly than later treatment. However, it is also possible that

treatment costs could be increased if some cases are converted from palliative care to more aggressive treatment. The balance of these effects is unknown given the lack of appropriate data to estimate the effects. Secondary treatment and end-of-life costs are similarly assumed to be equal between fast track and standard referrals. This reflects the model assumption that all patients ultimately progress.

The time intervals considered (0-4 weeks) are too short for discounting to have an important impact on cost estimates therefore the effects of moving forward treatment on present value is not considered.

Appendix 4 - Total demand for serum spectroscopy tests in UK in two clinical scenarios

1. Primary care

An assumption that patients currently referred in direct-access imaging service would be selected for spectroscopic testing can provide a conservative estimate of the total number of patients likely to be tested. Three sources from the literature were considered:

1. A direct-access brain MRI service in Nottingham (UK) reported 130 patients per quarter (520 per year) among a population of 342,000.[32,33] The population of the UK is approximately 61 million therefore, assuming Nottingham is representative of the UK general population demand for brain MRI, there would be approximately 93,000 per year in the UK.
2. A direct access CT service for chronic headache in Glasgow (UK) reported 4404 patients referred over 8 years (551 per year) among a population of approximately 1 million.[31] Assuming this was representative of the UK generally then the demand for this type of service would be approximately 34,000 for the UK.
3. A direct access CT neuroimaging service in Lothian (UK) reported 389 exams per year for a population of approximately 442,000.[34] Assuming this was representative of the UK generally then the demand for this type of service would be approximately 54,000 for the UK.

The Glasgow estimate is particularly low and may result from limited uptake in the early part of the 8 year period for which data were reported. As it was not possible to determine if uptake increased over the period or was stable this estimate was not considered reliable. The Nottingham and Lothian estimates report potentially more stable and established services and are therefore more likely to reliably estimate demand. The likely demand for this novel test, if the indication is considered identical to neuroimaging, is therefore between 54,000 and 93,000. This is likely to be conservative as the test may well have a slightly broader indication than current neuroimaging. A reasonable point estimate may be approximately 75,000.

2. Secondary Care

An estimate of the total demand for serum spectroscopic tests in the UK can be made based on the reported incidence of brain tumour in the UK and the proportion of cases currently diagnosed through secondary (non-emergency) care. Annual incidence of malignant brain tumours in the UK is approximately 4,700.[35] Approximately one third of these are likely to present through secondary, non-emergency care. Using the positive predictive value for patients currently referred to neuroimaging in this setting of 3% suggests that for each case there will be 33 non-case patients receiving neuroimaging. In total, in the UK, this would imply 53,000 patients per year in secondary care may be suitable for spectroscopic testing.

Appendix 5 - Sensitivity Analysis

One-way sensitivity analysis (OWSA)
OWSA results for a range of test sensitivity are displayed in S1 and S2 as a series of points on a cost-effectiveness (CE) plane with incremental QALYs (intervention-control) on the x-axis and incremental costs (intervention - control) on the y-axis. Corresponding OWSA results for a range of test specificities in primary and secondary care are shown in Figure 3 and 4 in the main article text respectively. These are displayed with the ICER on the y-axis and the test specificity on the x-axis. Note that the estimated QALYs do not change with specificity in the model therefore changes in the ICER are due solely to changes in incremental costs.**Error! Reference source not found.** All OWSA analyses show results assuming the spectroscopy based test costs of £50 and £100.

Figure S1 – Results on CE plane over a range of test sensitivities (0·5-0·99) – Primary care

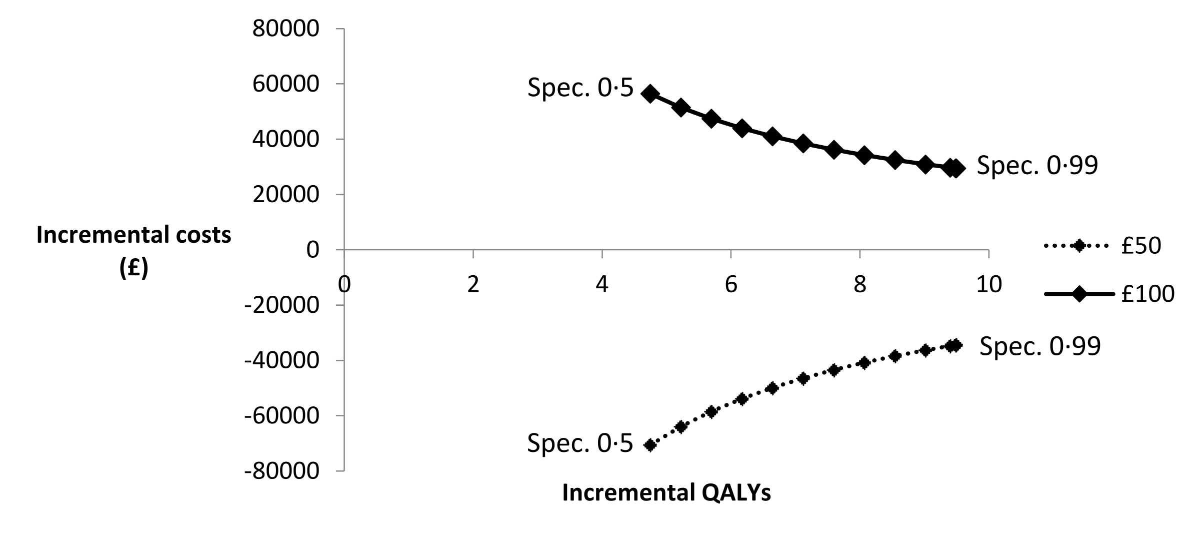

Figure S2 - Results on CE plane over a range of test sensitivities (0·5-0·99) – Secondary care

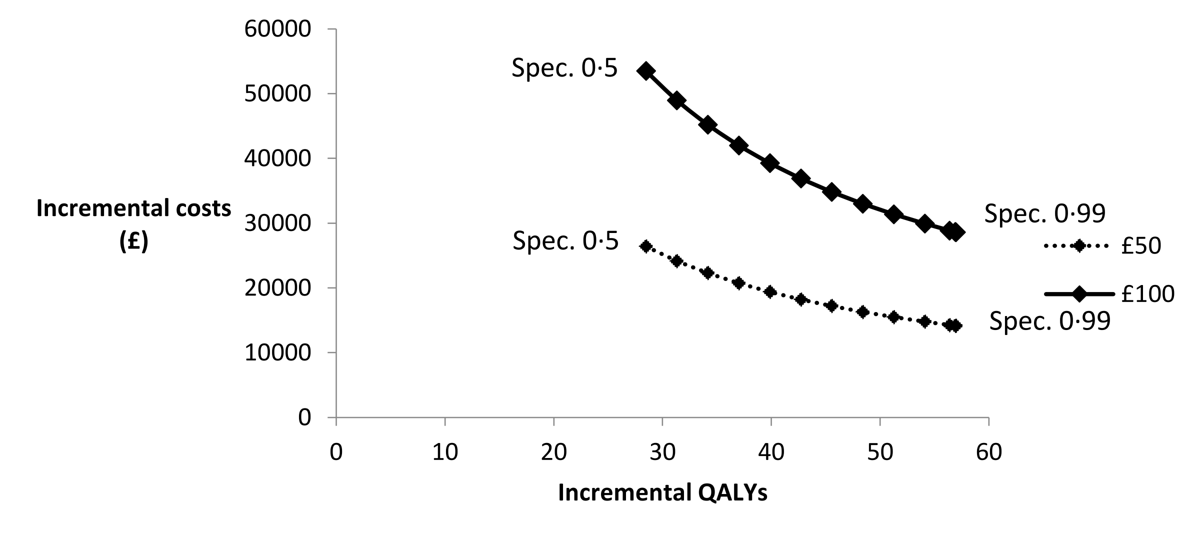

Scenario analysis

Table S3 – Scenario analysis: Mean survival estimates

	Scenario 1			Scenario 2		
Serum spectroscopy test cost (£)	ΔQALY	ΔCost	ICER	ΔQALY	ΔCost	ICER
50	12·83	-212808	-16582 (dominates)	77	540273	7016
100	12·83	287192	22377	77	1040273	13509

Table S4 - Additional costs scenario analysis

	Scenario 1			Scenario 2		
Serum spectroscopy test cost (£)	ΔQALY	ΔCost	ICER	ΔQALY	ΔCost	ICER
50	8·81	-379962	-43128 (dominates)	52·86	566247	10712
100	8·81	120038	13625	52·86	1066247	20171

Table S5 - Alternative prevalence estimates (1% prevalence) in primary care setting (Scenario 1)

	Scenario 1		
Serum spectroscopy test cost (£)	ΔQALY	ΔCost	ICER
50	17·62	-413220	-23452 (dominates)
100	17·62	86780	4925

Table S6– Alternative proportion of test negative cases referred to imaging (Scenario 1 only)

		Scenario 1		
Serum spectroscopy test cost (£)	% of test negative cases referred	ΔQALY	ΔCost	ICER
50	50 (base)	8·81	-422,116	-47,913
	75	17·62	-131,050	-7,438
	90	17·62	38,252	2,171
	100	17·62	151,120	8,577
100	50 (base)	8·81	77,884	8,840
	75	17·62	368,950	20,939
	90	17·62	538,252	30,548
	100	17·62	651,120	36,953

References

1 Burnet, N., Jefferies, S., Benson, R., Hunt, D. & Treasure, F. Years of life lost (YLL) from cancer is an important measure of population burden—and should be considered when allocating research funds. *Brit J Cancer* 2005; **92**: 241–5.

2 Hands, J. R., Clemens, G., Stables, R. *et al.* Brain tumour differentiation: rapid stratified serum diagnostics via attenuated total reflection Fourier-transform infrared spectroscopy. *J Neurooncol* 2016; **127**: 463–72.

3 Smith, B. R., Ashton, K. M., Brodbelt, A. *et al.* Combining random forest and 2D correlation analysis to identify serum spectral signatures for neuro-oncology. *Analyst* 2016; **141**: 3668–78.

4 Finding a better way? Improving the quality of life for people affected by brain tumours, Report Published by The Brain Tumour Charity, 2014.

5 Aggarwal, A., Herz, N., Campbell, P. *et al.* Diagnostic delay and survival in high-grade gliomas - evidence of the 'waiting time paradox'? *Br J Neurosurg* 2015; **29**: 520–3.

6 Zhou, Y., Abel, G. A., Hamilton, W. *et al.* Diagnosis of cancer as an emergency: a critical review of current evidence. *Nat Rev Clin Oncol* 2017; **14**: 45–56.

7 McPhail, S., Elliss-Brookes, L., Shelton, J. *et al.* Emergency presentation of cancer and short-term mortality. *Brit J Cancer* 2013; **109**: 2027–34.

8 Blanks, R., Moss, S., McGahan, C., Quinn, M. & Babb, P. Effect of NHS breast screening programme on mortality from breast cancer in England and Wales, 1990-8: comparison of observed with predicted mortality. *BMJ* 2000; **321**: 665–9.

9 UK Colorectal Cancer Screening Pilot Group. Results of the first round of a demonstration pilot of screening for colorectal cancer in the United Kingdom. *BMJ* 2004; **329**: 133.

10 Quinn, M., Babb, P., Jones, J. & Allen, E. Papers Effect of screening on incidence of and mortality from cancer of cervix in England: evaluation based on routinely collected statistics. *BMJ* 1999; **318**: 904.

11 Frankel, S., Smith, G. D., Donovan, J. & Neal, D. Screening for prostate cancer. *The Lancet* 2003; **361**: 1122–8.

12 Gadalla, S., Pfeiffer, R., Kristinsson, S. *et al.* Brain tumors in patients with myotonic dystrophy: a population-based study. *Eur J Neurol* 2016; **23**: 542–47.

13 Callaghan, B. C., Kerber, K. A., Pace, R. J., Skolarus, L. E. & Burke, J. F. Headaches and neuroimaging: high utilization and costs despite guidelines. *JAMA Intern Med* 2014; **174**: 819–21.

14 National Institute for Health and Care Excellence, 2015. Suspected cancer: recognition and referral. NICE Guideline (MG12).

15 Livermore, L. J., Ma, R., Bojanic, S. & Pereira, E. A. Yield and complications of frame-based and frameless stereotactic brain biopsy–the value of intra-operative histological analysis. *Brit J Neurosurg* 2014; **28**: 637–44.

16 Weber, M. A., Zoubaa, S., Schlieter, M. *et al.* Diagnostic performance of spectroscopic and perfusion MRI for distinction of brain tumors. *Cancer Imaging* 2006; **6**: S32–S41.

17 Baker, M. J., Trevisan, J., Bassan, P. *et al.* Using Fourier transform IR spectroscopy to analyze biological materials. *Nature Protoc* 2014; **9**: 1771–91.

18 Lovergne, L., Bouzy, P., Untereiner, V. *et al.* Biofluid infrared spectro-diagnostics: pre-analytical considerations for clinical applications. *Faraday Discuss* 2016; **187**: 521–37.

19 Baker, M. J., Hussain, S. R., Lovergne, L. *et al.* Developing and understanding biofluid vibrational spectroscopy: a critical review. *Chem Soc Rev* 2016; **45**: 1803–18.

20 Hochberg, F. H., Atai, N. A., Gonda, D. *et al.* Glioma diagnostics and biomarkers: an ongoing challenge in the field of medicine and science. *Expert Rev Mol Diagn* 2014; **14**: 439–52.

21 Hands, J. R., Abel, P., Ashton, K. *et al.* Investigating the rapid diagnosis of gliomas from serum samples using infrared spectroscopy and cytokine and angiogenesis factors. *Anal Bioanal Chem* 2013; **405**: 7347–55.

22 Wang, H. Z., Simonson, T. M., Greco, W. R. & Yuh, W. T. C. Brain MR Imaging in the Evaluation of Chronic Headache in Patients without Other Neurologic Symptoms. *Acad Radiol* 2001; **8**: 405–8.

23 Sempere, A. P., Porta-Etessam, J., Medrano, V. *et al.* Neuroimaging in the evaluation of patients with non-acute headache. *Cephalalgia* 2005; **25**: 30–5.

24 Clarke, C. E., Edwards, J., Nicholl, D. J. & Sivaguru, A. Imaging results in a consecutive series of 530 new patients in the Birmingham Headache Service. *J Neurol* 2010; **257**: 1274–9.

25 Kernick, D., Stapley, S., Goadsby, P. J. & Hamilton, W. What happens to new-onset headache presented to primary care? A case-cohort study using electronic primary care records. *Cephalalgia* 2008; **28**: 1188–95.

26 Hamilton, W. & Kernick, D. Clinical features of primary brain tumours: a case–control study using electronic primary care records. *Brit J Gen Pract* 2007; **57**: 695–9.

27 Do, V., Gebski, V. & Barton, M. The effect of wating for radiotherapy for grade II/IV gliomas. *Radiother Oncol* 2000; **57**: 131–6.

28 Burnet, N., Jefferies, S., Benson, R., Hunt, D. & Treasure, F. Years of life lost (YLL) from cancer is an important measure of population burden—and should be considered when allocating research funds. *Brit J Cancer* 2005; **92**: 241–45.

29 Garside, R., Pitt, M., Anderson, R. *et al.* The effectiveness and cost-effectiveness of carmustine implants and temozolomide for the treatment of newly diagnosed high-grade glioma: a systematic review and economic evaluation. *Health Technology Assessment* 2007; **11**.

30 National Institute for Health and Care Excellence, 2013. Guide to the methods of technology appraisal. NICE Guideline (PMG9).

31 Simpson, G. C., Forbes, K., Teasdale, E., Tyagi, A. & Santosh, C. Impact of GP direct-access computerised tomography for the investigation of chronic daily headache. *Br J Gen Pract* 2010; **60**: 897–901.

32 Taylor, T., Evangelou, N., Porter, H., Hamilton, W. & Kernick, D. Headache: two views on the right approach in general practice. *Br J Gen Pract* 2014; **64**: 475–6.

33 Taylor, T. R., Evangelou, N., Porter, H. & Lenthall, R. Primary care direct access MRI for the investigation of chronic headache. *Clin Radiol* 2012; **67**: 24–7.

34 White, P., Halliday-Pegg, J. & Collie, D. Open access neuroimaging for general practitioners - diagnostic yield and influence on patient management. *Brit J Gen Pract* 2002; **52**: 33–35.

35 UK Statistics Authority. Cancer Registration Statistics, England, 2014. Office of National Statistics
