# Supplementary material for: Health economic evaluation of a serum-based blood test for brain tumour diagnosis: exploration of two clinical scenarios
Source: BMJ Open. 2018 May 24;8(5):e017593. doi: 10.1136/bmjopen-2017-017593 (PMC5988134; doi:10.1136/bmjopen-2017-017593)
Supplement: Author's manuscript [file bmjopen-2017-017593.draft_revisions.pdf]

BMJ Open is committed to open peer review. As part of this commitment we make the peer review history of every article we publish publicly available.

When an article is published we post the peer reviewers' comments and the authors' responses online. We also post the versions of the paper that were used during peer review. These are the versions that the peer review comments apply to.

The versions of the paper that follow are the versions that were submitted during the peer review process. They are not the versions of record or the final published versions. They should not be cited or distributed as the published version of this manuscript.

BMJ Open is an open access journal and the full, final, typeset and author-corrected version of record of the manuscript is available on our site with no access controls, subscription charges or pay-per-view fees (http://bmjopen.bmj.com).

If you have any questions on BMJ Open's open peer review process please email info.bmjopen@bmj.com

BMJ Open

Health economic evaluation of a serum based blood test for brain tumour diagnosis: Exploration of two clinical scenarios.

Journal:	*BMJ Open*
Manuscript ID	bmjopen-2017-017593
Article Type:	Research
Date Submitted by the Author:	02-May-2017

Complete List of Authors:	Gray, Ewan; Health Improvement Scotland<br>Butler, Holly; Pure and Applied Chemistry; Technology & Innovation Building<br>Board, Ruth; Lancashire Teaching Hospitals NHS Foundation Trust, Rosemere Cancer Centre<br>Brennan, Paul; Western General Hospital, Department of Clinical Neurosciences<br>Chalmers, Anthony; University of Glasgow, Institute of Cancer Sciences; Beatson West of Scotland Cancer Centre<br>Dawson, Timothy; Lancashire Teaching Hospitals NHS Foundation Trust, Neurosurgery Department<br>Gooden, John; Leeds General Infirmary, Neurosurgery Department<br>Hamilton, Willie; University of Exeter Medical School, Primary Care Diagnostics<br>Hegarty, Mark; University of Strathclyde, Pure & Applied Chemistry<br>James, Allan; University of Glasgow Institute Of Molecular Cell And Systems Biology<br>Jenkinson, Michael; The Walton Centre NHS Foundation Trust<br>Kernick, David; St Thomas' Medical Group<br>Lekka, Elvira; Lancashire Teaching Hospitals NHS Foundation Trust, Neurosurgery Department<br>Livermore, Laurent; Oxford University Hospitals, NHS Foundation Trust, Department of Neurosurgery<br>Mills, Samantha; The Walton Centre NHS Foundation Trust<br>O'Neill , Kevin; Imperial College London<br>Palmer, David; University of Strathclyde, Pure & Applied Chemistry<br>Vaqas, Babar; Imperial College London, John Fulcher Neuro-Oncology Laboratory<br>Baker, Matthew; University of Strathclyde

**Primary Subject Heading**:	Health economics
Secondary Subject Heading:	Diagnostics, Health economics, Oncology
Keywords:	Biophysics < BASIC SCIENCES, HEALTH ECONOMICS, Neurology < INTERNAL MEDICINE, Neurological oncology < NEUROLOGY, Adult oncology < ONCOLOGY, Head & neck imaging < RADIOLOGY & IMAGING

For peer review only

Health economic evaluation of a serum based blood test for brain tumour diagnosis: Exploration of two clinical scenarios.

Ewan Gray[1†], Holly J. Butler[2,3†], Ruth Board[4], Paul M. Brennan[5], Anthony J Chalmers[6,7], Timothy Dawson[8], John Goodden[9], Willie Hamilton[10], Mark G. Hegarty[2,3], Allan James[11], Michael D. Jenkinson[12, 13], David Kernick[14], Elvira Lekka[8], Laurent J. Livermore[15], Samantha J. Mills[12], Kevin O'Neill[16], David S. Palmer[3,17], Babar Vaqas[16] Matthew J. Baker[2,3*]

[1] Health Improvement Scotland, NHS Scotland, Delta House, 50 West Nile Street, Glasgow, G1 2NP, UK; [2] WestCHEM, Department of Pure and Applied Chemistry, University of Strathclyde, Technology and Innovation Centre, 99 George Street, Glasgow, G1 1RD, UK; [3] ClinSpec Diagnostics Limited, Technology and Innovation Centre, 99 George St, G1 1RD, UK; [4] Rosemere Cancer Centre, Lancashire Teaching Hospitals NHS Trust, Royal Preston Hospital, Sharoe Green Lane, Preston, PR2 9HT, UK; [5] Department of Clinical Neurosciences, Western General Hospital, Edinburgh, EH4 2XU, UK; [6] Beatson West of Scotland Cancer Centre, 1053 Great Western Road, Glasgow, G12 0YN, UK; [7] Institute of Cancer Sciences, Wolfson Wohl Cancer Research Centre, University of Glasgow, Glasgow, G61 1QH; [8] Neurosurgery Department, Lancashire Teaching Hospitals NHS Trust, Royal Preston Hospital, Sharoe Green Lane, Preston, Lancashire, PR2 9HT, UK; [9] Neurosurgery Department, Leeds General Infirmary, Leeds, LS1 3EX, UK; [10] Primary Care Diagnostics, University of Exeter Medical School, College House, University of Exeter, Exeter, Devon, EX1 2LU, UK; [11] Institute of Molecular Cell and Systems Biology, Bower Building, Glasgow, G12 8QQ, UK; [12] The Walton Centre NHS Foundation Trust, Lower Lane, Fazakerley, Liverpool, L9 7LJ, UK; [13] Institute of Translational Medicine, Clinical Science Centre, University of Liverpool, Liverpool, L9 7LJ; [14] St Thomas' Medical Group, Cowick Street, Exeter, EX4 1HJ, UK; [15] Department of Neurosurgery, Oxford University Hospitals, NHS Foundation Trust, John Radcliffe Hospital, Oxford, OX3 9DU, UK; [16] John Fulcher Neuro-Oncology Laboratory, Imperial College, London, SW7 2AZ, UK; [17] WestCHEM, Department of Pure and Applied Chemistry, University of Strathclyde, Thomas Graham Building, 295 Cathedral Street, Glasgow, G1 1XL, UK

*Corresponding author: Dr Matthew J Baker, matthew.baker@strath.ac.uk, +44(0)141 548 4700, @ChemistryBaker

†These authors contributed equally to the paper

For peer review only

Copyright/Licence for publication

The Corresponding Author has the right to grant on behalf of all authors and does grant on behalf of all *authors,* a worldwide licence *to the Publishers and its licensees in perpetuity, in all forms, formats and media (whether known now or* created in the future), to i) publish, reproduce, distribute, display and store the Contribution, ii) translate the Contribution into other languages, create adaptations, reprints, include within collections and create summaries, extracts and/or, abstracts of the Contribution, iii) create any other derivative work(s) based on the Contribution, iv) to exploit all subsidiary rights in the Contribution, v) the inclusion of electronic links from the Contribution to third party material where-ever it may be located; and, vi) licence any third party to do any or all of the above

Authors' Contributions

EG conducted health economic assessment including data analysis, data interpretation, and writing. HJB contributed to data interpretation, writing and providing details regarding clinical spectroscopy. MJB is the principal investigator of this research, and initiated this interdisciplinary project and team. RB, PMB, AJC, JG, WH, AJ, MDJ, DK, EL, LJL, SJM, KO, and BV are members of the clinical focus group involved in spearheading translation; all of whom provided feedback on the manuscript. MGH, DSP, HJB and MJB are involved in the ClinSpec Diagnostics Limited project.

Competing Interest Declaration

MJB, MGH, HJB and DSP are all involved in ClinSpec Diagnostics Limited a prospective spin out company from the University of Strathclyde focusing on the translation of serum spectroscopic diagnostics (Company Number SC535447)

Role of the Funding Source

Scottish Enterprise is Scotland's main economic development agency and non-departmental public body of the Scottish Government. They work with partners in both the private and public sectors to exploit the best opportunities to deliver a significant, lasting effect on the Scottish economy. The High-Growth Spinout Programme helps researchers take their ideas and inventions from the lab to the global marketplace.

Acknowledgements

The authors acknowledge the support from Rosemere Cancer Foundation, Brain Tumour North West, EPSRC, and the Sydney Driscoll Neuroscience Foundation for funding.

For peer review only

Data Sharing Statement

No additional data available

For peer review only

Abstract

Objectives

To determine the health economic benefits of a serum-based spectroscopic triage tool for brain tumours, that could potentially reduce diagnostic delays in the current clinical pathway.

Design

A health economic assessment

Setting

Explored as a test in both primary and secondary care (neuroimaging) in the UK health service, as well as application to the US.

Participants

Calculations based upon an initial cohort of 10,000 patients. In primary care, it is estimated that the volume of tests would approach 75,000 per annum. The volume of tests in secondary care is estimated at 53,000 per annum.

Main Outcome Measures

The primary outcome measure was quality adjusted life years (QALYs), which were employed to derive incremental cost-effectiveness ratios (ICERs) in a cost-effectiveness analysis.

Results

Results indicate that using a blood-based spectroscopic test in both scenarios may be considered highly cost-effective in a health technology assessment (HTA) agency decision making process, as ICERs were well below standard threshold values of £20,000 to £30,000 per QALY. This test may be cost-effective in both scenarios with test sensitivities and specificities as low as 80%; however, the price of the test would need to be lower (less than approximately £40).

Conclusion

Use of this test as triage tool in primary care has the potential to be both more effective and cost saving for the health service. In secondary care, this test would also be deemed more effective than the current diagnostic pathway.

For peer review only

Strengths and Limitations of this Study

This health economic assessment presents a cost-effective blood test for early diagnosis of brain tumours, which may contribute towards improved prognosis for patients, as well as healthcare savings.

This test is highly sensitive and specific, with performances of 91.5 and 83.0% respectively.

Blood can be acquired using standard protocols, with the test requiring minimal sample preparation, and the rapid acquisition of results.

This project is a collaborative research project between leading neurosurgeons, neurooncologists, neuropathologists, neuroradiologists, and primary care experts, with award winning analytical scientists at the University of Strathclyde.

This study is based upon proof-of-concept studies, in advance of a pending clinical trial.

WHAT IS ALREADY KNOWN ON THIS TOPIC

Timely diagnosis of brain tumours, both primary and metastatic, is limited by the difficulty in identifying patients in primary care most likely to be affected, and patients in secondary care in urgent need of treatment.

Symptoms can be non-specific, necessitating expensive imaging technologies with low pick-up rates, and often invasive biopsies.

These factors contribute to delays in diagnosis, so are in part responsible for the poorer prognosis seen in brain tumours compared to other primary cancers.

WHAT THIS STUDY ADDS

A relatively non-invasive serum blood test using infrared spectroscopy, in conjunction with pattern recognition software, is show as a possible cost beneficial alternative to the current brain tumour diagnostic pathway.

In both primary care, this test is likely to be cost-effective and cost saving to the health service in the UK, and also USA; whilst also being cost-effective in secondary care.

For peer review only - http://bmjopen.bmj.com/site/about/guidelines.xhtml

Introduction

At an average of 20 years, patients with malignant brain tumours have the highest number of years of life lost, compared to all other primary cancers.[1] This, at least in part, may relate to diagnostic delays, reflecting the non-specific early symptoms, such as headache and dizziness, from which general practitioners (GPs) must identify patients at risk for further investigation. The perceived lack of a low-cost diagnostic and/or screening tools available within the health service also impacts on this delay. We have recently demonstrated that a spectroscopic test using blood-serum is able to effectively identify brain tumours in patients with sensitivities and specificities as high as 91·5% and 83% respectively.[2,3] This approach is based upon Fourier-transform infrared (FTIR) spectroscopy and can detect disease-specific signatures, which are extracted mathematically using pattern recognition and machine learning algorithms.

Current Diagnostic Pathway

Currently, patients with symptomatic brain tumour visit their GP on average five times before being referred to secondary care.[4] Partly as a result of this diagnostic delay up to 61% of brain tumour diagnoses occur in an emergency setting, often following a seizure.[5] Patients diagnosed by the emergency route have a poorer prognosis.[6,7] For some patients this may be because the disease is often of a more advanced stage at diagnosis, and the complication precipitating the emergency brings addition mortality *per se.*

Screening programmes for breast, prostate, cervical, and colorectal cancer have proved effective for diagnosing patients at an earlier stage, which can result in a better prognosis.[8-11] These screening programmes have had a significant impact in reducing the number of patients presenting as an emergency. There is no accessible and economically viable diagnostic tool for early detection of asymptomatic and symptomatic brain tumours.[12] The addition of a rapid and accurate blood test for patients with suspected primary brain tumours therefore has the potential to improve outcomes by allowing prioritisation of patients most at risk for further investigation. Under the current patient pathway it is not feasible to provide fast-track diagnostic imaging because the number of patients with non-specific headache symptoms is very large and the positive predictive value (PPV) on the basis of symptoms alone is less than 3% for all symptoms other than a new-onset seizure.[13,14]

Magnetic resonance imaging (MRI) and computed tomography (CT) are the current gold standard for identifying structural brain lesions including tumours. Treatment decisions made at the neuro-oncology multidisciplinary team (MDT) meeting are often based on the imaging alone, and following surgical resection or biopsy with histopathology and molecular analysis, definitive treatment can be planned.[15] Surgery to secure the tissue diagnosis has a small risk to the patient of neurological deterioration and death.[16] In additional to the patient risk, the diagnostic pathway also represents a significant cost burden to the health service, with a single MRI and CT scan in the UK costing around £164 and £85, respectively (National Schedule of Reference Costs [2014-15], Medicare Physician Fee Schedule 2016). A typical timeframe of the diagnostic pathway for brain tumours, specifically primary gliomas, is

For peer review only

illustrated in Figure 1, and effectively highlights the significant wait that a symptomatic patient may have before receiving brain imaging. Even from this stage, regardless of the time to GP referral or emergency presentation, full diagnosis may take a further 5 weeks.

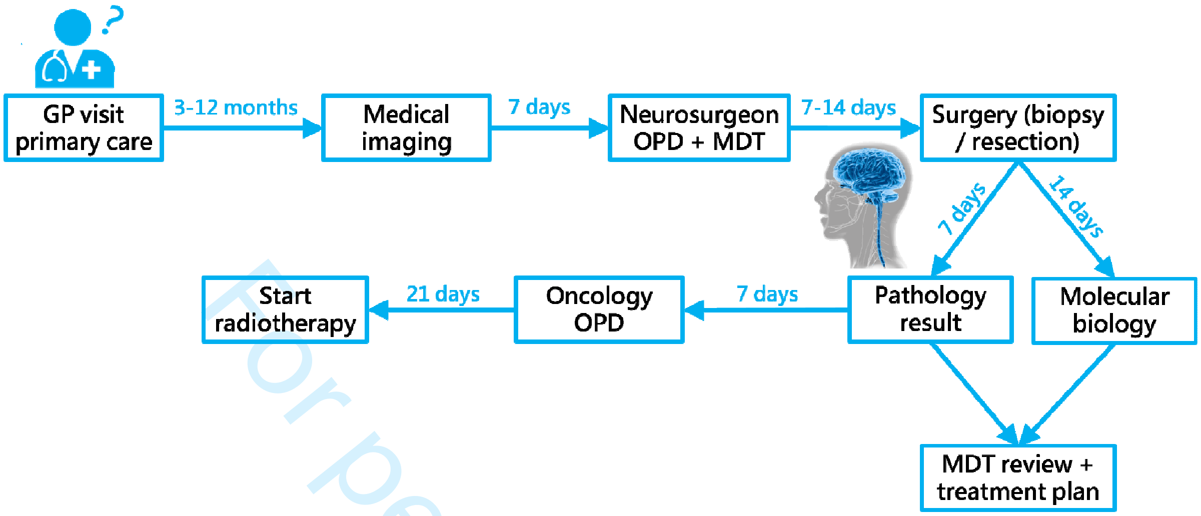

Figure 1. The diagnostic pathway of brain tumours. Timings relate to the diagnosis of high grade gliomas and are based upon discussion with the Clinical Focus Team and Aggarwal et al. (2015)[5]. (*OPD – Outpatient department; MDT – Multidisciplinary team*)

Serum Spectroscopic Diagnostics

This novel blood test for early brain tumour detection is based upon the interaction of infrared (IR) light with biological components of blood serum (**Figure 2**). Specifically, using attenuated total reflectance (ATR)-FTIR spectroscopy, specific bond vibrations of given molecules can be elucidated from serum samples, thus providing a unique insight into the composition via an absorbance spectrum.[17] Benefits of an ATR-FTIR based approach include a robust, user-friendly methodology without extensive sample preparation, that would readily fit into a clinical setting.[18] In short, serum is obtained according to standard protocols and can be snap frozen and stored at -80° until the point of analysis. A small volume of serum is required for analysis (1-5 μL), which is pipetted onto a crystal, known as an internal reflection element (IRE), where IR non-destructively interacts with the sample and produces an IR spectrum, with peaks representative of known bond vibrations and hence biomolecular constituents.

For peer review only

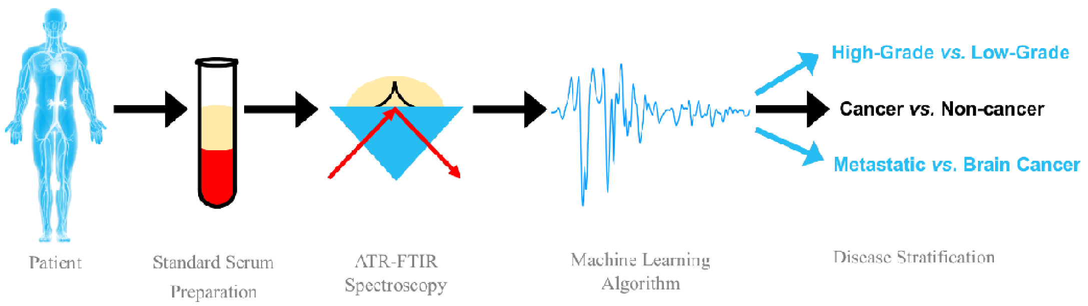

Figure 2. Attenuated total reflectance (ATR)-Fourier transform Infrared (FTIR) spectroscopic test of blood serum for the diagnosis and stratification of brain tumours using machine learning algorithms.

Blood serum is a complex medium that contains a variety of biomolecules, including around 20,000 proteins, that may be employed as diagnostic biomarkers.[19] In the case of brain tumours, such blood based technologies are limited, due to a lack of an established brain tumour specific diagnostic biomarker.[20] With our spectroscopic approach, rather than derive single biomarker specific information, a global signature is obtained, which encompasses the entire biomolecular makeup. This is epitomised as an equally complex biological absorbance spectrum, which contains a wealth of diagnostic information (an example may be seen in **Figure 3**).

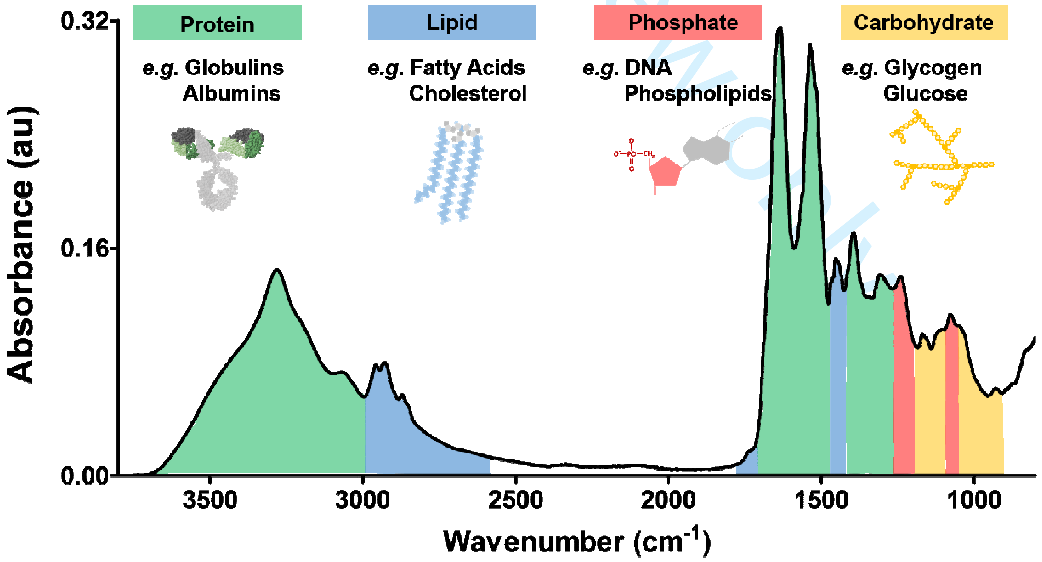

Figure 3. An unprocessed spectrum derived from human blood serum using ATR-FTIR spectroscopy. Spectral regions correspond to known bond vibrations and can therefore be associated with groups of biomolecules such as protein, lipid, phosphate and carbohydrates. Broad examples of blood serum constituents are listed.

Pattern recognition and machine learning algorithms using spectral features from FTIR data have been demonstrated as rapid and accurate for separating primary brain cancer and non-cancer cases.[2,21] When these algorithms are applied to this information rich dataset, the relationship between all biological components of the sample is ascertained, providing a multi-dimension analysis of the sample.

This approach has been able to not only detect between cancer and non-cancer, but also stratify based upon cancer pathology.[2] The ability to triage patients likely to have a brain tumour based on serum sample alone raises the possibility of systematic triaging prior to investigation with more expensive (MRI/CT imaging) and invasive (biopsy) tests. One major impact of having a serum test available would be a clear reduction in the number of unnecessary brain scans; however, as this test is also able to differentiate between primary and secondary tumours, there could also be a knock-on reduction of chest and abdomen scans which are conducted to rule out primary disease elsewhere. Ultimately, it is expected that this could allow earlier and potentially more effective treatment of brain tumour.

Aims & Objectives

The aim of the economic evaluation is to assess the potential cost-effectiveness of this spectroscopic technology, in advance of any prospective study results being available. There are three main objectives for the evaluation. The first objective is to create a map of where the test could be used in the clinical pathway. The second is to assess the potential cost-effectiveness of the technology, if the performance shown in the case-control study is replicated prospectively. This will give an indication of whether the technology would meet the criteria for acceptance for use in the National Health Service that are applied by Health Technology Assessment agencies (HTA). Related to this, the third objective is to define the level of performance in prospective trials, and any additional evidence that would be needed to meet the cost-effectiveness criteria of an HTA decision-making process. This can include diagnostic performance and also effects on long-term outcomes such as survival and resource use. To achieve these objectives a simplified economic model of two important clinical scenarios is used to explore cost effectiveness.

For peer review only

Methodology

Mapping the Clinical Pathway

In order to appreciate the current clinical pathway and determine an appropriate entry point for a serum spectroscopic test, a pan-UK clinical focus group was established. This cohort included neurosurgeons, clinical and medical oncologists, neuropathologists, neuroradiologists, academic GPs with special interests, and experts in primary care diagnostics (see Appendix 1).

Cost Effectiveness Analysis

A cost-effectiveness analysis (CEA) was conducted to calculate the effects on health outcomes and health service costs of introducing spectroscopic testing in each of two scenarios. The health outcomes considered were life-years and quality-adjusted life-years (QALYs).

A decision tree was used to model the pathway for patients presenting with symptoms warranting a referral for MRI/CT imaging for suspected brain tumour (**Figure 4**). Separate models were considered for primary and secondary care. The time horizon of the model is 2 years and the perspective is that of the health care service. A two-year horizon was selected because of the short duration of survival in this patient group: median survival is approximately 1 year for high grade gliomas, which are the commonest primary brain tumour. In all scenarios the comparator is the current diagnostic pathway (i.e. imaging alone). Further details regarding the node probabilities, simplifications and assumptions of the model can be found in **Appendix 2**.

For peer review only

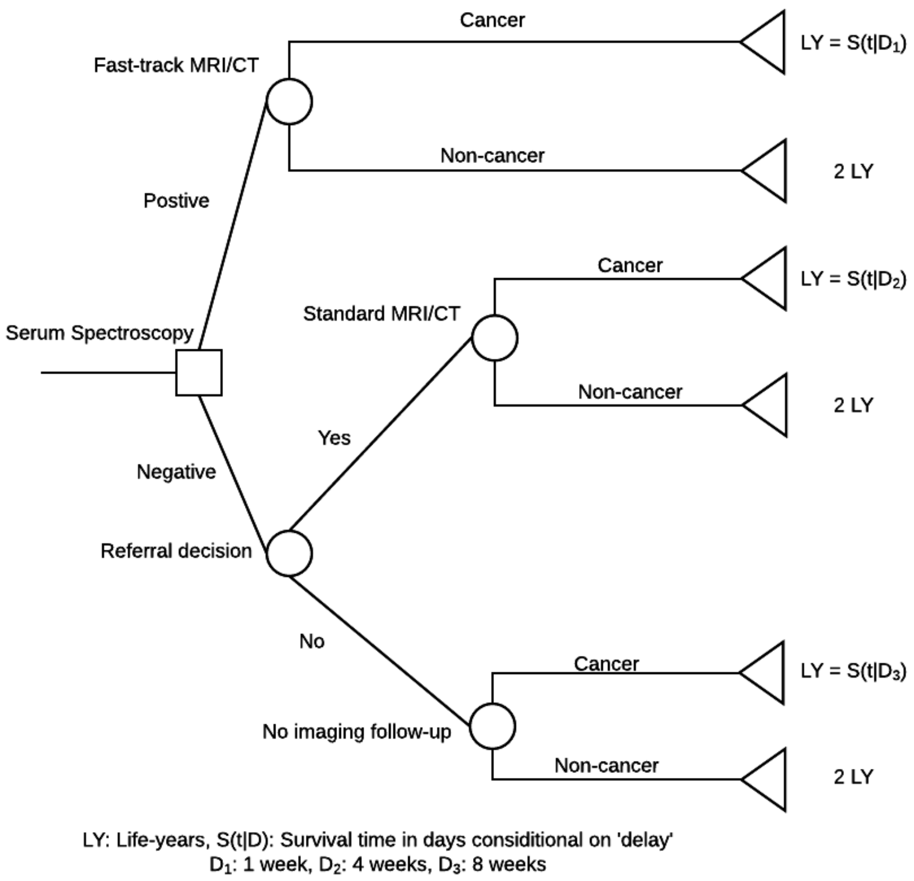

Figure 4. A decision tree model describing the integration of a serum spectroscopy test in the current diagnostic pathway, and the effect on MRI/CT imaging for suspected brain tumour.

Diagnostic Performance

The sensitivity and specificity of the test for detecting brain tumours has been demonstrated in a series of case-control studies using historical samples from the Brain Tumour North West and Walton Centre NHS bio-banks.[2,3] In the key case-control study nine FTIR spectra were collected from each of 433 patients.[3] Of these 134 were from patients with primary brain tumours (64 high grade glioma), 177 were from patients with cerebral metastases and 122 were from non-cancer controls. FTIR spectra were analysed using the random forest method to fit a classification model. Classification performance was estimated by applying the fitted model to a test set containing 20% of the patients from the original data set that were not used in the model fitting step (hold-out test set). Classification statistics are computed as averages of this process, iterated 96 times using random training and test sets. Under the best available model, sensitivity estimated by this method was 92·8% and specificity was 91·5% for the analysis of cancerous vs. non-cancerous serum.[3]

Establishing whether the performance demonstrated case-control data from historical samples translates to equivalent performance when applied prospectively in clinical practice is the subject of a planned clinical trial. This is critical to demonstrating the clinical and cost-effectiveness of a serum spectroscopy test as part of the diagnostic pathway for brain tumours.

Prevalence of disease

Prevalence data were sourced from the literature based on clinical expert guidance. Brain tumour prevalence in scenario 2 (secondary care) was assumed to be 3% based on observed rates of primary brain tumour diagnosis among patients referred for brain imaging for suspected cancer in secondary care.[22-24] In scenario 1 (primary care) an estimated prevalence of 0·5% is used based on case-control evidence and expert opinion of the prevalence among patients who would be considered for direct access imaging, using MRI or CT, where this is available and referral to neurology where it is not.[25,26] An alternative prevalence of 1% was explored for scenario 1 based on unpublished data from a direct access imaging service in the UK (P. Brennan, personal communication).

The effect of serum spectroscopy testing on the time-to-diagnosis and time-to-treatment is discussed in Appendix 3, additional to the effect of testing on the use of imaging studies, and also on the patient outcomes. The primary assumptions in this model are that first, the expected time-to-diagnosis would match the current median time-to-diagnosis for patients presenting with brain tumour in emergency care as observed in Aggarwal et al.[5] Furthermore, based upon expert opinion, it is assumed that in secondary care all patients would continue to imaging, while in primary care 50% would continue to imaging following a negative spectroscopy result. Finally, the effects of early diagnosis on the outcome of brain tumours is estimated using literature describing fitting natural history models to observational datasets of high grade glioma patients.[27,28]

Utility Weights

Health state utility weights are applied to life-years to generate quality adjusted life years (QALYs). A systematic review of health state utility weights for high grade glioma, the most common and aggressive primary brain tumour, was conducted to identify suitable utility weights. Due to heterogeneity it was not considered suitable to pool the estimates. The most appropriate health state utility weight was taken from a previous UK economic evaluation of glioblastoma treatment.[29] A value of 0·89 was used in the base case.

Resource Use and Costs

Resource use includes the application of a spectroscopic serum test to all patients prior to imaging, the imaging studies used in the diagnostic process, outpatient neurology clinic visits and general practitioner visits. In the UK analysis, unit costs for imaging studies are taken from UK NHS reference costs (2014/15), clinic and GP visits from the PSSRU costs schedule (Table 1). In the USA analysis, unit costs are taken from Medicare reimbursement schedules. Unit costs of the test were applied at an upper bound and lower bound rather than a single value as these products have not yet been commercialized. Bounds were set by consultation

For peer review only

with scientists developing the tests. Additional resource use and cost assumptions are described in Appendix 3.

Table 1. Unit costs and comparison for the brain tumour diagnostic pathway

Item	UK Cost per unit (£) (2015 prices)	USA cost per unit ($) (2016 prices)	Source(s)
CT imaging study [CT head]	85	163	National Schedule of Reference Costs (2014 – 15), Medicare Physician Fee Schedule 2016
MRI imaging study [MRI brain with contrast]	164	380	National Schedule of Reference Costs (2014 – 15), Medicare Physician Fee Schedule 2016
Neurology outpatient appointment	35	76	PSSRU 2016, Medicare Physician Fee Schedule 2016
GP visit	47·25	76	PSSRU 2016, Medicare Physician Fee Schedule 2016
Stable disease monitoring costs	116 per 3 months	154	[29], exchange rate adjusted
Serum spectroscopy test	Lower limit: 50<br>Upper limit: 100	Lower limit: 100<br>Upper limit: 200	Assumed prices

Sensitivity Analysis

Incremental cost effectiveness ratios (ICER) were calculated for scenarios 1 and 2 using the base case parameter estimates. Base case analysis was repeated for UK and USA settings. Additional sensitivity analyses are reported for the UK setting only.

Sensitivity analyses included one-way sensitivity analysis (OWSA), systematically varying a single parameter in the model, and scenario analysis in which specific model assumptions were altered. OWSA were conducted for test sensitivity, specificity and test cost. Scenario analyses included assuming an additional consultation cost for discussion of test results,

For peer review only

assuming a higher proportion of patients continue to imaging following a negative spectroscopy result in primary care, and using mean survival rather than median survival.

A probabilistic sensitivity analysis (PSA) was conducted to explore the effects of joint uncertainty in the parameter estimates on the model results.[30]

For peer review only

Results

Mapping the clinical pathway

For the first study objective, initial discussion with clinical experts indicated that there are potential uses for the test in both primary and secondary care. The main advantage of employing a cost-effective spectroscopic blood test in the diagnostic pathway is to use it as a triage test. This prioritises more urgent cases for access to services, and acts as a gate-keeper (requiring a positive result in some conditions to give access to services) for imaging studies.

Two clinical scenarios are mapped out below and subsequently explored in this early economic evaluation of the serum spectroscopy test for aiding diagnosis of brain tumours:

1. Triage tool in primary care

The primary care scenario explores a population of patients with a clinical presentation that warrants further investigation of possible brain tumour. This would include some patients with headaches and some with focal neurological deficits. This is the group of patients who would be considered for direct access imaging, using MRI or CT, where this is available and referral to neurology where it is not.[31] The blood test is used to provide rapid information, within 24 hours, where a positive result would lead to patients receiving more timely access to imaging. It may also be the case that negative test results, in addition to establishing the low probability of a brain tumour, could also provide some reassurance for those patients that must wait for imaging. The total volume of tests would be approximately 75,000 per year in the UK (see Appendix 4 for further details).

2. Triage tool in secondary care

In this scenario the population is the group of patients that are currently referred for imaging studies from secondary care for suspected brain tumour, typically via neurology clinics. This is the patient group for whom the clinical presentation has the highest positive predictive value (PPV). However, even in this high risk group, the odds of a brain tumour being present are approximately 1:33.[22-24] Again, the spectroscopy test is used to provide rapid information to allow a subset of these patients to access immediate imaging and provide reassurance to other patients who may have to wait longer for definitive imaging studies and diagnosis. The extent of the benefits of triage in this scenario is likely to vary by locality depending on the capacity constraints on imaging and pathology services. This evaluation uses estimates of the delays in diagnosis, and potential improvements in the speed of diagnosis, from a consecutive patient case series in London, UK.[5] The total volume of tests if this scenario occurred would be approximately 53,000 per year in the UK (see Appendix 4 for further details).

For peer review only - http://bmjopen.bmj.com/site/about/guidelines.xhtml

Cost Effectiveness Assessment

The standard threshold value per QALY gained in the UK, is considered to be between £20,000 to £30,000. Below this value, a healthcare intervention may be considered cost effective, whereas a negative ICER value would be deemed cost saving. Base case results for primary care (scenario 1) and secondary care (scenario 2) are presented in Table 2. Note results are reported for cohorts of 10,000 patients.

Table 2. Incremental QALYs, costs and ICERs for Scenarios 1 and 2, UK and USA

	Scenario 1 – Primary Care			Scenario 2 – Secondary Care		
**Serum spectroscopy test cost (£)**	**ΔQALY**	**ΔCost**	**ICER**	**ΔQALY**	**ΔCost**	**ICER**
**UK**						
**50**	8·81	-422,116	-47,913 (dominates)	52·86	527,646	9,982
**100**	8·81	77,884	8,840	52·86	1,027,646	19,441
**USA**						
**100**	8·81	-1,718,475	-195,058 (dominates)	52·86	536,702	10,153
**200**	8·81	-218,475	-24,798 (dominates)	52·86	2,036,702	38,530

ΔQALY, ΔCost: difference is QALYs/Costs (with serum spectroscopy test – without test), 10K patients
ICER: Incremental cost-effectiveness ratio

Incremental cost-effectiveness ratios were well below standard threshold values of £20,000 to £30,000 per quality adjusted life year (QALY) gained used in the UK, and similar thresholds used internationally, provided the test cost did not exceed £100. The base case results demonstrate that the serum spectroscopy test dominates (more effective and less costly) standard care at the lower bound of test cost in the primary care setting in both the UK and USA. At the upper bound of test cost the ICERs may be within commonly used thresholds or, in the case of the USA, remain dominant to standard care. In the secondary care setting ICERs of £9,982 and $10,153 at the lower bounds of test cost indicate that this test is potentially cost-effective in this setting. At the upper bounds ICERs may still be within commonly used thresholds for cost-effectiveness.

Sensitivity Analysis Results

The performance of the test with regards to levels of sensitivity and specificity are addressed using sensitivity analysis. OWSA results for a range of test specificities are displayed in

Figure 3 and Figure 4, for primary and secondary care respectively, displaying the ICER with varying test specificity. Note that the estimated QALYs do not change with specificity in the model therefore changes in the ICER are due solely to changes in incremental costs. Varying sensitivity changes both estimated QALYs and estimated costs therefore results of the OWSA for test sensitivity are presented on the cost-effectiveness plane (Appendix 5). In primary care, using the upper cost limit of £100 it is evident that the test is deemed cost effective at specificities of around 0·9 and above, where the ICER is below standard thresholds. In contrast, at the lower cost limit, the test is cost effective at specificities above 0·8. Although the serum spectroscopy test is not cost saving at low cost (or near perfect specificities), the test is still considered cost effective at specificity levels around 0·7 and 0·8 for £50 and £100 pricing respectively.

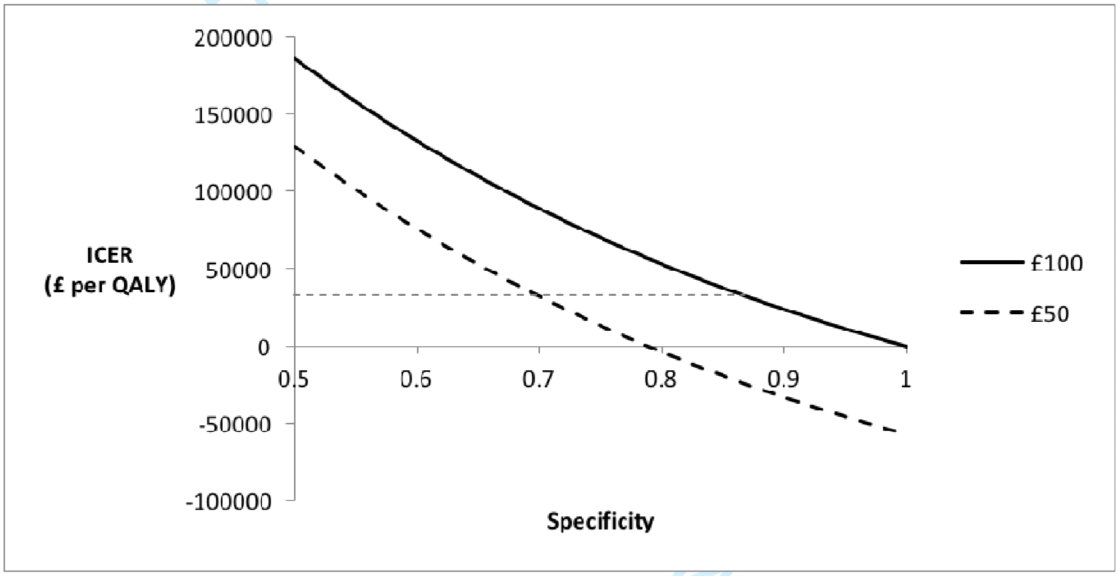

Figure 5 – Incremental cost effectiveness ratios (ICERs) at various specificities in primary care (‘Scenario 1’). The £30,000 ICER threshold is displayed as a dashed horizontal line.

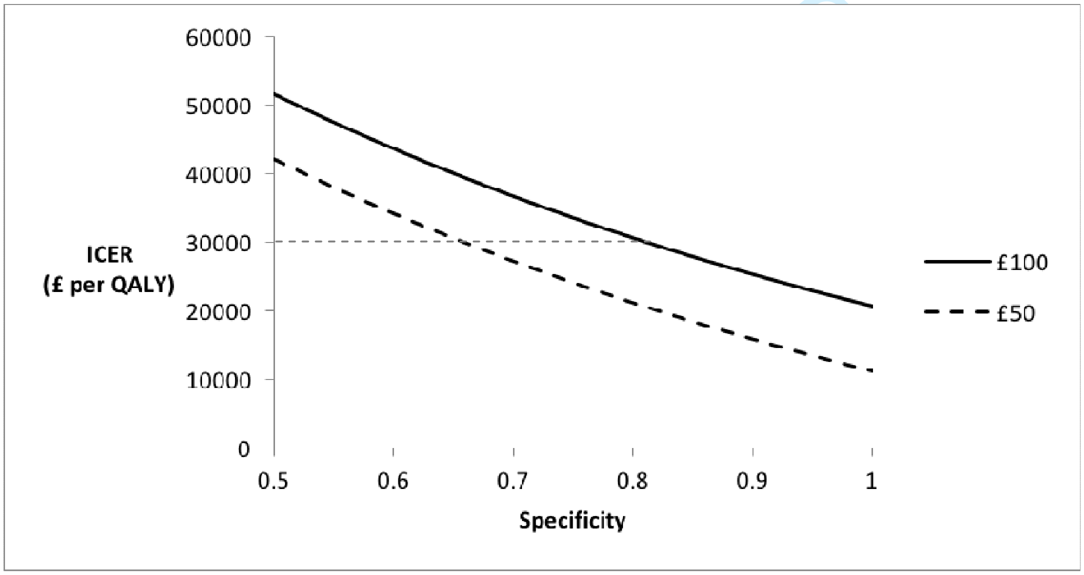

Figure 6 - Incremental cost effectiveness ratios (ICERs) various specificities in Secondary Care ('Scenario 2'). The £30,000 ICER threshold is displayed as a dashed horizontal line.

The OWSA results highlight how ICERs in scenario 2 (secondary care) are strongly influenced by test sensitivity while ICERs in scenario 1 (primary care) are more strongly influenced by test specificity. These features are a result of the varying prevalence of disease and the assumption in scenario 2 that 100% of patients are referred for imaging following negative result. It should be noted that test specificity is important in both scenarios. Relatively small improvements in test specificity can substantially change the ICER, while larger improvements in test sensitivity are required to substantially alter the ICERs.

Additional scenario analyses are also reported in Appendix 5. These demonstrate that results are robust to using mean survival estimates rather than median survival estimates and including additional consultation costs for positive test results. If the prevalence of brain tumours in Scenario 1 is 1% rather than 0·5% the incremental QALYs increase substantially and the ICERs are reduced.

The PSA results reported in Figure 5 and 6 indicate that at a test cost of £50 and an ICER threshold of £30,000 per QALY there is a near 100% probability the serum spectroscopy test is cost-effective in scenario 1 and approximately 90% probability it is cost-effective in scenario 2. The corresponding probabilities at the upper bound cost of £100 are approximately 85% and 75%.

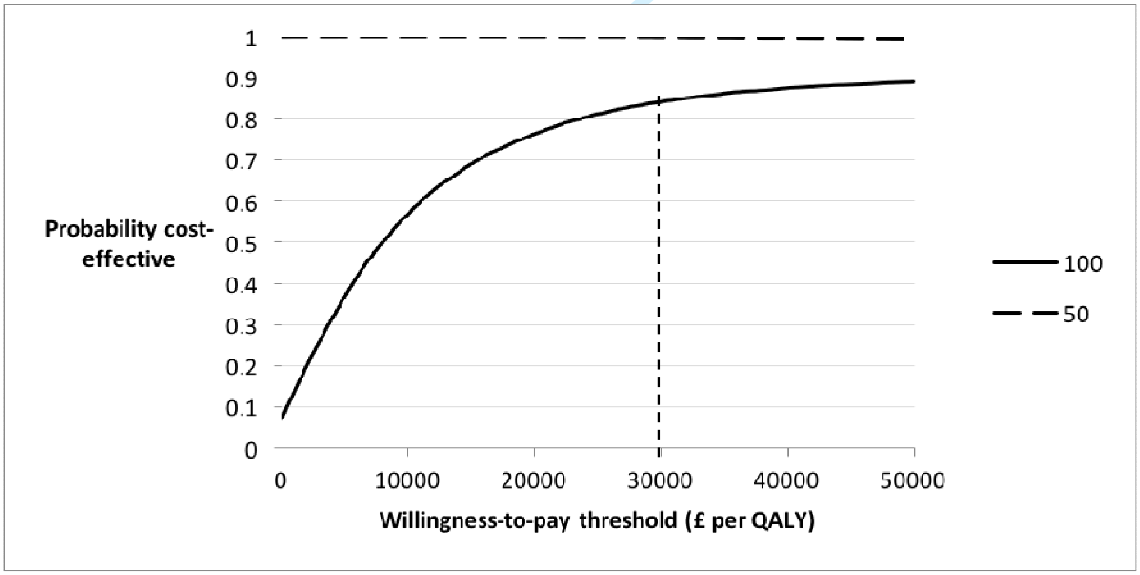

Figure 7 – Cost effectiveness acceptability curve (CEAC) at £50 and £100 per test – Primary care ('Scenario 1')

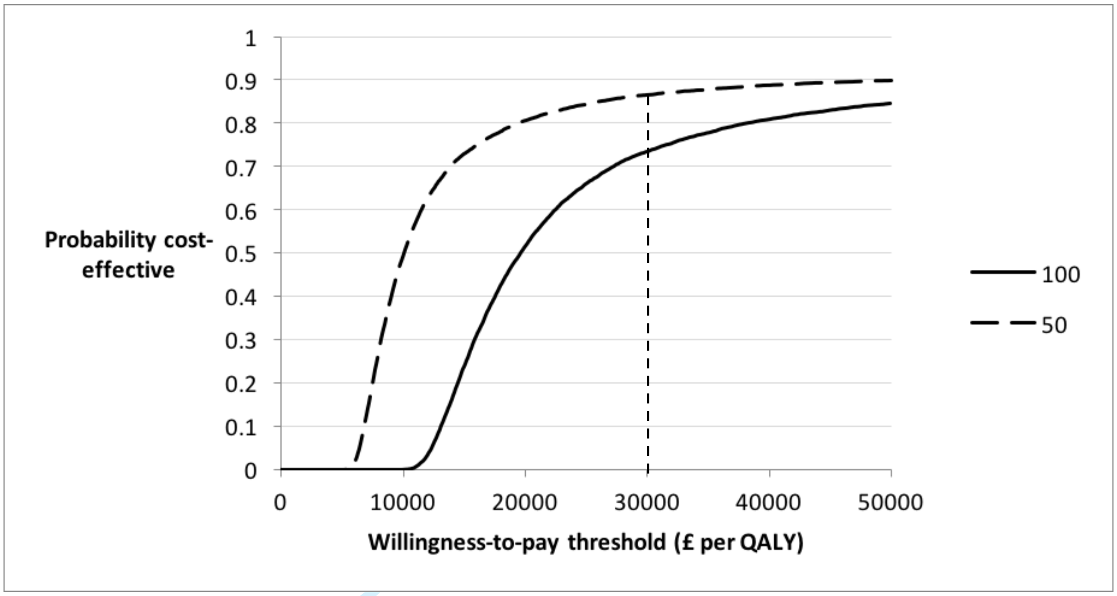

Figure 8 – Cost effectiveness acceptability curve (CEAC) at £50 and £100 per test – Secondary care ("Scenario 2")

For peer review only - http://bmjopen.bmj.com/site/about/guidelines.xhtml

Discussion

This economic evaluation establishes the potential for serum spectroscopy to have a role in the diagnosis of both benign and malignant brain tumours in both primary and secondary care. The potential costs and health benefits of testing using a spectroscopic method prior to CT/MRI tests (or in some scenarios to avoid imaging) have been estimated based on a mathematical model with parameter values taken from published studies and expert opinion. This diagnostic tool is sensitive to all brain tumours (benign or malignant), however, this assessment is closely aligned with the diagnosis of primary gliomas, where there is a maximum potential benefit to the health service.

When used as a triage tool in primary care, this novel test has the potential to deliver improvements in health outcomes and also to reduce costs. At the lower end of test costs, the technology would be cost-saving for the health service. At higher test costs the technology is still likely to be considered cost-effective in HTA agency decision processes.

In Scenario 2, in which serum spectroscopy is used as a triage tool in secondary care, the technology will create additional costs but also produce sizable health benefits. At test costs of under £100 ($200) the technology would be likely to be considered as a cost-effective use of resources in HTA agency decision processes in the UK (and USA).

Sensitivity analyses have demonstrated the importance of diagnostic performance on the cost-effectiveness of the test. In particular, test specificity is important in the primary care setting. If test specificity is 87·5% or worse the technology may not be considered cost-effective at higher values of assumed test cost. This is due to the increased number of false positive results in this low prevalence population, generating a greatly increased proportion of fast-track imaging studies which increases costs.

To strengthen the case that this approach represents a cost-effective use of healthcare resources it is necessary to establish the diagnostic performance of the test prospectively. This can be accomplished by a suitably large cohort study in which serum spectroscopy is used alongside current clinical practice in one of the patient groups included in this evaluation. It would be appropriate to initially target the secondary care patient population, because the higher prevalence of disease in this group will reduce the sample size needed to accurately estimate diagnostic performance.

Decision makers are often most interested in patient outcomes, such as survival, rather than intermediate outcomes, such as accuracy or speed of diagnosis (although this latter point is vital for treatment of high grade gliomas). From this perspective, randomised, or a prospective cohort trial with extended follow-up, may be required to fully establish the size of survival and quality-of-life benefits of including a serum spectroscopy test in the diagnostic pathway. A trial with primary outcomes relating to survival and quality-of-life would be specific to either the primary or secondary care setting (rather than generalisable to

both), would need a large sample size, and would also require a follow-up period to capture survival benefits. In the case of malignant glioma this would require a period of at least 24 months. Such a trial would clearly be expensive, time consuming and may be unfeasible. Decision makers may be willing to make a decision on implementation of the blood test based on the modeled effects of improvements in intermediate outcomes on later patient outcomes. In this situation, the model proposed in this evaluation, populated with diagnostic performance and other data from a prospective trial, could be used to inform decisions about the wider adoption of the technology.

Several results in this analysis suggest cost savings through reduced use of imaging for patients with a negative test result. To make the case that a serum spectroscopic test can improve the efficiency of the diagnostic pathway prospective studies will also need to explore the impact of these test results on clinician and patient imaging study decisions. The possibility remains that the test may triage patients, but may not reduce the number of scans being conducted, and could potentially increase the demand on imaging. However, this triaging of patients would still benefit each patient that is provided with an early diagnosis.

This evaluation has explored the potential for serum spectroscopy to be a cost-effective addition to the diagnostic pathway for brain tumours. It has demonstrated that in specific scenarios this novel test may be an effective and cost-effective technology in reducing the delay to diagnosis for patients with brain tumours. Prospective trials are required to provide definitive evidence.

For peer review only

References

1 Burnet, N., Jefferies, S., Benson, R., Hunt, D. & Treasure, F. Years of life lost (YLL) from cancer is an important measure of population burden—and should be considered when allocating research funds. *Brit J Cancer* 2005; **92**: 241–5.

2 Hands, J. R., Clemens, G., Stables, R. *et al.* Brain tumour differentiation: rapid stratified serum diagnostics via attenuated total reflection Fourier-transform infrared spectroscopy. *J Neurooncol* 2016; **127**: 463–72.

3 Smith, B. R., Ashton, K. M., Brodbelt, A. *et al.* Combining random forest and 2D correlation analysis to identify serum spectral signatures for neuro-oncology. *Analyst* 2016; **141**: 3668–78.

4 Finding a better way? Improving the quality of life for people affected by brain tumours, Report Published by The Brain Tumour Charity, 2014.

5 Aggarwal, A., Herz, N., Campbell, P. *et al.* Diagnostic delay and survival in high-grade gliomas - evidence of the 'waiting time paradox'? *Br J Neurosurg* 2015; **29**: 520–3.

6 Zhou, Y., Abel, G. A., Hamilton, W. *et al.* Diagnosis of cancer as an emergency: a critical review of current evidence. *Nat Rev Clin Oncol* 2017; **14**: 45–56.

7 McPhail, S., Elliss-Brookes, L., Shelton, J. *et al.* Emergency presentation of cancer and short-term mortality. *Brit J Cancer* 2013; **109**: 2027–34.

8 Blanks, R., Moss, S., McGahan, C., Quinn, M. & Babb, P. Effect of NHS breast screening programme on mortality from breast cancer in England and Wales, 1990-8: comparison of observed with predicted mortality. *BMJ* 2000; **321**: 665–9.

9 UK Colorectal Cancer Screening Pilot Group. Results of the first round of a demonstration pilot of screening for colorectal cancer in the United Kingdom. *BMJ* 2004; **329**: 133.

10 Quinn, M., Babb, P., Jones, J. & Allen, E. Papers Effect of screening on incidence of and mortality from cancer of cervix in England: evaluation based on routinely collected statistics. *BMJ* 1999; **318**: 904.

11 Frankel, S., Smith, G. D., Donovan, J. & Neal, D. Screening for prostate cancer. *The Lancet* 2003; **361**: 1122–8.

12 Gadalla, S., Pfeiffer, R., Kristinsson, S. *et al.* Brain tumors in patients with myotonic dystrophy: a population□based study. *Eur J Neurol* 2016; **23**: 542–47.

13 Callaghan, B. C., Kerber, K. A., Pace, R. J., Skolarus, L. E. & Burke, J. F. Headaches and neuroimaging: high utilization and costs despite guidelines. *JAMA Intern Med* 2014; **174**: 819–21.

14 National Institute for Health and Care Excellence, 2015. Suspected cancer: recognition and referral. NICE Guideline (MG12).

15 Livermore, L. J., Ma, R., Bojanic, S. & Pereira, E. A. Yield and complications of frame-based and frameless stereotactic brain biopsy–the value of intra-operative histological analysis. *Brit J Neurosurg* 2014; **28**: 637–44.

16 Weber, M. A., Zoubaa, S., Schlieter, M. *et al.* Diagnostic performance of spectroscopic and perfusion MRI for distinction of brain tumors. *Cancer Imaging* 2006; **6**: S32–S41.

17 Baker, M. J., Trevisan, J., Bassan, P. *et al.* Using Fourier transform IR spectroscopy to analyze biological materials. *Nature Protoc* 2014; **9**: 1771–91.

18 Lovergne, L., Bouzy, P., Untereiner, V. *et al.* Biofluid infrared spectro-diagnostics: pre-analytical considerations for clinical applications. *Faraday Discuss* 2016; **187**: 521–37.

19 Baker, M. J., Hussain, S. R., Lovergne, L. *et al.* Developing and understanding biofluid vibrational spectroscopy: a critical review. *Chem Soc Rev* 2016; **45**: 1803–18.

20 Hochberg, F. H., Atai, N. A., Gonda, D. *et al.* Glioma diagnostics and biomarkers: an ongoing challenge in the field of medicine and science. *Expert Rev Mol Diagn* 2014; **14**: 439–52.

21 Hands, J. R., Abel, P., Ashton, K. *et al.* Investigating the rapid diagnosis of gliomas from serum samples using infrared spectroscopy and cytokine and angiogenesis factors. *Anal Bioanal Chem* 2013; **405**: 7347–55.

22 Wang, H. Z., Simonson, T. M., Greco, W. R. & Yuh, W. T. C. Brain MR Imaging in the Evaluation of Chronic Headache in Patients without Other Neurologic Symptoms. *Acad Radiol* 2001; **8**: 405–8.

23 Sempere, A. P., Porta-Etessam, J., Medrano, V. *et al.* Neuroimaging in the evaluation of patients with non-acute headache. *Cephalalgia* 2005; **25**: 30–5.

24 Clarke, C. E., Edwards, J., Nicholl, D. J. & Sivaguru, A. Imaging results in a consecutive series of 530 new patients in the Birmingham Headache Service. *J Neurol* 2010; **257**: 1274–9.

25 Kernick, D., Stapley, S., Goadsby, P. J. & Hamilton, W. What happens to new-onset headache presented to primary care? A case-cohort study using electronic primary care records. *Cephalalgia* 2008; **28**: 1188–95.

26 Hamilton, W. & Kernick, D. Clinical features of primary brain tumours: a case–control study using electronic primary care records. *Brit J Gen Pract* 2007; **57**: 695–9.

27 Do, V., Gebski, V. & Barton, M. The effect of wating for radiotherapy for grade II/IV gliomas. *Radiother Oncol* 2000; **57**: 131–6.

28 Burnet, N., Jefferies, S., Benson, R., Hunt, D. & Treasure, F. Years of life lost (YLL) from cancer is an important measure of population burden—and should be considered when allocating research funds. *Brit J Cancer* 2005; **92**: 241–45.

29 Garside, R., Pitt, M., Anderson, R. *et al.* The effectiveness and cost-effectiveness of carmustine implants and temozolomide for the treatment of newly diagnosed high-grade glioma: a systematic review and economic evaluation. *Health Technology Assessment* 2007; **11**.

30 National Institute for Health and Care Excellence, 2013. Guide to the methods of technology appraisal. NICE Guideline (PMG9).

31 Simpson, G. C., Forbes, K., Teasdale, E., Tyagi, A. & Santosh, C. Impact of GP direct-access computerised tomography for the investigation of chronic daily headache. *Br J Gen Pract* 2010; **60**: 897–901.

32 Taylor, T., Evangelou, N., Porter, H., Hamilton, W. & Kernick, D. Headache: two views on the right approach in general practice. *Br J Gen Pract* 2014; **64**: 475–6.

33 Taylor, T. R., Evangelou, N., Porter, H. & Lenthall, R. Primary care direct access MRI for the investigation of chronic headache. *Clin Radiol* 2012; **67**: 24–7.

34 White, P., Halliday-Pegg, J. & Collie, D. Open access neuroimaging for general practitioners - diagnostic yield and influence on patient management. *Brit J Gen Pract* 2002; **52**: 33–35.

35 UK Statistics Authority. Cancer Registration Statistics, England, 2014. Office of National Statistics

For peer review only

Web Extra Material

Appendix 1 – Clinical expert group

Clinical expert group participants: Mr Michael D Jenkinson (Walton Centre NHS Trust & University of Liverpool), Dr Paul Brennan (University of Edinburgh), Prof. Anthony Chalmers (University of Glasgow & Beatson Centre), Prof. Willie Hamilton (University of Exeter Medical School), Mr Kevin O'Neill (Imperial College Healthcare NHS Trust), Dr Allan Janes (Beatson West of Scotland Cancer Centre), Dr Babar Vaqas (Imperial College Healthcare NHS Trust), Dr David Kernick (St Thomas Health Centre, Exeter), Dr James Livermore (University of Oxford), Dr Ruth Board (Lancashire Teaching Hospitals NHS Trust), Prof. Timothy Dawson (Lancashire Teaching Hospitals NHS Trust), Miss Elvira Lekka (Lancashire Teaching Hospitals NHS Trust), Mr John Goodden (Leeds Teaching Hospital NHS Trust) and Dr. Samantha Mills (Walton Centre NHS Trust)

For peer review only

Appendix 2 – Decision tree model

The decision node probabilities that populate the decision tree model are reported in Table S1. The model is simplified by assuming that the reference test (MRI/CT) has perfect accuracy (100% sensitivity and 100% specificity). This is desirable if we wish to evaluate the test against an MRI/CT only diagnostic pathway in which these imaging studies are considered as the 'gold standard'. It is also difficult to obtain valid estimates of MRI/CT sensitivity and specificity for this indication as these are not reported in a comparable manner in the literature. This simplification will not substantially alter the economic results compared to using more realistic values for the scenarios under consideration. This is because it is assumed confirmation of the diagnosis always requires imaging and therefore outcomes for patients that would hypothetically be classified correctly using this test but incorrectly by MRI/CT will remain the same whether or not the test is used. The effect of less than perfect accuracy of the reference test is therefore the same as a (small) decrease in prevalence as a proportion of patients with the disease cannot benefit from the additional test due to incorrect subsequent testing. Outcomes are calculated by 'rolling-back' the tree for both cancer cases and non-cases and taking an average of the two groups weighted by the prevalence of brain tumours in the scenario population.

Table S1. Decision node probabilities for decision tree model describing the integration of a serum spectroscopy test in the current diagnostic pathway

**Cancer cases:**	**Probability to upper branch**	**Probability to lower branch**
Serum Spectroscopy Test (test sensitivity)	0·928	0·072
Standard MRI/CT (reference test sensitivity)	1	0
Fast-track MRI/CT (reference test sensitivity)	1	0
Referral decision	S1:0·5, S2: 1	S1:0·5, S2: 0
Non-cases:		
Serum Spectroscopy Test (test specificity)	0·085	0·915
Standard MRI/CT (reference test specificity)	0	1
Fast-track MRI/CT (reference test specificity)	0	1
Referral decision	S1:0·5, S2: 1	S1:0·5, S2: 0

S1 & S2: Scenarios 1 &2

For peer review only

Appendix 3 – Assumptions and details of cost effective analysis model

Effect of testing on time-to-diagnosis and time-to-treatment
To estimate the expected time-to-diagnosis under a fast-track pathway it is assumed that this would match the current median time-to-diagnosis for patients presenting with brain tumour in emergency care as observed in Aggarwal et al [5]. This study reported a cohort of high grade glioma patients from a single hospital in London, UK. No other suitable estimates of time-to-treatment or time-to-diagnosis were identified in the literature. This is the most common primary brain tumour in the indications considered in this evaluation. Median time-to-diagnosis for the standard pathway is also taken from the same source.

Effect of testing on use of imaging studies
The effect of spectroscopic testing on imaging study decisions of clinicians and patients is uncertain. Based on clinical expert input it was assumed, in the base case, that in secondary care all patients would continue to imaging, while in primary care 50% would continue to imaging following a negative spectroscopy result.

Effect of early diagnosis on patient outcomes
A systematic review of the literature failed to identify any studies that directly estimated the effect of earlier (or later) diagnosis on outcomes for primary brain tumours. Instead, based clinical expert guidance, estimates of the effects of time-to-treatment were sourced from the literature. The estimated effects are based on fitting natural history models to observational datasets of high grade glioma patients.[27,28] It is necessary to use a natural history model to produce survival estimates rather than using the survival data stratified by time-to-treatment directly, in order to adjust for potential confounding factors influencing both time-to-treatment and survival.

A one-to-one correspondence is assumed for the effects of an additional day added to the time-to-diagnosis and an additional day added to the time-to-treatment. Median survival by weeks between diagnosis and treatment are displayed in Table 2. The hazard ratio per additional day delay calculated in Do et al of 1·015 was applied to each day between diagnosis and treatment up to 28 days to calculate median survival time. Time between diagnosis and treatment beyond 28 days were fixed at the same median survival as 28 days.

Table S2. Relationship between time-to-diagnosis and survival

Time-to-diagnosis (weeks)	Median survival (weeks)
0	46
1	41·4
2	37·3
3	33·6
4	30·3

Source: Do et al 2000

Resource use and cost

It is assumed that there is no additional cost for converting a small proportion of standard referral to additional urgent referrals. This could be justified on the basis that patients receiving negative results allows more flexibility in the scheduling for these patients. This could create more opportunity to schedule patients with positive spectroscopy results sooner. As a larger proportion of patients are referred for immediate imaging it was assumed costs would increase because achieving this would require additional capacity. It was assumed that unit costs for MRI and CT grow exponentially with increasing proportion of immediate referrals such that costs double if 50% of patients are referred for immediate imaging.

Treatment costs are assumed to be the same for both fast-track and standard referrals that are diagnosed as cases. This may be a conservative assumption as treatment costs are likely to increase as disease progresses and therefore earlier treatment may, on average, be less costly than later treatment. However, it is also possible that

For peer review only

treatment costs could be increased if some cases are converted from palliative care to more aggressive treatment. The balance of these effects is unknown given the lack of appropriate data to estimate the effects. Secondary treatment and end-of-life costs are similarly assumed to be equal between fast track and standard referrals. This reflects the model assumption that all patients ultimately progress.

The time intervals considered (0-4 weeks) are too short for discounting to have an important impact on cost estimates therefore the effects of moving forward treatment on present value is not considered.

For peer review only

Appendix 4 - Total demand for serum spectroscopy tests in UK in two clinical scenarios

1. Primary care

An assumption that patients currently referred in direct-access imaging service would be selected for spectroscopic testing can provide a conservative estimate of the total number of patients likely to be tested. Three sources from the literature were considered:

1. A direct-access brain MRI service in Nottingham (UK) reported 130 patients per quarter (520 per year) among a population of 342,000.[32,33] The population of the UK is approximately 61 million therefore, assuming Nottingham is representative of the UK general population demand for brain MRI, there would be approximately 93,000 per year in the UK.
2. A direct access CT service for chronic headache in Glasgow (UK) reported 4404 patients referred over 8 years (551 per year) among a population of approximately 1 million.[31] Assuming this was representative of the UK generally then the demand for this type of service would be approximately 34,000 for the UK.
3. A direct access CT neuroimaging service in Lothian (UK) reported 389 exams per year for a population of approximately 442,000.[34] Assuming this was representative of the UK generally then the demand for this type of service would be approximately 54,000 for the UK.

The Glasgow estimate is particularly low and may result from limited uptake in the early part of the 8 year period for which data were reported. As it was not possible to determine if uptake increased over the period or was stable this estimate was not considered reliable. The Nottingham and Lothian estimates report potentially more stable and established services and are therefore more likely to reliably estimate demand. The likely demand for this novel test, if the indication is considered identical to neuroimaging, is therefore between 54,000 and 93,000. This is likely to be conservative as the test may well have a slightly broader indication than current neuroimaging. A reasonable point estimate may be approximately 75,000.

2. Secondary Care

An estimate of the total demand for serum spectroscopic tests in the UK can be made based on the reported incidence of brain tumour in the UK and the proportion of cases currently diagnosed through secondary (non-emergency) care. Annual incidence of malignant brain tumours in the UK is approximately 4,700.[35] Approximately one third of these are likely to present through secondary, non-emergency care. Using the positive predictive value for patients currently referred to neuroimaging in this setting of 3% suggests that for each case there will be 33 non-case patients receiving neuroimaging. In total, in the UK, this would imply 53,000 patients per year in secondary care may be suitable for spectroscopic testing.

For peer review only

Appendix 5 - Sensitivity Analysis

One-way sensitivity analysis (OWSA)

OWSA results for a range of test sensitivity are displayed in S1 and S2 as a series of points on a cost-effectiveness (CE) plane with incremental QALYs (intervention-control) on the x-axis and incremental costs (intervention - control) on the y-axis. Corresponding OWSA results for a range of test specificities in primary and secondary care are shown in Figure 3 and 4 in the main article text respectively. These are displayed with the ICER on the y-axis and the test specificity on the x-axis. Note that the estimated QALYs do not change with specificity in the model therefore changes in the ICER are due solely to changes in incremental costs.**Error! Reference source not found.** All OWSA analyses show results assuming the spectroscopy based test costs of £50 and £100.

Figure S1 – Results on CE plane over a range of test sensitivities (0·5-0·99) – Primary care

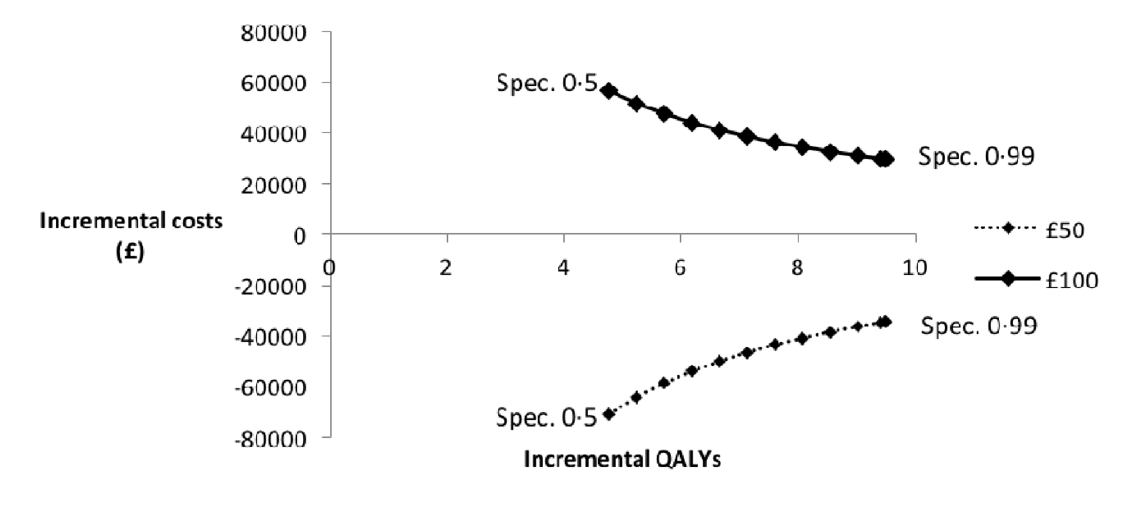

Figure S2 - Results on CE plane over a range of test sensitivities (0·5-0·99) – Secondary care

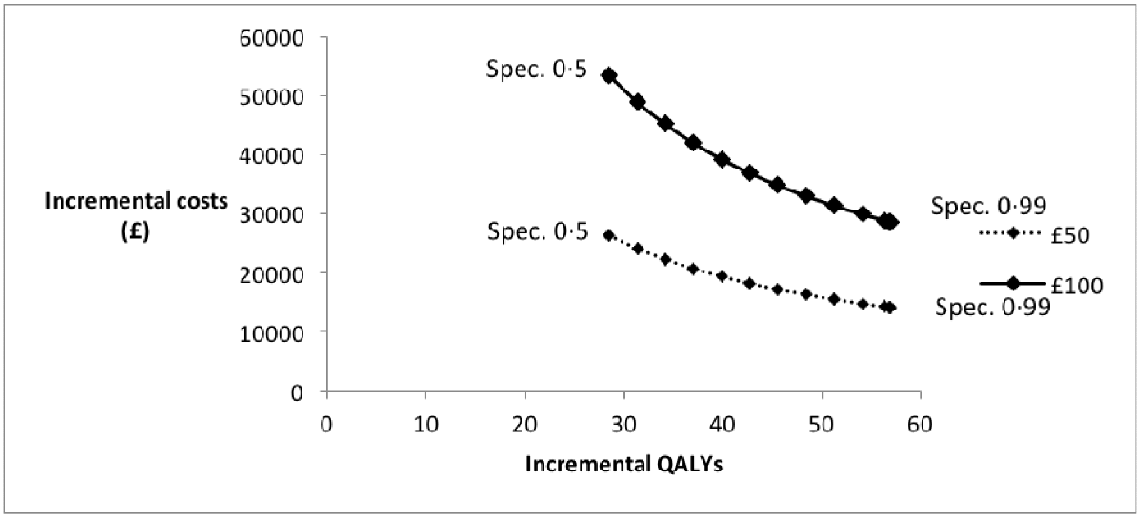

Scenario analysis

Table S3 – Scenario analysis: Mean survival estimates

	Scenario 1			Scenario 2		
Serum spectroscopy test cost (£)	ΔQALY	ΔCost	ICER	ΔQALY	ΔCost	ICER
50	12·83	-212808	-16582 (dominates)	77	540273	7016
100	12·83	287192	22377	77	1040273	13509

Table S4 - Additional costs scenario analysis

	Scenario 1			Scenario 2		
Serum spectroscopy test cost (£)	ΔQALY	ΔCost	ICER	ΔQALY	ΔCost	ICER
50	8·81	-379962	-43128 (dominates)	52·86	566247	10712
100	8·81	120038	13625	52·86	1066247	20171

Table S5 - Alternative prevalence estimates (1% prevalence) in primary care setting (Scenario 1)

	Scenario 1		
Serum spectroscopy test cost (£)	ΔQALY	ΔCost	ICER
50	17·62	-413220	-23452 (dominates)
100	17·62	86780	4925

Table S6– Alternative proportion of test negative cases referred to imaging (Scenario 1 only)

		Scenario 1		
Serum spectroscopy test cost (£)	% of test negative cases referred	ΔQALY	ΔCost	ICER
50	50 (base)	8·81	-422,116	-47,913
	75	17·62	-131,050	-7,438
	90	17·62	38,252	2,171
	100	17·62	151,120	8,577
100	50 (base)	8·81	77,884	8,840
	75	17·62	368,950	20,939
	90	17·62	538,252	30,548
	100	17·62	651,120	36,953

For peer review only

Web Extra Material

Appendix 1 – Clinical expert group

Clinical expert group participants: Mr Michael D Jenkinson (Walton Centre NHS Trust & University of Liverpool), Dr Paul Brennan (University of Edinburgh), Prof. Anthony Chalmers (University of Glasgow & Beatson Centre), Prof. Willie Hamilton (University of Exeter Medical School), Mr Kevin O'Neill (Imperial College Healthcare NHS Trust), Dr Allan Janes (Beatson West of Scotland Cancer Centre), Dr Babar Vaqas (Imperial College Healthcare NHS Trust), Dr David Kernick (St Thomas Health Centre, Exeter), Dr James Livermore (University of Oxford), Dr Ruth Board (Lancashire Teaching Hospitals NHS Trust), Prof. Timothy Dawson (Lancashire Teaching Hospitals NHS Trust), Miss Elvira Lekka (Lancashire Teaching Hospitals NHS Trust), Mr John Goodden (Leeds Teaching Hospital NHS Trust) and Dr. Samantha Mills (Walton Centre NHS Trust)

For peer review only

Appendix 2 – Decision tree model

The decision node probabilities that populate the decision tree model are reported in Table S1. The model is simplified by assuming that the reference test (MRI/CT) has perfect accuracy (100% sensitivity and 100% specificity). This is desirable if we wish to evaluate the test against an MRI/CT only diagnostic pathway in which these imaging studies are considered as the 'gold standard'. It is also difficult to obtain valid estimates of MRI/CT sensitivity and specificity for this indication as these are not reported in a comparable manner in the literature. This simplification will not substantially alter the economic results compared to using more realistic values for the scenarios under consideration. This is because it is assumed confirmation of the diagnosis always requires imaging and therefore outcomes for patients that would hypothetically be classified correctly using this test but incorrectly by MRI/CT will remain the same whether or not the test is used. The effect of less than perfect accuracy of the reference test is therefore the same as a (small) decrease in prevalence as a proportion of patients with the disease cannot benefit from the additional test due to incorrect subsequent testing. Outcomes are calculated by 'rolling-back' the tree for both cancer cases and non-cases and taking an average of the two groups weighted by the prevalence of brain tumours in the scenario population.

Table S1. Decision node probabilities for decision tree model describing the integration of a serum spectroscopy test in the current diagnostic pathway

**Cancer cases:**	**Probability to upper branch**	**Probability to lower branch**
Serum Spectroscopy Test (test sensitivity)	0·928	0·072
Standard MRI/CT (reference test sensitivity)	1	0
Fast-track MRI/CT (reference test sensitivity)	1	0
Referral decision	S1:0·5, S2: 1	S1:0·5, S2: 0
Non-cases:		
Serum Spectroscopy Test (test specificity)	0·085	0·915
Standard MRI/CT (reference test specificity)	0	1
Fast-track MRI/CT (reference test specificity)	0	1
Referral decision<br>S1 & S2: Scenarios 1 &2	S1:0·5, S2: 1	S1:0·5, S2: 0

For peer review only

Appendix 3 – Assumptions and details of cost effective analysis model

Effect of testing on time-to-diagnosis and time-to-treatment

To estimate the expected time-to-diagnosis under a fast-track pathway it is assumed that this would match the current median time-to-diagnosis for patients presenting with brain tumour in emergency care as observed in Aggarwal et al [5]. This study reported a cohort of high grade glioma patients from a single hospital in London, UK. No other suitable estimates of time-to-treatment or time-to-diagnosis were identified in the literature. This is the most common primary brain tumour in the indications considered in this evaluation. Median time-to-diagnosis for the standard pathway is also taken from the same source.

Effect of testing on use of imaging studies

The effect of spectroscopic testing on imaging study decisions of clinicians and patients is uncertain. Based on clinical expert input it was assumed, in the base case, that in secondary care all patients would continue to imaging, while in primary care 50% would continue to imaging following a negative spectroscopy result.

Effect of early diagnosis on patient outcomes

A systematic review of the literature failed to identify any studies that directly estimated the effect of earlier (or later) diagnosis on outcomes for primary brain tumours. Instead, based clinical expert guidance, estimates of the effects of time-to-treatment were sourced from the literature. The estimated effects are based on fitting natural history models to observational datasets of high grade glioma patients.[27,28] It is necessary to use a natural history model to produce survival estimates rather than using the survival data stratified by time-to-treatment directly, in order to adjust for potential confounding factors influencing both time-to-treatment and survival.

A one-to-one correspondence is assumed for the effects of an additional day added to the time-to-diagnosis and an additional day added to the time-to-treatment. Median survival by weeks between diagnosis and treatment are displayed in Table 2. The hazard ratio per additional day delay calculated in Do et al of 1·015 was applied to each day between diagnosis and treatment up to 28 days to calculate median survival time. Time between diagnosis and treatment beyond 28 days were fixed at the same median survival as 28 days.

Table S2. Relationship between time-to-diagnosis and survival

**Time-to-diagnosis (weeks)**	**Median survival (weeks)**
0	46
1	41·4
2	37·3
3	33·6
4	30·3

Source: Do et al 2000

Resource use and cost

It is assumed that there is no additional cost for converting a small proportion of standard referral to additional urgent referrals. This could be justified on the basis that patients receiving negative results allows more flexibility in the scheduling for these patients. This could create more opportunity to schedule patients with positive spectroscopy results sooner. As a larger proportion of patients are referred for immediate imaging it was assumed costs would increase because achieving this would require additional capacity. It was assumed that unit costs for MRI and CT grow exponentially with increasing proportion of immediate referrals such that costs double if 50% of patients are referred for immediate imaging.

Treatment costs are assumed to be the same for both fast-track and standard referrals that are diagnosed as cases. This may be a conservative assumption as treatment costs are likely to increase as disease progresses and therefore earlier treatment may, on average, be less costly than later treatment. However, it is also possible that

For peer review only

treatment costs could be increased if some cases are converted from palliative care to more aggressive treatment. The balance of these effects is unknown given the lack of appropriate data to estimate the effects. Secondary treatment and end-of-life costs are similarly assumed to be equal between fast track and standard referrals. This reflects the model assumption that all patients ultimately progress.

The time intervals considered (0-4 weeks) are too short for discounting to have an important impact on cost estimates therefore the effects of moving forward treatment on present value is not considered.

For peer review only

Appendix 4 - Total demand for serum spectroscopy tests in UK in two clinical scenarios

1. Primary care

An assumption that patients currently referred in direct-access imaging service would be selected for spectroscopic testing can provide a conservative estimate of the total number of patients likely to be tested. Three sources from the literature were considered:

1. A direct-access brain MRI service in Nottingham (UK) reported 130 patients per quarter (520 per year) among a population of 342,000.[32,33] The population of the UK is approximately 61 million therefore, assuming Nottingham is representative of the UK general population demand for brain MRI, there would be approximately 93,000 per year in the UK.
2. A direct access CT service for chronic headache in Glasgow (UK) reported 4404 patients referred over 8 years (551 per year) among a population of approximately 1 million.[31] Assuming this was representative of the UK generally then the demand for this type of service would be approximately 34,000 for the UK.
3. A direct access CT neuroimaging service in Lothian (UK) reported 389 exams per year for a population of approximately 442,000.[34] Assuming this was representative of the UK generally then the demand for this type of service would be approximately 54,000 for the UK.

The Glasgow estimate is particularly low and may result from limited uptake in the early part of the 8 year period for which data were reported. As it was not possible to determine if uptake increased over the period or was stable this estimate was not considered reliable. The Nottingham and Lothian estimates report potentially more stable and established services and are therefore more likely to reliably estimate demand. The likely demand for this novel test, if the indication is considered identical to neuroimaging, is therefore between 54,000 and 93,000. This is likely to be conservative as the test may well have a slightly broader indication than current neuroimaging. A reasonable point estimate may be approximately 75,000.

2. Secondary Care

An estimate of the total demand for serum spectroscopic tests in the UK can be made based on the reported incidence of brain tumour in the UK and the proportion of cases currently diagnosed through secondary (non-emergency) care. Annual incidence of malignant brain tumours in the UK is approximately 4,700.[35] Approximately one third of these are likely to present through secondary, non-emergency care. Using the positive predictive value for patients currently referred to neuroimaging in this setting of 3% suggests that for each case there will be 33 non-case patients receiving neuroimaging. In total, in the UK, this would imply 53,000 patients per year in secondary care may be suitable for spectroscopic testing.

For peer review only

Appendix 5 - Sensitivity Analysis

One-way sensitivity analysis (OWSA)

OWSA results for a range of test sensitivity are displayed in S1 and S2 as a series of points on a cost-effectiveness (CE) plane with incremental QALYs (intervention-control) on the x-axis and incremental costs (intervention - control) on the y-axis. Corresponding OWSA results for a range of test specificities in primary and secondary care are shown in Figure 3 and 4 in the main article text respectively. These are displayed with the ICER on the y-axis and the test specificity on the x-axis. Note that the estimated QALYs do not change with specificity in the model therefore changes in the ICER are due solely to changes in incremental costs.**Error! Reference source not found.** All OWSA analyses show results assuming the spectroscopy based test costs of £50 and £100.

Figure S1 – Results on CE plane over a range of test sensitivities (0·5-0·99) – Primary care

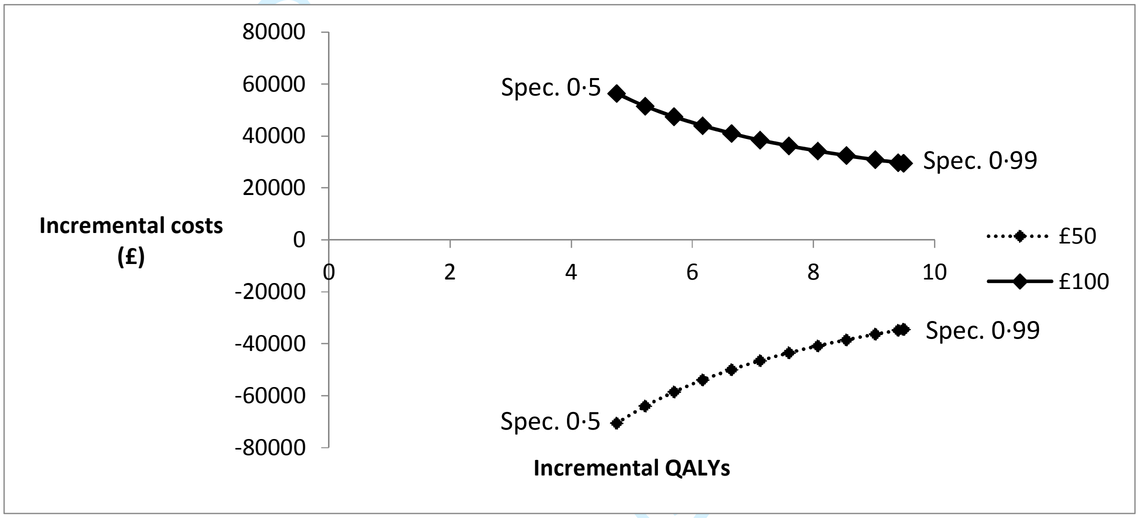

Figure S2 - Results on CE plane over a range of test sensitivities (0·5-0·99) – Secondary care

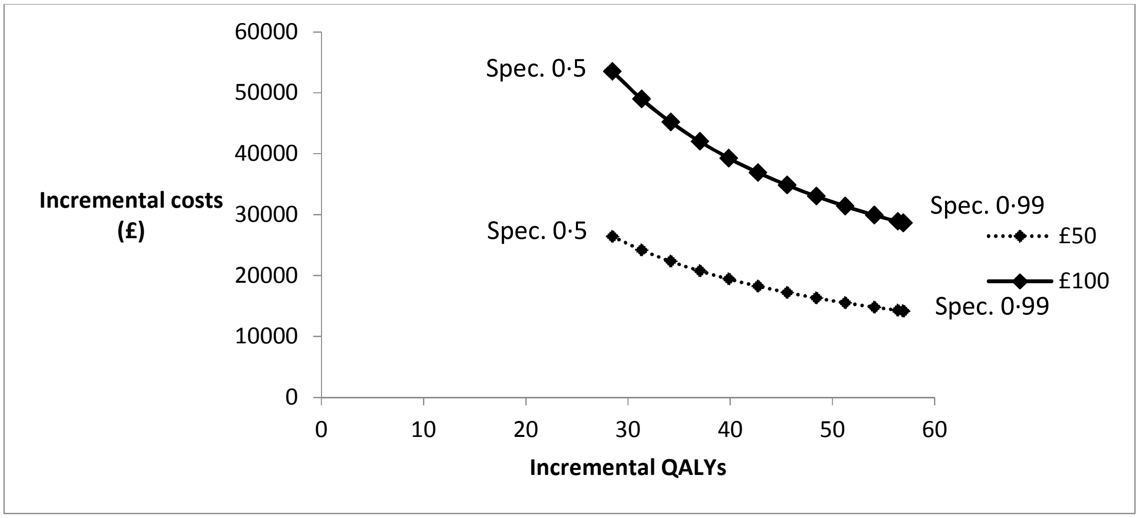

Scenario analysis

Table S3 – Scenario analysis: Mean survival estimates

	Scenario 1			Scenario 2		
Serum spectroscopy test cost (£)	ΔQALY	ΔCost	ICER	ΔQALY	ΔCost	ICER
50	12·83	-212808	-16582 (dominates)	77	540273	7016
100	12·83	287192	22377	77	1040273	13509

Table S4 - Additional costs scenario analysis

	Scenario 1			Scenario 2		
Serum spectroscopy test cost (£)	ΔQALY	ΔCost	ICER	ΔQALY	ΔCost	ICER
50	8·81	-379962	-43128 (dominates)	52·86	566247	10712
100	8·81	120038	13625	52·86	1066247	20171

Table S5 - Alternative prevalence estimates (1% prevalence) in primary care setting (Scenario 1)

	Scenario 1		
Serum spectroscopy test cost (£)	ΔQALY	ΔCost	ICER
50	17·62	-413220	-23452 (dominates)
100	17·62	86780	4925

Table S6– Alternative proportion of test negative cases referred to imaging (Scenario 1 only)

		Scenario 1		
Serum spectroscopy test cost (£)	% of test negative cases referred	ΔQALY	ΔCost	ICER
50	50 (base)	8·81	-422,116	-47,913
	75	17·62	-131,050	-7,438
	90	17·62	38,252	2,171
	100	17·62	151,120	8,577
100	50 (base)	8·81	77,884	8,840
	75	17·62	368,950	20,939
	90	17·62	538,252	30,548
	100	17·62	651,120	36,953

For peer review only

CHEERS Checklist
Items to include when reporting economic evaluations of health interventions

The **ISPOR CHEERS Task Force Report**, *Consolidated Health Economic Evaluation Reporting Standards (CHEERS)—Explanation and Elaboration: A Report of the ISPOR Health Economic Evaluations Publication Guidelines Good Reporting Practices Task Force*, provides examples and further discussion of the 24-item CHEERS Checklist and the CHEERS Statement. It may be accessed via the *Value in Health* or via the ISPOR Health Economic Evaluation Publication Guidelines – CHEERS: Good Reporting Practices webpage: http://www.ispor.org/TaskForces/EconomicPubGuidelines.asp

Section/item	Item No	Recommendation	Reported on page No/ line No
**Title and abstract**			
Title	1	Identify the study as an economic evaluation or use more specific terms such as "cost-effectiveness analysis", and describe the interventions compared.	1 / 1
Abstract	2	Provide a structured summary of objectives, perspective, setting, methods (including study design and inputs), results (including base case and uncertainty analyses), and conclusions.	3 / 1-29
**Introduction**			
Background and objectives	3	Provide an explicit statement of the broader context for the study.	5/2-12
		Present the study question and its relevance for health policy or practice decisions.	8/17-29
**Methods**			
Target population and subgroups	4	Describe characteristics of the base case population and subgroups analysed, including why they were chosen.	14/ 11-37
Setting and location	5	State relevant aspects of the system(s) in which the decision(s) need(s) to be made.	14/11-37
Study perspective	6	Describe the perspective of the study and relate this to the costs being evaluated.	26/3-32
Comparators	7	Describe the interventions or strategies being compared and state why they were chosen.	11-12/33-2
Time horizon	8	State the time horizon(s) over which costs and consequences are being evaluated and say why appropriate.	12/14-22
Discount rate	9	Report the choice of discount rate(s) used for costs and outcomes and say why appropriate.	12/2-3
Choice of health outcomes	10	Describe what outcomes were used as the measure(s) of benefit in the evaluation and their relevance for the type of analysis performed.	11/26-32
Measurement of effectiveness	11a	*Single study-based estimates:* Describe fully the design features of the single effectiveness study and why the single study was a sufficient source of clinical effectiveness data.	11/9-22

For peer review only

	11b	*Synthesis-based estimates:* Describe fully the methods used for identification of included studies and synthesis of clinical effectiveness data.	
Measurement and valuation of preference based outcomes	12	If applicable, describe the population and methods used to elicit preferences for outcomes.	9/1
Estimating resources and costs	13a	*Single study-based economic evaluation:* Describe approaches used to estimate resource use associated with the alternative interventions. Describe primary or secondary research methods for valuing each resource item in terms of its unit cost. Describe any adjustments made to approximate to opportunity costs.	11/33-40
	13b	*Model-based economic evaluation:* Describe approaches and data sources used to estimate resource use associated with model health states. Describe primary or secondary research methods for valuing each resource item in terms of its unit cost. Describe any adjustments made to approximate to opportunity costs.	12-13/4-4<br>24/1-17
Currency, price date, and conversion	14	Report the dates of the estimated resource quantities and unit costs. Describe methods for adjusting estimated unit costs to the year of reported costs if necessary. Describe methods for converting costs into a common currency base and the exchange rate.	11/38-40
Choice of model	15	Describe and give reasons for the specific type of decision-analytical model used. Providing a figure to show model structure is strongly recommended.	9/1
Assumptions	16	Describe all structural or other assumptions underpinning the decision-analytical model.	23-24/1-6
Analytical methods	17	Describe all analytical methods supporting the evaluation. This could include methods for dealing with skewed, missing, or censored data; extrapolation methods; methods for pooling data; approaches to validate or make adjustments (such as half cycle corrections) to a model; and methods for handling population heterogeneity and uncertainty.	12-13/4-4
**Results**			15/3-20
Study parameters	18	Report the values, ranges, references, and, if used, probability distributions for all parameters. Report reasons or sources for distributions used to represent uncertainty where appropriate. Providing a table to show the input values is strongly recommended.	
Incremental costs and outcomes	19	For each intervention, report mean values for the main categories of estimated costs and outcomes of interest, as well as mean differences between the comparator groups. If applicable, report incremental cost-effectiveness ratios.	15/3-20
Characterising uncertainty	20a	*Single study-based economic evaluation:* Describe the effects of sampling uncertainty for the estimated incremental cost and incremental effectiveness parameters, together with the impact	

For peer review only

		of methodological assumptions (such as discount rate, study perspective).	
	20b	*Model-based economic evaluation:* Describe the effects on the results of uncertainty for all input parameters, and uncertainty related to the structure of the model and assumptions.	17-18/2-2
Characterising heterogeneity	21	If applicable, report differences in costs, outcomes, or cost-effectiveness that can be explained by variations between subgroups of patients with different baseline characteristics or other observed variability in effects that are not reducible by more information.	
**Discussion**			
Study findings, limitations, generalisability, and current knowledge	22	Summarise key study findings and describe how they support the conclusions reached. Discuss limitations and the generalisability of the findings and how the findings fit with current knowledge.	19-20/4-20
**Other**			
Source of funding	23	Describe how the study was funded and the role of the funder in the identification, design, conduct, and reporting of the analysis. Describe other non-monetary sources of support.	2/28-33
Conflicts of interest	24	Describe any potential for conflict of interest of study contributors in accordance with journal policy. In the absence of a journal policy, we recommend authors comply with International Committee of Medical Journal Editors recommendations.	2/23-26

For consistency, the CHEERS Statement checklist format is based on the format of the CONSORT statement checklist

The **ISPOR CHEERS Task Force Report** provides examples and further discussion of the 24-item CHEERS Checklist and the CHEERS Statement. It may be accessed via the *Value in Health* link or via the ISPOR Health Economic Evaluation Publication Guidelines – CHEERS: Good Reporting Practices webpage: http://www.ispor.org/TaskForces/EconomicPubGuidelines.asp

The citation for the CHEERS Task Force Report is:
Husereau D, Drummond M, Petrou S, et al. Consolidated health economic evaluation reporting standards (CHEERS)—Explanation and elaboration: A report of the ISPOR health economic evaluations publication guidelines good reporting practices task force. Value Health 2013;16:231-50.

For peer review only

BMJ Open

Health economic evaluation of a serum based blood test for brain tumour diagnosis: Exploration of two clinical scenarios.

Journal:	*BMJ Open*
Manuscript ID	bmjopen-2017-017593.R1
Article Type:	Research
Date Submitted by the Author:	19-Dec-2017
Complete List of Authors:	Gray, Ewan; Health Improvement Scotland<br>Butler, Holly; Pure and Applied Chemistry; Technology & Innovation Building<br>Board, Ruth; Lancashire Teaching Hospitals NHS Foundation Trust, Rosemere Cancer Centre<br>Brennan, Paul; Western General Hospital, Department of Clinical Neurosciences<br>Chalmers, Anthony; University of Glasgow, Institute of Cancer Sciences; Beatson West of Scotland Cancer Centre<br>Dawson, Timothy; Lancashire Teaching Hospitals NHS Foundation Trust, Neurosurgery Department<br>Gooden, John; Leeds General Infirmary, Neurosurgery Department<br>Hamilton, Willie; University of Exeter Medical School, Primary Care Diagnostics<br>Hegarty, Mark; University of Strathclyde, Pure & Applied Chemistry<br>James, Allan; University of Glasgow Institute Of Molecular Cell And Systems Biology<br>Jenkinson, Michael; The Walton Centre NHS Foundation Trust<br>Kernick, David; St Thomas' Medical Group<br>Lekka, Elvira; Lancashire Teaching Hospitals NHS Foundation Trust, Neurosurgery Department<br>Livermore, Laurent; Oxford University Hospitals, NHS Foundation Trust, Department of Neurosurgery<br>Mills, Samantha; The Walton Centre NHS Foundation Trust<br>O'Neill , Kevin; Imperial College London<br>Palmer, David; University of Strathclyde, Pure & Applied Chemistry<br>Vaqas, Babar; Imperial College London, John Fulcher Neuro-Oncology Laboratory<br>Baker, Matthew; University of Strathclyde
**Primary Subject Heading**:	Health economics
Secondary Subject Heading:	Diagnostics, Health economics, Oncology
Keywords:	Biophysics < BASIC SCIENCES, HEALTH ECONOMICS, Neurology < INTERNAL MEDICINE, Neurological oncology < NEUROLOGY, Adult oncology < ONCOLOGY, Head & neck imaging < RADIOLOGY & IMAGING

For peer review only

Health economic evaluation of a serum based blood test for brain tumour diagnosis: Exploration of two clinical scenarios.

Ewan Gray[1†], Holly J. Butler[2,3†], Ruth Board[4], Paul M. Brennan[5], Anthony J Chalmers[6,7], Timothy Dawson[8], John Goodden[9], Willie Hamilton[10], Mark G. Hegarty[2,3], Allan James[11], Michael D. Jenkinson[12, 13], David Kernick[14], Elvira Lekka[8], Laurent J. Livermore[15], Samantha J. Mills[12], Kevin O'Neill[16], David S. Palmer[3,17], Babar Vaqas[16] Matthew J. Baker[2,3*]

[1] Health Improvement Scotland, NHS Scotland, Delta House, 50 West Nile Street, Glasgow, G1 2NP, UK; [2] WestCHEM, Department of Pure and Applied Chemistry, University of Strathclyde, Technology and Innovation Centre, 99 George Street, Glasgow, G1 1RD, UK; [3] ClinSpec Diagnostics Limited, Technology and Innovation Centre, 99 George St, G1 1RD, UK; [4] Rosemere Cancer Centre, Lancashire Teaching Hospitals NHS Trust, Royal Preston Hospital, Sharoe Green Lane, Preston, PR2 9HT, UK; [5] Department of Clinical Neurosciences, Western General Hospital, Edinburgh, EH4 2XU, UK; [6] Beatson West of Scotland Cancer Centre, 1053 Great Western Road, Glasgow, G12 0YN, UK; [7] Institute of Cancer Sciences, Wolfson Wohl Cancer Research Centre, University of Glasgow, Glasgow, G61 1QH; [8] Neurosurgery Department, Lancashire Teaching Hospitals NHS Trust, Royal Preston Hospital, Sharoe Green Lane, Preston, Lancashire, PR2 9HT, UK; [9] Neurosurgery Department, Leeds General Infirmary, Leeds, LS1 3EX, UK; [10] Primary Care Diagnostics, University of Exeter Medical School, College House, University of Exeter, Exeter, Devon, EX1 2LU, UK; [11] Institute of Molecular Cell and Systems Biology, Bower Building, Glasgow, G12 8QQ, UK; [12] The Walton Centre NHS Foundation Trust, Lower Lane, Fazakerley, Liverpool, L9 7LJ, UK; [13] Institute of Translational Medicine, Clinical Science Centre, University of Liverpool, Liverpool, L9 7LJ; [14] St Thomas' Medical Group, Cowick Street, Exeter, EX4 1HJ, UK; [15] Department of Neurosurgery, Oxford University Hospitals, NHS Foundation Trust, John Radcliffe Hospital, Oxford, OX3 9DU, UK; [16] John Fulcher Neuro-Oncology Laboratory, Imperial College, London, SW7 2AZ, UK; [17] WestCHEM, Department of Pure and Applied Chemistry, University of Strathclyde, Thomas Graham Building, 295 Cathedral Street, Glasgow, G1 1XL, UK

*Corresponding author: Dr Matthew J Baker, matthew.baker@strath.ac.uk, +44(0)141 548 4700, @ChemistryBaker

†These authors contributed equally to the paper

For peer review only

Copyright/Licence for publication

The Corresponding Author has the right to grant on behalf of all authors and does grant on behalf of all *authors,* a worldwide licence *to the Publishers and its licensees in perpetuity, in all forms, formats and media (whether known now or* created in the future), to i) publish, reproduce, distribute, display and store the Contribution, ii) translate the Contribution into other languages, create adaptations, reprints, include within collections and create summaries, extracts and/or, abstracts of the Contribution, iii) create any other derivative work(s) based on the Contribution, iv) to exploit all subsidiary rights in the Contribution, v) the inclusion of electronic links from the Contribution to third party material where-ever it may be located; and, vi) licence any third party to do any or all of the above

Authors' Contributions

EG conducted the health economic assessment including data analysis, data interpretation, and writing. HJB contributed to data interpretation, writing and providing details regarding clinical spectroscopy. MJB is the principal investigator of this research, and initiated this interdisciplinary project and team. RB, PMB, AJC, TD, JG, WH, AJ, MDJ, DK, EL, LJL, SJM, KO, and BV are members of the clinical focus group involved in spearheading translation; all of whom provided feedback on the manuscript. MGH, DSP, HJB and MJB are involved in the ClinSpec Diagnostics Limited project.

Competing Interest Declaration

MJB, MGH, HJB and DSP are all involved in ClinSpec Diagnostics Limited a prospective spin out company from the University of Strathclyde focusing on the translation of serum spectroscopic diagnostics (Company Number SC535447)

Role of the Funding Source

Scottish Enterprise is Scotland's main economic development agency and non-departmental public body of the Scottish Government. They work with partners in both the private and public sectors to exploit the best opportunities to deliver a significant, lasting effect on the Scottish economy. The High-Growth Spinout Programme helps researchers take their ideas and inventions from the lab to the global marketplace.

Acknowledgements

The authors acknowledge the support from Rosemere Cancer Foundation, Brain Tumour North West, EPSRC, and the Sydney Driscoll Neuroscience Foundation for funding.

For peer review only

Data Sharing Statement

No additional data available

For peer review only

Abstract

Objectives

To determine the costs and health benefits of a serum-based spectroscopic triage tool for brain tumours, that could potentially reduce diagnostic delays in the current clinical pathway.

Design

A model-based health economic assessment. Decision tree models were constructed based on simplified diagnostic pathways. Models were populated with parameters identified from rapid reviews of the literature and clinical expert opinion.

Setting

Explored as a test in both primary and secondary care (neuroimaging) in the UK health service, as well as application to the US.

Participants

Calculations based upon an initial cohort of 10,000 patients. In primary care, it is estimated that the volume of tests would approach 75,000 per annum. The volume of tests in secondary care is estimated at 53,000 per annum.

Main Outcome Measures

The primary outcome measure was quality adjusted life years (QALYs), which were employed to derive incremental cost-effectiveness ratios (ICERs) in a cost-effectiveness analysis.

Results

Results indicate that using a blood-based spectroscopic test in both scenarios may be considered highly cost-effective in a health technology assessment (HTA) agency decision making process, as ICERs were well below standard threshold values of £20,000 to £30,000 per QALY. This test may be cost-effective in both scenarios with test sensitivities and specificities as low as 80%; however, the price of the test would need to be lower (less than approximately £40).

Conclusion

Use of this test as triage tool in primary care has the potential to be both more effective and cost saving for the health service. In secondary care, this test would also be deemed more effective than the current diagnostic pathway.

For peer review only

Strengths and Limitations of this Study

- Simplified models of clinical pathways were mapped with input from, and consensus among, a wide range of clinical experts including neurosurgeons, neurooncologists, neuropathologists, neuroradiologists, and primary care experts.
- The spectroscopic blood test was highly sensitive and specific in retrospective data, with performances of 91.5 and 83.0% respectively. There is potential for this to contribute towards improved prognosis for patients, as well as healthcare savings.
- This study is based upon proof-of-concept studies, in advance of a pending prospective clinical trial. As these samples are retrospective, there is the possibility the diagnostic performance will not be as high in prospective studies.
- A lack of clinical trial evidence necessitates the estimate of long-term benefits of improved diagnostic protocols based on disease natural history models. This creates additional uncertainties.
- The precise patient population for whom the test may be suitable in the primary care setting is difficult to establish at this stage in development. This study considers a limited definition of eligibility that may need revision in light of future evidence.

For peer review only

Introduction

At an average of 20 years, patients with malignant brain tumours have the highest number of years of life lost, compared to all other primary cancers.[1] This, at least in part, may relate to diagnostic delays, reflecting the non-specific early symptoms, such as headache and dizziness, from which general practitioners (GPs) must identify patients at risk for further investigation. The lack of a low-cost diagnostic and/or screening tools available within the health service contributes to this delay. We have recently demonstrated that a spectroscopic test using blood-serum is able to effectively identify brain tumours in patients with sensitivities and specificities as high as 91·5% and 83% respectively.[2,3] This approach is based upon Fourier-transform infrared (FTIR) spectroscopy and can detect disease-specific signatures, which are extracted mathematically using pattern recognition and machine learning algorithms.

Current Diagnostic Pathway

Currently, patients who are symptomatic with a brain tumour visit their GP on average five times before being referred to secondary care.[4] Partly as a result of this diagnostic delay up to 61% of brain tumour diagnoses occur in an emergency setting, often following a seizure.[5] Patients diagnosed by the emergency route have a poorer prognosis.[6,7] For some patients this may be because the disease is at a more advanced stage at diagnosis. The complications precipitating the emergency admission make an additional contribution to mortality.

Screening programmes for breast, prostate, cervical, and colorectal cancer have proved effective for diagnosing patients at an earlier stage, which can result in a better prognosis.[8-11] These screening programmes have had a significant impact in reducing the number of patients presenting as an emergency. To date there has been no accessible and economically viable diagnostic tool for early detection of asymptomatic and symptomatic brain tumours.[12] The addition of a rapid and accurate blood test for patients with suspected primary brain tumours (symptomatic patients) therefore has the potential to improve outcomes by allowing prioritisation of patients most at risk of a brain tumour for further investigation. Under the current patient pathway it is not feasible to provide fast-track diagnostic imaging because the number of patients with non-specific headache symptoms is very large and the positive predictive value (PPV) on the basis of symptoms alone is less than 3% for all symptoms other than a new-onset seizure.[13,14]

Magnetic resonance imaging (MRI) and computed tomography (CT) are the current gold standard for identifying structural brain lesions including tumours. Treatment decisions made at the neuro-oncology multidisciplinary team (MDT) meeting are often based on the imaging alone. Following surgical resection or biopsy with histopathology and molecular analysis, definitive treatment can be planned.[15] Surgery to secure the tissue diagnosis has a small risk to the patient of neurological deterioration and death.[16]

The diagnostic pathway also represents a significant cost burden to the health service, with a single MRI and CT scan in the UK costing around £164 and £85, respectively (National

For peer review only

Schedule of Reference Costs [2014-15], Medicare Physician Fee Schedule 2016). A typical timeframe of the diagnostic pathway for brain tumours, specifically primary gliomas, is illustrated in Figure 1, and effectively highlights the significant wait that a symptomatic patient may have before receiving brain imaging. Even from this stage, regardless of the time to GP referral or emergency presentation, full diagnosis may take a further 5 weeks.

Serum Spectroscopic Diagnostics

This novel blood test for early brain tumour detection is based upon the interaction of infrared (IR) light with biological components of blood serum (**Figure 2**). Specifically, using attenuated total reflectance (ATR)-FTIR spectroscopy, specific bond vibrations of given molecules can be elucidated from serum samples, thus providing a unique insight into the composition via an absorbance spectrum.[17] Benefits of an ATR-FTIR based approach include a robust, user-friendly methodology without extensive sample preparation, that would readily fit into a clinical setting.[18] In short, serum is obtained according to standard protocols and can be snap frozen and stored at -80° until the point of analysis. A small volume of serum is required for analysis (1-5 μL), which is pipetted onto a crystal, known as an internal reflection element (IRE), where IR non-destructively interacts with the sample and produces an IR spectrum, with peaks representative of known bond vibrations and hence biomolecular constituents.

Blood serum is a complex medium that contains a variety of biomolecules, including around 20,000 proteins, that may be employed as diagnostic biomarkers.[19] In the case of brain tumours, such blood based technologies are limited, due to a lack of an established brain tumour specific diagnostic biomarker.[20] With our spectroscopic approach, rather than derive single biomarker specific information, a global signature is obtained, which encompasses the entire biomolecular makeup. This is epitomised as an equally complex biological absorbance spectrum, which contains a wealth of diagnostic information (an example may be seen in **Figure 3**).

Pattern recognition and machine learning algorithms using spectral features from FTIR data have been demonstrated as rapid and accurate for separating primary brain cancer and non-cancer cases.[2,21] When these algorithms are applied to this information rich dataset, the relationship between all biological components of the sample is ascertained, providing a multi-dimensional analysis of the sample.

This approach has been able to not only detect between cancer and non-cancer, but also stratify based upon cancer pathology.[2] For further information and in-depth description of the methodology, we direct the readers to the following fundamental review and recent research papers.[2,3,17,21] The ability to triage patients likely to have a brain tumour based on serum sample alone raises the possibility of systematic triaging prior to investigation with more expensive (MRI/CT imaging) and invasive (biopsy) tests. One major impact of having a serum test available would be a clear reduction in the number of unnecessary brain scans; however, as this test is also able to differentiate between primary and secondary tumours, there could also be a knock-on reduction of chest and abdomen scans which are conducted to

For peer review only

rule out primary disease elsewhere. There is also the possibility that this approach will reduce the incidence of incidental abnormalities which in themselves can cause considerable distress. Ultimately, it is expected that this could allow earlier and potentially more effective treatment of brain tumours.

Aims & Objectives

The aim of the economic evaluation is to assess the potential cost-effectiveness of this spectroscopic technology, in advance of any prospective study results being available. There are three main objectives for the evaluation. The first objective is to create a map of where the test could be used in the clinical pathway. The second is to assess the potential cost-effectiveness of the technology, if the performance shown in the case-control study is replicated prospectively. This will give an indication of whether the technology would meet the criteria for acceptance for use in the National Health Service that are applied by Health Technology Assessment agencies (HTA). Related to this, the third objective is to define the level of performance in prospective trials, and any additional evidence that would be needed to meet the cost-effectiveness criteria of an HTA decision-making process. This can include diagnostic performance and also effects on long-term outcomes such as survival and resource use. To achieve these objectives a simplified economic model of two important clinical scenarios is used to explore cost effectiveness.

For peer review only

Methodology

Mapping the Clinical Pathway

In order to appreciate the current clinical pathway and determine an appropriate entry point for a serum spectroscopic test, a pan-UK clinical focus group was established. This cohort included neurosurgeons, clinical and medical oncologists, neuropathologists, neuroradiologists, academic GPs with special interests, and experts in primary care diagnostics (see Appendix 1).

Cost Effectiveness Analysis

A cost-effectiveness analysis (CEA) was conducted to calculate the effects on health outcomes and health service costs of introducing spectroscopic testing in each of two scenarios. The health outcomes considered were life-years and quality-adjusted life-years (QALYs).

A decision tree was used to model the pathway for patients presenting with symptoms warranting a referral for MRI/CT imaging for suspected brain tumour (**Figure 4**). Separate models were considered for primary and secondary care. The time horizon of the model is 2 years and the perspective is that of the health care service. A two-year horizon was selected because of the short duration of survival in this patient group: median survival is approximately 1 year for high grade gliomas, which are the commonest malignant primary brain tumour. In all scenarios the comparator is the current diagnostic pathway (i.e. imaging alone). Further details regarding the node probabilities, simplifications and assumptions of the model can be found in **Appendix 2**.

Diagnostic Performance

The sensitivity and specificity of the test for detecting brain tumours has been demonstrated in a series of case-control studies using historical samples from the Brain Tumour North West and Walton Centre NHS bio-banks.[2,3] In the key case-control study nine FTIR spectra were collected from each of 433 patients.[3] Of these 134 were from patients with primary brain tumours (64 high grade glioma), 177 were from patients with cerebral metastases and 122 were from non-cancer controls. FTIR spectra were analysed using the random forest method to fit a classification model. Classification performance was estimated by applying the fitted model to a test set containing 20% of the patients from the original data set that were not used in the model fitting step (hold-out test set). Classification statistics are computed as averages of this process, iterated 96 times using random training and test sets. Under the best available model, sensitivity estimated by this method was 92·8% and specificity was 91·5% for the analysis of cancerous vs. non-cancerous serum.[3] These classification statistics were established using 96 independent iterations of a Random Forest model, and resulted in standard deviations of 1.1% and 1.9% respectively.

Establishing whether the performance demonstrated case-control data from historical samples translates to equivalent performance when applied prospectively in clinical practice is the

For peer review only

subject of a planned clinical trial. This is critical to demonstrating the clinical and cost-effectiveness of a serum spectroscopy test as part of the diagnostic pathway for brain tumours.

Prevalence of disease

Prevalence data were sourced from the literature based on clinical expert guidance. Brain tumour prevalence in scenario 2 (secondary care) was assumed to be 3% based on observed rates of primary brain tumour diagnosis among patients referred for brain imaging for suspected cancer in secondary care.[22-24] In scenario 1 (primary care) an estimated prevalence of 0·5% is used based on case-control evidence and expert opinion of the prevalence among patients who would be considered for direct access imaging, using MRI or CT, where this is available and referral to neurology where it is not.[25,26] An alternative prevalence of 1% was explored for scenario 1 based on unpublished data from a direct access imaging service in the UK (P. Brennan, personal communication).

The effect of serum spectroscopy testing on the time-to-diagnosis and time-to-treatment is discussed in Appendix 3, additional to the effect of testing on the use of imaging studies, and also on the patient outcomes. The primary assumptions in this model are that first, the expected time-to-diagnosis would match the current median time-to-diagnosis for patients presenting with brain tumour in emergency care as observed in Aggarwal et al.[5] Furthermore, based upon expert opinion, it is assumed that in secondary care all patients would continue to imaging, while in primary care 50% would continue to imaging following a negative spectroscopy result. This is a conservative estimate associated with the possibility that an imaging test will still be required in some cases based on interpretation of a patient's symptoms and the other non-tumour diagnoses being considered by the clinician. Finally, the effects of early diagnosis on the outcome of brain tumours is estimated using literature describing fitting natural history models to observational datasets of high grade glioma patients.[27,28]

Utility Weights

Health state utility weights are applied to life-years to generate quality adjusted life years (QALYs). A systematic review of health state utility weights for high grade glioma, the most common and aggressive primary brain tumour, was conducted to identify suitable utility weights. Due to heterogeneity it was not considered suitable to pool the estimates. The most appropriate health state utility weight was taken from a previous UK economic evaluation of glioblastoma treatment.[29] A value of 0·89 was used in the base case.

Resource Use and Costs

Resource use includes the application of a spectroscopic serum test to all patients prior to imaging, the imaging studies used in the diagnostic process, outpatient neurology clinic visits and general practitioner visits. In the UK analysis, unit costs for imaging studies are taken from UK NHS reference costs (2014/15), clinic and GP visits from the PSSRU costs schedule (Table 1). In the USA analysis, unit costs are taken from Medicare reimbursement schedules. Unit costs of the test were applied at an upper bound and lower bound rather than a single value as these products have not yet been commercialized. Bounds were set by consultation

For peer review only

with scientists developing the tests. Additional resource use and cost assumptions are described in Appendix 3.

Table 1. Unit costs and comparison for the brain tumour diagnostic pathway

Item	UK Cost per unit (£) (2015 prices)	USA cost per unit ($) (2016 prices)	Source(s)
CT imaging study [CT head]	85	163	National Schedule of Reference Costs (2014 – 15), Medicare Physician Fee Schedule 2016
MRI imaging study [MRI brain with contrast]	164	380	National Schedule of Reference Costs (2014 – 15), Medicare Physician Fee Schedule 2016
Neurology outpatient appointment	35	76	PSSRU 2016, Medicare Physician Fee Schedule 2016
GP visit	47·25	76	PSSRU 2016, Medicare Physician Fee Schedule 2016
Stable disease monitoring costs	116 per 3 months	154	[29], exchange rate adjusted
Serum spectroscopy test	Lower limit: 50<br>Upper limit: 100	Lower limit: 100<br>Upper limit: 200	Assumed prices

Incremental cost effectiveness ratios (ICER)

The comparative cost-effectiveness of spectroscopic testing compared to no testing is summarized by the ICER defined as:

$$ICER = \frac{C_s - C_n}{H_s - H_n}$$

C_s and C_n are the total costs with spectroscopic testing and no testing respectively. Equivalently H_s and H_n are the total QALYs with and without spectroscopic testing. The ICER can be interpreted as the additional cost per QALY gained.

For peer review only

Base Case and Sensitivity Analysis

ICERs were calculated for scenarios 1 and 2 using the base case parameter estimates. Base case analysis was repeated for UK and USA settings. Additional sensitivity analyses are reported for the UK setting only.

Sensitivity analyses included one-way sensitivity analysis (OWSA), systematically varying a single parameter in the model, and scenario analysis in which specific model assumptions were altered. OWSA were conducted for test sensitivity, specificity and test cost. Scenario analyses included assuming an additional consultation cost for discussion of test results, assuming a higher proportion of patients continue to imaging following a negative spectroscopy result in primary care, and using mean survival rather than median survival.

A probabilistic sensitivity analysis (PSA) was conducted to explore the effects of joint uncertainty in the parameter estimates on the model results.[30]

For peer review only

Results

Mapping the clinical pathway

For the first study objective, initial discussion with clinical experts indicated that there are potential uses for the test in both primary and secondary care. The main advantage of employing a cost-effective spectroscopic blood test in the diagnostic pathway is to use it as a triage test. This prioritises more urgent cases for access to services, and acts as a gate-keeper (requiring a positive result in some conditions to give access to services) for imaging studies.

Two clinical scenarios are mapped out below and subsequently explored in this early economic evaluation of the serum spectroscopy test for aiding diagnosis of brain tumours:

1. Triage tool in primary care

The primary care scenario explores a population of patients with a clinical presentation that warrants further investigation of possible brain tumour. This would include some patients with headaches and some with focal neurological deficits. This is the group of patients who would be considered for direct access imaging, using MRI or CT, where this is available and referral to neurology where it is not.[31] The blood test is used to provide rapid information, within 24 hours, where a positive result would lead to patients receiving more timely access to imaging. It may also be the case that negative test results, in addition to establishing the low probability of a brain tumour, could also provide some reassurance for those patients that must wait for imaging. The total volume of tests would be approximately 75,000 per year in the UK (see Appendix 4 for further details).

2. Triage tool in secondary care

In this scenario the population is the group of patients that are currently referred for imaging studies from secondary care for suspected brain tumour, typically via neurology clinics. This is the patient group for whom the clinical presentation has the highest positive predictive value (PPV). However, even in this high risk group, the odds of a brain tumour being present are approximately 1:33.[22-24] Again, the spectroscopy test is used to provide rapid information to allow a subset of these patients to access immediate imaging and provide reassurance to other patients who may have to wait longer for definitive imaging studies and diagnosis. The extent of the benefits of triage in this scenario is likely to vary by locality depending on the capacity constraints on imaging and pathology services. This evaluation uses estimates of the delays in diagnosis, and potential improvements in the speed of diagnosis, from a consecutive patient case series in London, UK.[5] The total volume of tests if this scenario occurred would be approximately 53,000 per year in the UK (see Appendix 4 for further details).

For peer review only

Cost Effectiveness Assessment

The standard threshold value per QALY gained in the UK, is considered to be between £20,000 to £30,000. Below this value, a healthcare intervention may be considered cost effective, whereas a negative ICER value would be deemed cost saving. Base case results for primary care (scenario 1) and secondary care (scenario 2) are presented in Table 2. Note results are reported for cohorts of 10,000 patients.

Table 2. Incremental QALYs, costs and ICERs for Scenarios 1 and 2, UK and USA

	**Scenario 1 – Primary Care**			**Scenario 2 – Secondary Care**		
**Serum spectroscopy test cost (£)**	**ΔQALY**	**ΔCost**	**ICER**	**ΔQALY**	**ΔCost**	**ICER**
**UK**						
**50**	8·81	-422,116	-47,913 (dominates)	52·86	527,646	9,982
**100**	8·81	77,884	8,840	52·86	1,027,646	19,441
**USA**						
**100**	8·81	-1,718,475	-195,058 (dominates)	52·86	536,702	10,153
**200**	8·81	-218,475	-24,798 (dominates)	52·86	2,036,702	38,530

ΔQALY, ΔCost: difference is QALYs/Costs (with serum spectroscopy test – without test), 10K patients
ICER: Incremental cost-effectiveness ratio

Incremental cost-effectiveness ratios were well below standard threshold values of £20,000 to £30,000 per quality adjusted life year (QALY) gained used in the UK, and similar thresholds used internationally, provided the test cost did not exceed £100. The base case results demonstrate that the serum spectroscopy test dominates (more effective and less costly) standard care at the lower bound of test cost in the primary care setting in both the UK and USA. At the upper bound of test cost the ICERs may be within commonly used thresholds or, in the case of the USA, remain dominant to standard care. In the secondary care setting ICERs of £9,982 and $10,153 at the lower bounds of test cost indicate that this test is potentially cost-effective in this setting. At the upper bounds ICERs may still be within commonly used thresholds for cost-effectiveness.

Sensitivity Analysis Results

The performance of the test with regards to levels of sensitivity and specificity are addressed using sensitivity analysis. OWSA results for a range of test specificities are displayed in Figure 5 and 6, for primary and secondary care respectively, displaying the ICER with

For peer review only

varying test specificity. Note that the estimated QALYs do not change with specificity in the model therefore changes in the ICER are due solely to changes in incremental costs. Varying sensitivity changes both estimated QALYs and estimated costs therefore results of the OWSA for test sensitivity are presented on the cost-effectiveness plane (Appendix 5). In primary care, using the upper cost limit of £100 it is evident that the test is deemed cost effective at specificities of around 0·9 and above, where the ICER is below standard thresholds. In contrast, at the lower cost limit, the test is cost effective at specificities above 0·8. Although the serum spectroscopy test is not cost saving at low cost (or near perfect specificities), the test is still considered cost effective at specificity levels around 0·7 and 0·8 for £50 and £100 pricing respectively.

The OWSA results highlight how ICERs in scenario 2 (secondary care) are strongly influenced by test sensitivity while ICERs in scenario 1 (primary care) are more strongly influenced by test specificity. These features are a result of the varying prevalence of disease and the assumption in scenario 2 that 100% of patients are referred for imaging following negative result. It should be noted that test specificity is important in both scenarios. Relatively small improvements in test specificity can substantially change the ICER, while larger improvements in test sensitivity are required to substantially alter the ICERs.

Additional scenario analyses are also reported in Appendix 5. These demonstrate that results are robust to using mean survival estimates rather than median survival estimates and including additional consultation costs for positive test results. If the prevalence of brain tumours in Scenario 1 is 1% rather than 0·5% the incremental QALYs increase substantially and the ICERs are reduced.

The PSA results reported in Figure 7 and 8 indicate that at a test cost of £50 and an ICER threshold of £30,000 per QALY there is a near 100% probability the serum spectroscopy test is cost-effective in scenario 1 and approximately 90% probability it is cost-effective in scenario 2. The corresponding probabilities at the upper bound cost of £100 are approximately 85% and 75%.

For peer review only

Discussion

This economic evaluation establishes the potential for serum spectroscopy to have a role in the diagnosis of both benign and malignant brain tumours in both primary and secondary care. The potential costs and health benefits of testing using a spectroscopic method prior to CT/MRI tests (or in some scenarios to avoid imaging) have been estimated based on a mathematical model with parameter values taken from published studies and expert opinion. This diagnostic tool is sensitive to all brain tumours (benign or malignant), however, this assessment is closely aligned with the diagnosis of primary gliomas, where there is a maximum potential benefit to the health service.

When used as a triage tool in primary care, this novel test has the potential to deliver improvements in health outcomes and also to reduce costs. At the lower end of test costs, the technology would be cost-saving for the health service. At higher test costs the technology is still likely to be considered cost-effective in HTA agency decision processes.

In Scenario 2, in which serum spectroscopy is used as a triage tool in secondary care, the technology will create additional costs but also produce sizable health benefits. At test costs of under £100 ($200) the technology would be likely to be considered as a cost-effective use of resources in HTA agency decision processes in the UK (and USA).

Sensitivity analyses have demonstrated the importance of diagnostic performance on the cost-effectiveness of the test. In particular, test specificity is important in the primary care setting. If test specificity is 87·5% or worse the technology may not be considered cost-effective at higher values of assumed test cost. This is due to the increased number of false positive results in this low prevalence population, generating a greatly increased proportion of fast-track imaging studies which increases costs.

To strengthen the case that this approach represents a cost-effective use of healthcare resources it is necessary to establish the diagnostic performance of the test prospectively. This can be accomplished by a suitably large cohort study in which serum spectroscopy is used alongside current clinical practice in one of the patient groups included in this evaluation. It would be appropriate to initially target the secondary care patient population, because the higher prevalence of disease in this group will reduce the sample size needed to accurately estimate diagnostic performance.

Decision makers are often most interested in patient outcomes, such as survival, rather than intermediate outcomes, such as accuracy or speed of diagnosis (although this latter point is vital for treatment of high grade gliomas). From this perspective, a randomized trial, or a prospective cohort study with extended follow-up, may be required to fully establish the size of survival and quality-of-life benefits of including a serum spectroscopy test in the diagnostic pathway. A trial with primary outcomes relating to survival and quality-of-life would be specific to either the primary or secondary care setting (rather than generalisable to both), would need a large sample size, and would also require a follow-up period to capture survival benefits. In the case of malignant glioma this would require a period of at least 24

For peer review only

months. Such a trial would clearly be expensive, time consuming and may be unfeasible. Decision makers may be willing to make a decision on implementation of the blood test based on the modeled effects of improvements in intermediate outcomes on later patient outcomes. In this situation, the model proposed in this evaluation, populated with diagnostic performance and other data from a prospective trial, could be used to inform decisions about the wider adoption of the technology.

Future developments beyond trials such as emerging epidemiological evidence and new technologies should also be included in any future evaluations. It was not possible to foresee and include all such possible scenarios in this early evaluation but that should not preclude assessment in the light of new evidence. Updated analysis should inform any decisions about system wide implementation.

Several results in this analysis suggest cost savings through reduced use of imaging for patients with a negative test result. To make the case that a serum spectroscopic test can improve the efficiency of the diagnostic pathway prospective studies will also need to explore the impact of these test results on clinician and patient imaging study decisions. The possibility remains that the test may triage patients, but may not reduce the number of scans being conducted, and could potentially increase the demand on imaging. However, this triaging of patients would still benefit each patient that is provided with an early diagnosis.

This evaluation has explored the potential for serum spectroscopy to be a cost-effective addition to the diagnostic pathway for brain tumours. It has demonstrated that in specific scenarios this novel test may be an effective and cost-effective technology in reducing the delay to diagnosis for patients with brain tumours. Prospective trials are required to provide definitive evidence.

For peer review only

References

1 Burnet, N., Jefferies, S., Benson, R., Hunt, D. & Treasure, F. Years of life lost (YLL) from cancer is an important measure of population burden—and should be considered when allocating research funds. *Brit J Cancer* 2005; **92**: 241–5.

2 Hands, J. R., Clemens, G., Stables, R. *et al.* Brain tumour differentiation: rapid stratified serum diagnostics via attenuated total reflection Fourier-transform infrared spectroscopy. *J Neurooncol* 2016; **127**: 463–72.

3 Smith, B. R., Ashton, K. M., Brodbelt, A. *et al.* Combining random forest and 2D correlation analysis to identify serum spectral signatures for neuro-oncology. *Analyst* 2016; **141**: 3668–78.

4 Finding a better way? Improving the quality of life for people affected by brain tumours, Report Published by The Brain Tumour Charity, 2014.

5 Aggarwal, A., Herz, N., Campbell, P. *et al.* Diagnostic delay and survival in high-grade gliomas - evidence of the 'waiting time paradox'? *Br J Neurosurg* 2015; **29**: 520–3.

6 Zhou, Y., Abel, G. A., Hamilton, W. *et al.* Diagnosis of cancer as an emergency: a critical review of current evidence. *Nat Rev Clin Oncol* 2017; **14**: 45–56.

7 McPhail, S., Elliss-Brookes, L., Shelton, J. *et al.* Emergency presentation of cancer and short-term mortality. *Brit J Cancer* 2013; **109**: 2027–34.

8 Blanks, R., Moss, S., McGahan, C., Quinn, M. & Babb, P. Effect of NHS breast screening programme on mortality from breast cancer in England and Wales, 1990-8: comparison of observed with predicted mortality. *BMJ* 2000; **321**: 665–9.

9 UK Colorectal Cancer Screening Pilot Group. Results of the first round of a demonstration pilot of screening for colorectal cancer in the United Kingdom. *BMJ* 2004; **329**: 133.

10 Quinn, M., Babb, P., Jones, J. & Allen, E. Papers Effect of screening on incidence of and mortality from cancer of cervix in England: evaluation based on routinely collected statistics. *BMJ* 1999; **318**: 904.

11 Frankel, S., Smith, G. D., Donovan, J. & Neal, D. Screening for prostate cancer. *The Lancet* 2003; **361**: 1122–8.

12 Gadalla, S., Pfeiffer, R., Kristinsson, S. *et al.* Brain tumors in patients with myotonic dystrophy: a population□based study. *Eur J Neurol* 2016; **23**: 542–47.

13 Callaghan, B. C., Kerber, K. A., Pace, R. J., Skolarus, L. E. & Burke, J. F. Headaches and neuroimaging: high utilization and costs despite guidelines. *JAMA Intern Med* 2014; **174**: 819–21.

14 National Institute for Health and Care Excellence, 2015. Suspected cancer: recognition and referral. NICE Guideline (MG12).

15 Livermore, L. J., Ma, R., Bojanic, S. & Pereira, E. A. Yield and complications of frame-based and frameless stereotactic brain biopsy–the value of intra-operative histological analysis. *Brit J Neurosurg* 2014; **28**: 637–44.

16 Weber, M. A., Zoubaa, S., Schlieter, M. *et al.* Diagnostic performance of spectroscopic and perfusion MRI for distinction of brain tumors. *Cancer Imaging* 2006; **6**: S32–S41.

17 Baker, M. J., Trevisan, J., Bassan, P. *et al.* Using Fourier transform IR spectroscopy to analyze biological materials. *Nature Protoc* 2014; **9**: 1771–91.

18 Lovergne, L., Bouzy, P., Untereiner, V. *et al.* Biofluid infrared spectro-diagnostics: pre-analytical considerations for clinical applications. *Faraday Discuss* 2016; **187**: 521–37.

For peer review only

19 Baker, M. J., Hussain, S. R., Lovergne, L. *et al.* Developing and understanding biofluid vibrational spectroscopy: a critical review. *Chem Soc Rev* 2016; **45**: 1803–18.
20 Hochberg, F. H., Atai, N. A., Gonda, D. *et al.* Glioma diagnostics and biomarkers: an ongoing challenge in the field of medicine and science. *Expert Rev Mol Diagn* 2014; **14**: 439–52.
21 Hands, J. R., Abel, P., Ashton, K. *et al.* Investigating the rapid diagnosis of gliomas from serum samples using infrared spectroscopy and cytokine and angiogenesis factors. *Anal Bioanal Chem* 2013; **405**: 7347–55.
22 Wang, H. Z., Simonson, T. M., Greco, W. R. & Yuh, W. T. C. Brain MR Imaging in the Evaluation of Chronic Headache in Patients without Other Neurologic Symptoms. *Acad Radiol* 2001; **8**: 405–8.
23 Sempere, A. P., Porta-Etessam, J., Medrano, V. *et al.* Neuroimaging in the evaluation of patients with non-acute headache. *Cephalalgia* 2005; **25**: 30–5.
24 Clarke, C. E., Edwards, J., Nicholl, D. J. & Sivaguru, A. Imaging results in a consecutive series of 530 new patients in the Birmingham Headache Service. *J Neurol* 2010; **257**: 1274–9.
25 Kernick, D., Stapley, S., Goadsby, P. J. & Hamilton, W. What happens to new-onset headache presented to primary care? A case-cohort study using electronic primary care records. *Cephalalgia* 2008; **28**: 1188–95.
26 Hamilton, W. & Kernick, D. Clinical features of primary brain tumours: a case–control study using electronic primary care records. *Brit J Gen Pract* 2007; **57**: 695–9.
27 Do, V., Gebski, V. & Barton, M. The effect of wating for radiotherapy for grade II/IV gliomas. *Radiother Oncol* 2000; **57**: 131–6.
28 Burnet, N., Jefferies, S., Benson, R., Hunt, D. & Treasure, F. Years of life lost (YLL) from cancer is an important measure of population burden—and should be considered when allocating research funds. *Brit J Cancer* 2005; **92**: 241–45.
29 Garside, R., Pitt, M., Anderson, R. *et al.* The effectiveness and cost-effectiveness of carmustine implants and temozolomide for the treatment of newly diagnosed high-grade glioma: a systematic review and economic evaluation. *Health Technology Assessment* 2007; **11**.
30 National Institute for Health and Care Excellence, 2013. Guide to the methods of technology appraisal. NICE Guideline (PMG9).
31 Simpson, G. C., Forbes, K., Teasdale, E., Tyagi, A. & Santosh, C. Impact of GP direct-access computerised tomography for the investigation of chronic daily headache. *Br J Gen Pract* 2010; **60**: 897–901.
32 Taylor, T., Evangelou, N., Porter, H., Hamilton, W. & Kernick, D. Headache: two views on the right approach in general practice. *Br J Gen Pract* 2014; **64**: 475–6.
33 Taylor, T. R., Evangelou, N., Porter, H. & Lenthall, R. Primary care direct access MRI for the investigation of chronic headache. *Clin Radiol* 2012; **67**: 24–7.
34 White, P., Halliday-Pegg, J. & Collie, D. Open access neuroimaging for general practitioners - diagnostic yield and influence on patient management. *Brit J Gen Pract* 2002; **52**: 33–35.
35 UK Statistics Authority. Cancer Registration Statistics, England, 2014. Office of National Statistics

FIGURE LEGENDS

Figure 1. The diagnostic pathway of brain tumours. Timings relate to the diagnosis of high grade gliomas and are based upon discussion with the Clinical Focus Team and Aggarwal et al. (2015)[5]. (*OPD – Outpatient department; MDT – Multidisciplinary team*)

Figure 2. Attenuated total reflectance (ATR)-Fourier transform Infrared (FTIR) spectroscopic test of blood serum for the diagnosis and stratification of brain tumours using machine learning algorithms.

Figure 3. An unprocessed spectrum derived from human blood serum using ATR-FTIR spectroscopy. Spectral regions correspond to known bond vibrations and can therefore be associated with groups of biomolecules such as protein, lipid, phosphate and carbohydrates. Broad examples of blood serum constituents are listed.

Figure 4. A decision tree model describing the integration of a serum spectroscopy test in the current diagnostic pathway, and the effect on MRI/CT imaging for suspected brain tumour.

Figure 5 – Incremental cost effectiveness ratios (ICERs) at various specificities in primary care ('Scenario 1'). The £30,000 ICER threshold is displayed as a dashed horizontal line.

Figure 6 - Incremental cost effectiveness ratios (ICERs) various specificities in Secondary Care ('Scenario 2'). The £30,000 ICER threshold is displayed as a dashed horizontal line.

Figure 7 – Cost effectiveness acceptability curve (CEAC) at £50 and £100 per test – Primary care ('Scenario 1')

Figure 8 – Cost effectiveness acceptability curve (CEAC) at £50 and £100 per test – Secondary care ("Scenario 2")

For peer review only

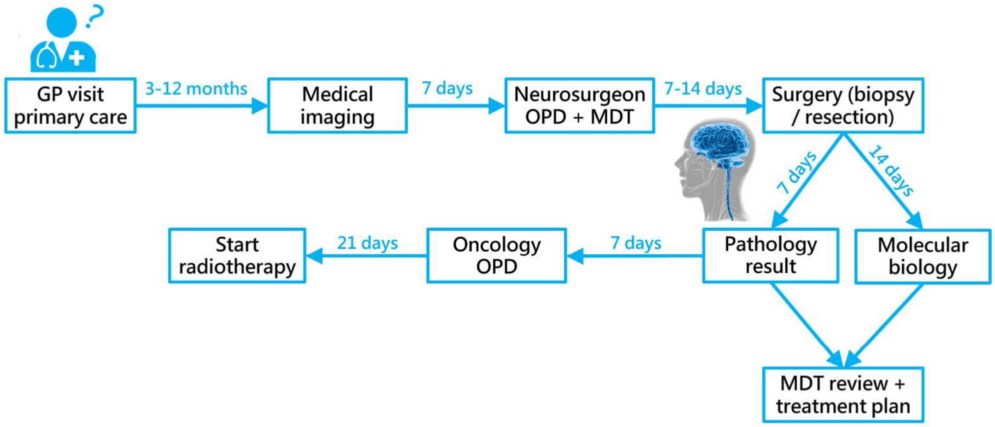

Figure 1. The diagnostic pathway of brain tumours. Timings relate to the diagnosis of high grade gliomas and are based upon discussion with the Clinical Focus Team and Aggarwal et al. (2015)5. (OPD – Outpatient department; MDT – Multidisciplinary team)

169x77mm (300 x 300 DPI)

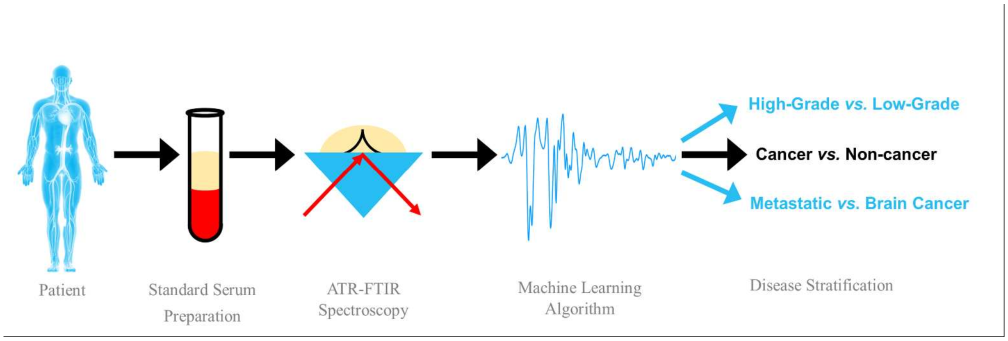

Figure 2. Attenuated total reflectance (ATR)-Fourier transform Infrared (FTIR) spectroscopic test of blood serum for the diagnosis and stratification of brain tumours using machine learning algorithms.

210x70mm (300 x 300 DPI)

peer review only

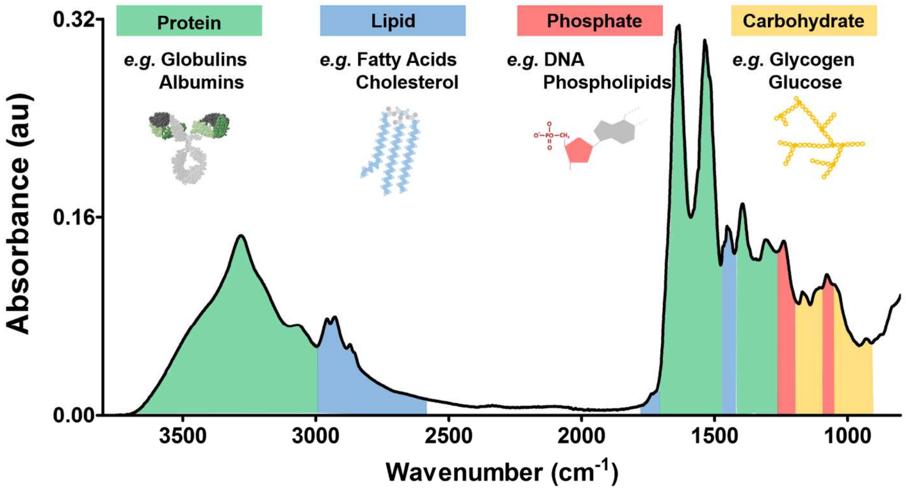

Figure 3. An unprocessed spectrum derived from human blood serum using ATR-FTIR spectroscopy. Spectral regions correspond to known bond vibrations and can therefore be associated with groups of biomolecules such as protein, lipid, phosphate and carbohydrates. Broad examples of blood serum constituents are listed.

169x95mm (300 x 300 DPI)

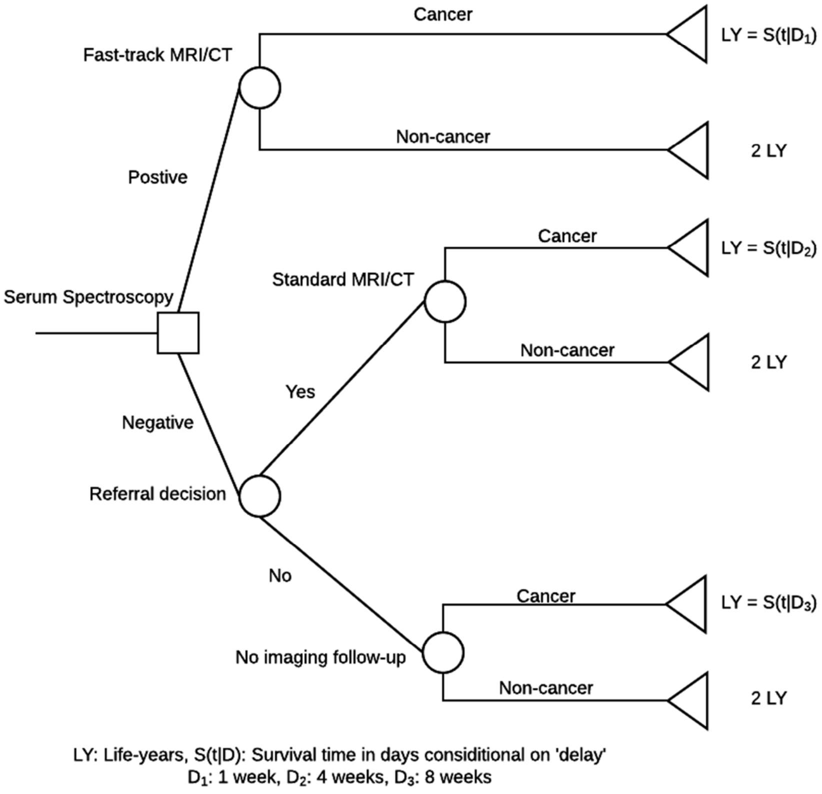

Figure 4. A decision tree model describing the integration of a serum spectroscopy test in the current diagnostic pathway, and the effect on MRI/CT imaging for suspected brain tumour.

82x74mm (300 x 300 DPI)

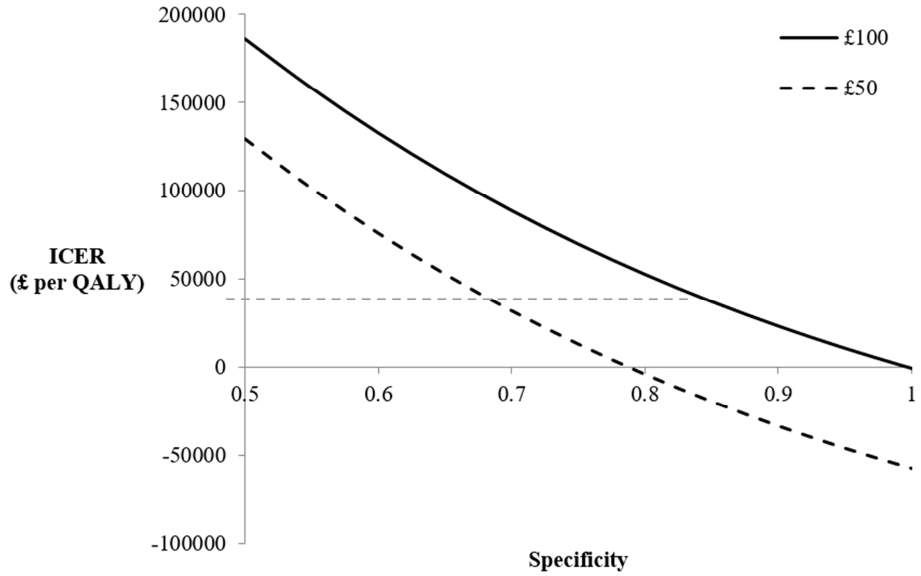

Figure 5 – Incremental cost effectiveness ratios (ICERs) at various specificities in primary care (‘Scenario 1’). The £30,000 ICER threshold is displayed as a dashed horizontal line.

78x49mm (300 x 300 DPI)

view only

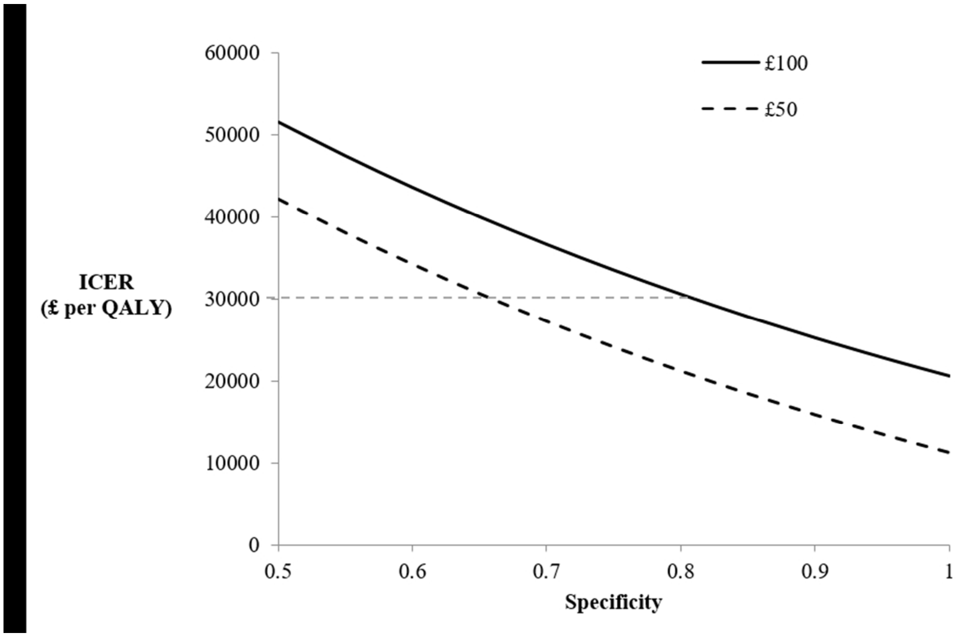

Figure 6 - Incremental cost effectiveness ratios (ICERs) various specificities in Secondary Care ('Scenario 2'). The £30,000 ICER threshold is displayed as a dashed horizontal line.

80x50mm (300 x 300 DPI)

view only

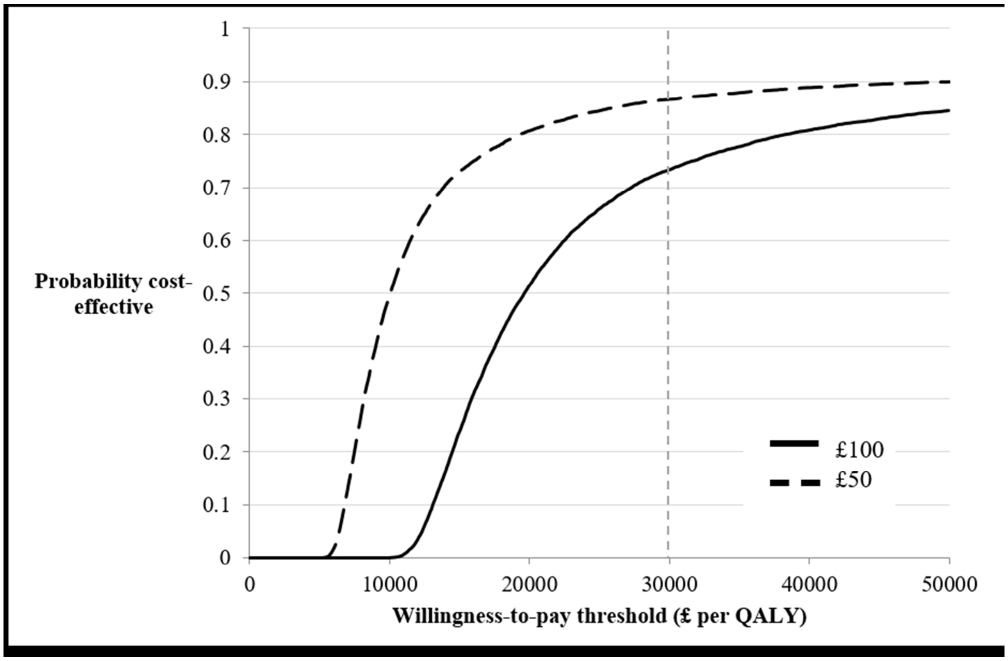

Figure 7 – Cost effectiveness acceptability curve (CEAC) at £50 and £100 per test – Primary care ('Scenario 1')

78x51mm (300 x 300 DPI)

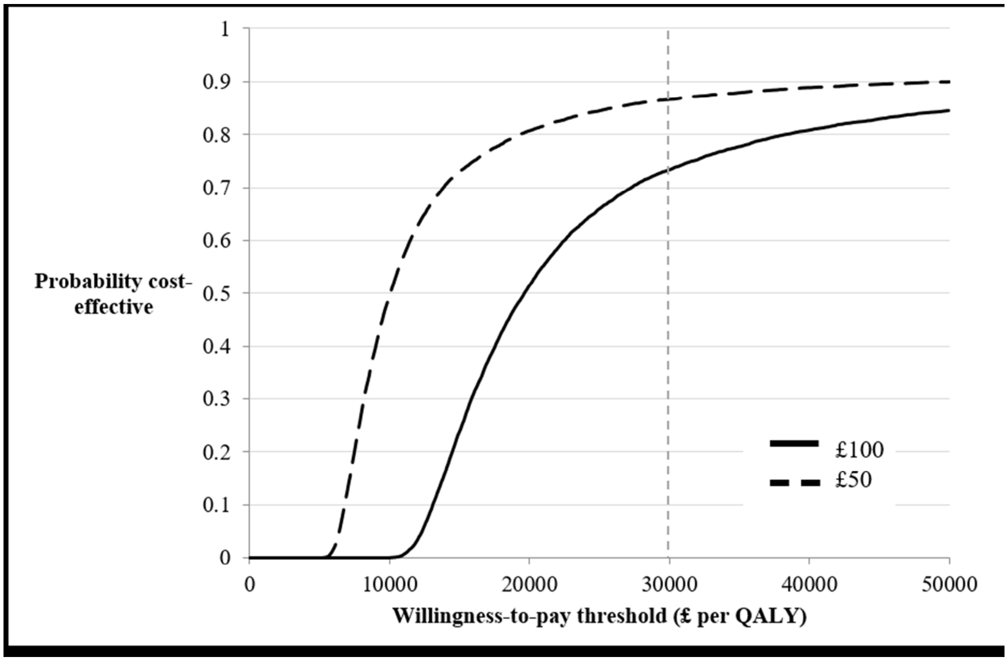

Figure 8 – Cost effectiveness acceptability curve (CEAC) at £50 and £100 per test – Secondary care ("Scenario 2").

78x51mm (300 x 300 DPI)

SUPPLEMENTARY MATERIAL

Appendix 1 – Clinical expert group

Clinical expert group participants: Mr Michael D Jenkinson (Walton Centre NHS Trust & University of Liverpool), Dr Paul Brennan (University of Edinburgh), Prof. Anthony Chalmers (University of Glasgow & Beatson Centre), Prof. Willie Hamilton (University of Exeter Medical School), Mr Kevin O'Neill (Imperial College Healthcare NHS Trust), Dr Allan Janes (Beatson West of Scotland Cancer Centre), Dr Babar Vaqas (Imperial College Healthcare NHS Trust), Dr David Kernick (St Thomas Health Centre, Exeter), Dr James Livermore (University of Oxford), Dr Ruth Board (Lancashire Teaching Hospitals NHS Trust), Prof. Timothy Dawson (Lancashire Teaching Hospitals NHS Trust), Miss Elvira Lekka (Lancashire Teaching Hospitals NHS Trust), Mr John Goodden (Leeds Teaching Hospital NHS Trust) and Dr. Samantha Mills (Walton Centre NHS Trust)

For peer review only

Appendix 2 – Decision tree model

The decision node probabilities that populate the decision tree model are reported in Table S1. The model is simplified by assuming that the reference test (MRI/CT) has perfect accuracy (100% sensitivity and 100% specificity). This is desirable if we wish to evaluate the test against an MRI/CT only diagnostic pathway in which these imaging studies are considered as the 'gold standard'. It is also difficult to obtain valid estimates of MRI/CT sensitivity and specificity for this indication as these are not reported in a comparable manner in the literature. This simplification will not substantially alter the economic results compared to using more realistic values for the scenarios under consideration. This is because it is assumed confirmation of the diagnosis always requires imaging and therefore outcomes for patients that would hypothetically be classified correctly using this test but incorrectly by MRI/CT will remain the same whether or not the test is used. The effect of less than perfect accuracy of the reference test is therefore the same as a (small) decrease in prevalence as a proportion of patients with the disease cannot benefit from the additional test due to incorrect subsequent testing. Outcomes are calculated by 'rolling-back' the tree for both cancer cases and non-cases and taking an average of the two groups weighted by the prevalence of brain tumours in the scenario population.

Table S1. Decision node probabilities for decision tree model describing the integration of a serum spectroscopy test in the current diagnostic pathway

**Cancer cases:**	**Probability to upper branch**	**Probability to lower branch**
Serum Spectroscopy Test (test sensitivity)	0·928	0·072
Standard MRI/CT (reference test sensitivity)	1	0
Fast-track MRI/CT (reference test sensitivity)	1	0
Referral decision	S1:0·5, S2: 1	S1:0·5, S2: 0
Non-cases:		
Serum Spectroscopy Test (test specificity)	0·085	0·915
Standard MRI/CT (reference test specificity)	0	1
Fast-track MRI/CT (reference test specificity)	0	1
Referral decision	S1:0·5, S2: 1	S1:0·5, S2: 0

S1 & S2: Scenarios 1 &2

For peer review only

Appendix 3 – Assumptions and details of cost effective analysis model

Effect of testing on time-to-diagnosis and time-to-treatment

To estimate the expected time-to-diagnosis under a fast-track pathway it is assumed that this would match the current median time-to-diagnosis for patients presenting with brain tumour in emergency care as observed in Aggarwal et al [5]. This study reported a cohort of high grade glioma patients from a single hospital in London, UK. No other suitable estimates of time-to-treatment or time-to-diagnosis were identified in the literature. This is the most common primary brain tumour in the indications considered in this evaluation. Median time-to-diagnosis for the standard pathway is also taken from the same source.

Effect of testing on use of imaging studies

The effect of spectroscopic testing on imaging study decisions of clinicians and patients is uncertain. Based on clinical expert input it was assumed, in the base case, that in secondary care all patients would continue to imaging, while in primary care 50% would continue to imaging following a negative spectroscopy result.

Effect of early diagnosis on patient outcomes

A systematic review of the literature failed to identify any studies that directly estimated the effect of earlier (or later) diagnosis on outcomes for primary brain tumours. Instead, based clinical expert guidance, estimates of the effects of time-to-treatment were sourced from the literature. The estimated effects are based on fitting natural history models to observational datasets of high grade glioma patients.[27,28] It is necessary to use a natural history model to produce survival estimates rather than using the survival data stratified by time-to-treatment directly, in order to adjust for potential confounding factors influencing both time-to-treatment and survival.

A one-to-one correspondence is assumed for the effects of an additional day added to the time-to-diagnosis and an additional day added to the time-to-treatment. Median survival by weeks between diagnosis and treatment are displayed in Table 2. The hazard ratio per additional day delay calculated in Do et al of 1·015 was applied to each day between diagnosis and treatment up to 28 days to calculate median survival time. Time between diagnosis and treatment beyond 28 days were fixed at the same median survival as 28 days.

Table S2. Relationship between time-to-diagnosis and survival

Time-to-diagnosis (weeks)	Median survival (weeks)
0	46
1	41·4
2	37·3
3	33·6
4	30·3

Source: Do et al 2000

Resource use and cost

It is assumed that there is no additional cost for converting a small proportion of standard referral to additional urgent referrals. This could be justified on the basis that patients receiving negative results allows more flexibility in the scheduling for these patients. This could create more opportunity to schedule patients with positive spectroscopy results sooner. As a larger proportion of patients are referred for immediate imaging it was assumed costs would increase because achieving this would require additional capacity. It was assumed that unit costs for MRI and CT grow exponentially with increasing proportion of immediate referrals such that costs double if 50% of patients are referred for immediate imaging.

Treatment costs are assumed to be the same for both fast-track and standard referrals that are diagnosed as cases. This may be a conservative assumption as treatment costs are likely to increase as disease progresses and therefore earlier treatment may, on average, be less costly than later treatment. However, it is also possible that

For peer review only

treatment costs could be increased if some cases are converted from palliative care to more aggressive treatment. The balance of these effects is unknown given the lack of appropriate data to estimate the effects. Secondary treatment and end-of-life costs are similarly assumed to be equal between fast track and standard referrals. This reflects the model assumption that all patients ultimately progress.

The time intervals considered (0-4 weeks) are too short for discounting to have an important impact on cost estimates therefore the effects of moving forward treatment on present value is not considered.

For peer review only

Appendix 4 - Total demand for serum spectroscopy tests in UK in two clinical scenarios

1. Primary care

An assumption that patients currently referred in direct-access imaging service would be selected for spectroscopic testing can provide a conservative estimate of the total number of patients likely to be tested. Three sources from the literature were considered:

1. A direct-access brain MRI service in Nottingham (UK) reported 130 patients per quarter (520 per year) among a population of 342,000.[32,33] The population of the UK is approximately 61 million therefore, assuming Nottingham is representative of the UK general population demand for brain MRI, there would be approximately 93,000 per year in the UK.
2. A direct access CT service for chronic headache in Glasgow (UK) reported 4404 patients referred over 8 years (551 per year) among a population of approximately 1 million.[31] Assuming this was representative of the UK generally then the demand for this type of service would be approximately 34,000 for the UK.
3. A direct access CT neuroimaging service in Lothian (UK) reported 389 exams per year for a population of approximately 442,000.[34] Assuming this was representative of the UK generally then the demand for this type of service would be approximately 54,000 for the UK.

The Glasgow estimate is particularly low and may result from limited uptake in the early part of the 8 year period for which data were reported. As it was not possible to determine if uptake increased over the period or was stable this estimate was not considered reliable. The Nottingham and Lothian estimates report potentially more stable and established services and are therefore more likely to reliably estimate demand. The likely demand for this novel test, if the indication is considered identical to neuroimaging, is therefore between 54,000 and 93,000. This is likely to be conservative as the test may well have a slightly broader indication than current neuroimaging. A reasonable point estimate may be approximately 75,000.

2. Secondary Care

An estimate of the total demand for serum spectroscopic tests in the UK can be made based on the reported incidence of brain tumour in the UK and the proportion of cases currently diagnosed through secondary (non-emergency) care. Annual incidence of malignant brain tumours in the UK is approximately 4,700.[35] Approximately one third of these are likely to present through secondary, non-emergency care. Using the positive predictive value for patients currently referred to neuroimaging in this setting of 3% suggests that for each case there will be 33 non-case patients receiving neuroimaging. In total, in the UK, this would imply 53,000 patients per year in secondary care may be suitable for spectroscopic testing.

For peer review only

Appendix 5 - Sensitivity Analysis

One-way sensitivity analysis (OWSA)

OWSA results for a range of test sensitivity are displayed in S1 and S2 as a series of points on a cost-effectiveness (CE) plane with incremental QALYs (intervention-control) on the x-axis and incremental costs (intervention - control) on the y-axis. Corresponding OWSA results for a range of test specificities in primary and secondary care are shown in Figure 3 and 4 in the main article text respectively. These are displayed with the ICER on the y-axis and the test specificity on the x-axis. Note that the estimated QALYs do not change with specificity in the model therefore changes in the ICER are due solely to changes in incremental costs.**Error! Reference source not found.** All OWSA analyses show results assuming the spectroscopy based test costs of £50 and £100.

Figure S1 – Results on CE plane over a range of test sensitivities (0·5-0·99) – Primary care

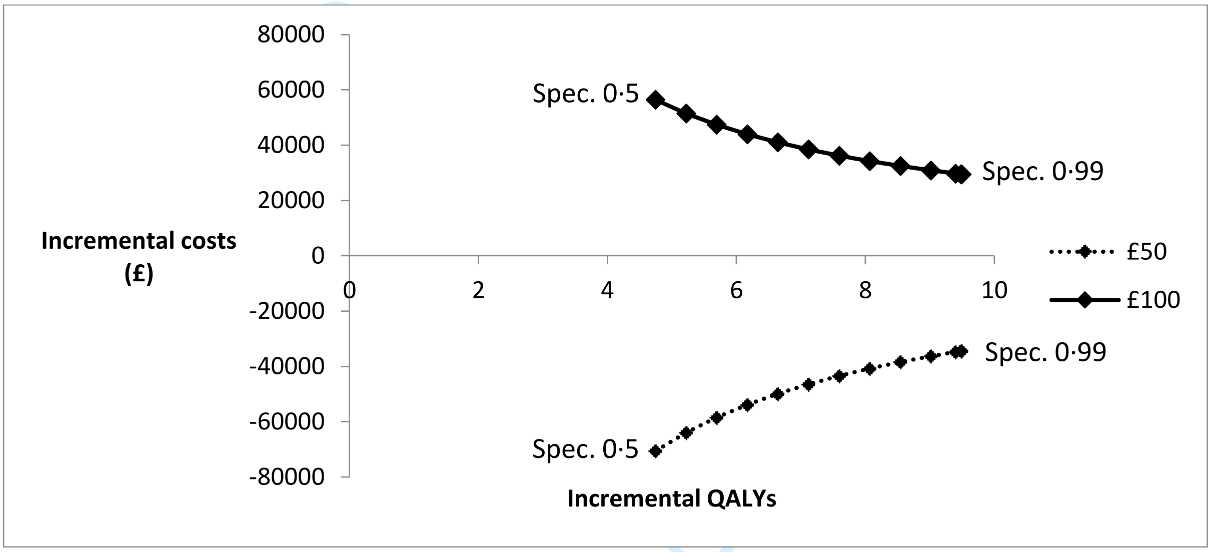

Figure S2 - Results on CE plane over a range of test sensitivities (0·5-0·99) – Secondary care

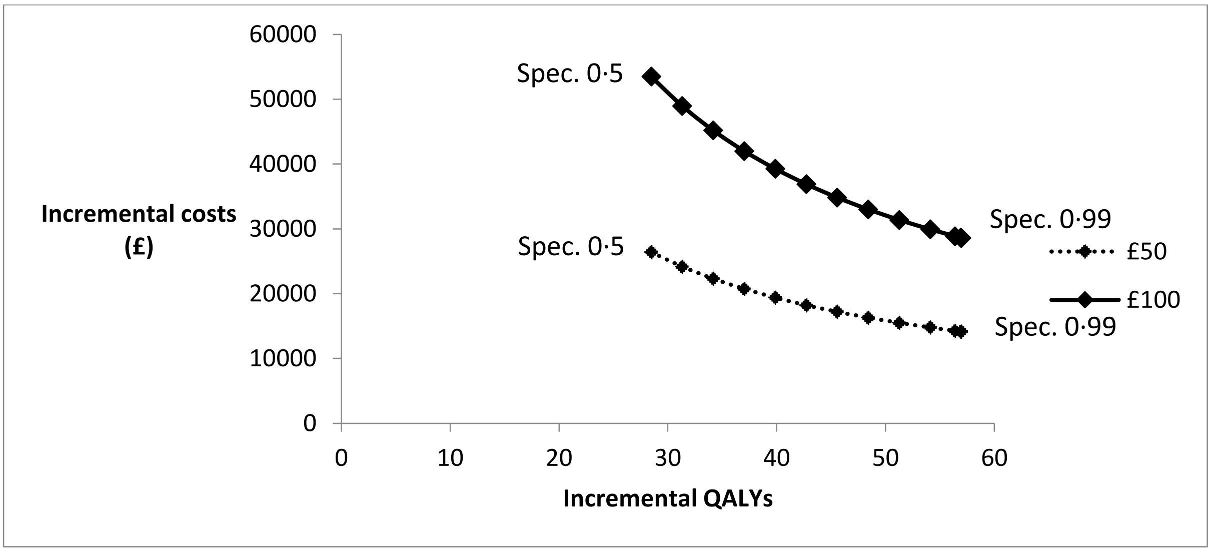

Scenario analysis

Table S3 – Scenario analysis: Mean survival estimates

	Scenario 1			Scenario 2		
Serum spectroscopy test cost (£)	ΔQALY	ΔCost	ICER	ΔQALY	ΔCost	ICER
50	12·83	-212808	-16582 (dominates)	77	540273	7016
100	12·83	287192	22377	77	1040273	13509

Table S4 - Additional costs scenario analysis

	Scenario 1			Scenario 2		
Serum spectroscopy test cost (£)	ΔQALY	ΔCost	ICER	ΔQALY	ΔCost	ICER
50	8·81	-379962	-43128 (dominates)	52·86	566247	10712
100	8·81	120038	13625	52·86	1066247	20171

Table S5 - Alternative prevalence estimates (1% prevalence) in primary care setting (Scenario 1)

	Scenario 1		
Serum spectroscopy test cost (£)	ΔQALY	ΔCost	ICER
50	17·62	-413220	-23452 (dominates)
100	17·62	86780	4925

Table S6– Alternative proportion of test negative cases referred to imaging (Scenario 1 only)

		Scenario 1		
Serum spectroscopy test cost (£)	% of test negative cases referred	ΔQALY	ΔCost	ICER
50	50 (base)	8·81	-422,116	-47,913
	75	17·62	-131,050	-7,438
	90	17·62	38,252	2,171
	100	17·62	151,120	8,577
100	50 (base)	8·81	77,884	8,840
	75	17·62	368,950	20,939
	90	17·62	538,252	30,548
	100	17·62	651,120	36,953

For peer review only

CHEERS Checklist

Items to include when reporting economic evaluations of health interventions

The **ISPOR CHEERS Task Force Report**, *Consolidated Health Economic Evaluation Reporting Standards (CHEERS)—Explanation and Elaboration: A Report of the ISPOR Health Economic Evaluations Publication Guidelines Good Reporting Practices Task Force*, provides examples and further discussion of the 24-item CHEERS Checklist and the CHEERS Statement. It may be accessed via the *Value in Health* or via the ISPOR Health Economic Evaluation Publication Guidelines – CHEERS: Good Reporting Practices webpage: http://www.ispor.org/TaskForces/EconomicPubGuidelines.asp

Section/item	Item No	Recommendation	Reported on page No/ line No
**Title and abstract**			
Title	1	Identify the study as an economic evaluation or use more specific terms such as "cost-effectiveness analysis", and describe the interventions compared.	1 / 1
Abstract	2	Provide a structured summary of objectives, perspective, setting, methods (including study design and inputs), results (including base case and uncertainty analyses), and conclusions.	3 / 1-29
**Introduction**			
Background and objectives	3	Provide an explicit statement of the broader context for the study.	5/2-12
		Present the study question and its relevance for health policy or practice decisions.	8/17-29
**Methods**			
Target population and subgroups	4	Describe characteristics of the base case population and subgroups analysed, including why they were chosen.	14/ 11-37
Setting and location	5	State relevant aspects of the system(s) in which the decision(s) need(s) to be made.	14/11-37
Study perspective	6	Describe the perspective of the study and relate this to the costs being evaluated.	26/3-32
Comparators	7	Describe the interventions or strategies being compared and state why they were chosen.	11-12/33-2
Time horizon	8	State the time horizon(s) over which costs and consequences are being evaluated and say why appropriate.	12/14-22
Discount rate	9	Report the choice of discount rate(s) used for costs and outcomes and say why appropriate.	12/2-3
Choice of health outcomes	10	Describe what outcomes were used as the measure(s) of benefit in the evaluation and their relevance for the type of analysis performed.	11/26-32
Measurement of effectiveness	11a	*Single study-based estimates:* Describe fully the design features of the single effectiveness study and why the single study was a sufficient source of clinical effectiveness data.	11/9-22

For peer review only

	11b	*Synthesis-based estimates:* Describe fully the methods used for identification of included studies and synthesis of clinical effectiveness data.	
Measurement and valuation of preference based outcomes	12	If applicable, describe the population and methods used to elicit preferences for outcomes.	9/1
Estimating resources and costs	13a	*Single study-based economic evaluation:* Describe approaches used to estimate resource use associated with the alternative interventions. Describe primary or secondary research methods for valuing each resource item in terms of its unit cost. Describe any adjustments made to approximate to opportunity costs.	11/33-40
	13b	*Model-based economic evaluation:* Describe approaches and data sources used to estimate resource use associated with model health states. Describe primary or secondary research methods for valuing each resource item in terms of its unit cost. Describe any adjustments made to approximate to opportunity costs.	12-13/4-4<br>24/1-17
Currency, price date, and conversion	14	Report the dates of the estimated resource quantities and unit costs. Describe methods for adjusting estimated unit costs to the year of reported costs if necessary. Describe methods for converting costs into a common currency base and the exchange rate.	11/38-40
Choice of model	15	Describe and give reasons for the specific type of decision-analytical model used. Providing a figure to show model structure is strongly recommended.	9/1
Assumptions	16	Describe all structural or other assumptions underpinning the decision-analytical model.	23-24/1-6
Analytical methods	17	Describe all analytical methods supporting the evaluation. This could include methods for dealing with skewed, missing, or censored data; extrapolation methods; methods for pooling data; approaches to validate or make adjustments (such as half cycle corrections) to a model; and methods for handling population heterogeneity and uncertainty.	12-13/4-4
**Results**			15/3-20
Study parameters	18	Report the values, ranges, references, and, if used, probability distributions for all parameters. Report reasons or sources for distributions used to represent uncertainty where appropriate. Providing a table to show the input values is strongly recommended.	
Incremental costs and outcomes	19	For each intervention, report mean values for the main categories of estimated costs and outcomes of interest, as well as mean differences between the comparator groups. If applicable, report incremental cost-effectiveness ratios.	15/3-20
Characterising uncertainty	20a	*Single study-based economic evaluation:* Describe the effects of sampling uncertainty for the estimated incremental cost and incremental effectiveness parameters, together with the impact	

For peer review only

		of methodological assumptions (such as discount rate, study perspective).	
	20b	*Model-based economic evaluation:* Describe the effects on the results of uncertainty for all input parameters, and uncertainty related to the structure of the model and assumptions.	17-18/2-2
Characterising heterogeneity	21	If applicable, report differences in costs, outcomes, or cost-effectiveness that can be explained by variations between subgroups of patients with different baseline characteristics or other observed variability in effects that are not reducible by more information.	
**Discussion**			
Study findings, limitations, generalisability, and current knowledge	22	Summarise key study findings and describe how they support the conclusions reached. Discuss limitations and the generalisability of the findings and how the findings fit with current knowledge.	19-20/4-20
**Other**			
Source of funding	23	Describe how the study was funded and the role of the funder in the identification, design, conduct, and reporting of the analysis. Describe other non-monetary sources of support.	2/28-33
Conflicts of interest	24	Describe any potential for conflict of interest of study contributors in accordance with journal policy. In the absence of a journal policy, we recommend authors comply with International Committee of Medical Journal Editors recommendations.	2/23-26

For consistency, the CHEERS Statement checklist format is based on the format of the CONSORT statement checklist

The **ISPOR CHEERS Task Force Report** provides examples and further discussion of the 24-item CHEERS Checklist and the CHEERS Statement. It may be accessed via the *Value in Health* link or via the ISPOR Health Economic Evaluation Publication Guidelines – CHEERS: Good Reporting Practices webpage: http://www.ispor.org/TaskForces/EconomicPubGuidelines.asp

The citation for the CHEERS Task Force Report is:
Husereau D, Drummond M, Petrou S, et al. Consolidated health economic evaluation reporting standards (CHEERS)—Explanation and elaboration: A report of the ISPOR health economic evaluations publication guidelines good reporting practices task force. Value Health 2013;16:231-50.

For peer review only

BMJ Open

Health economic evaluation of a serum based blood test for brain tumour diagnosis: Exploration of two clinical scenarios.

Journal:	*BMJ Open*
Manuscript ID	bmjopen-2017-017593.R2
Article Type:	Research
Date Submitted by the Author:	30-Jan-2018
Complete List of Authors:	Gray, Ewan; Health Improvement Scotland<br>Butler, Holly; Pure and Applied Chemistry; Technology & Innovation Building<br>Board, Ruth; Lancashire Teaching Hospitals NHS Foundation Trust, Rosemere Cancer Centre<br>Brennan, Paul; Western General Hospital, Department of Clinical Neurosciences<br>Chalmers, Anthony; University of Glasgow, Institute of Cancer Sciences; Beatson West of Scotland Cancer Centre<br>Dawson, Timothy; Lancashire Teaching Hospitals NHS Foundation Trust, Neurosurgery Department<br>Gooden, John; Leeds General Infirmary, Neurosurgery Department<br>Hamilton, Willie; University of Exeter Medical School, Primary Care Diagnostics<br>Hegarty, Mark; University of Strathclyde, Pure & Applied Chemistry<br>James, Allan; University of Glasgow Institute Of Molecular Cell And Systems Biology<br>Jenkinson, Michael; The Walton Centre NHS Foundation Trust<br>Kernick, David; St Thomas' Medical Group<br>Lekka, Elvira; Lancashire Teaching Hospitals NHS Foundation Trust, Neurosurgery Department<br>Livermore, Laurent; Oxford University Hospitals, NHS Foundation Trust, Department of Neurosurgery<br>Mills, Samantha; The Walton Centre NHS Foundation Trust<br>O'Neill , Kevin; Imperial College London<br>Palmer, David; University of Strathclyde, Pure & Applied Chemistry<br>Vaqas, Babar; Imperial College London, John Fulcher Neuro-Oncology Laboratory<br>Baker, Matthew; University of Strathclyde
**Primary Subject Heading**:	Health economics
Secondary Subject Heading:	Diagnostics, Health economics, Oncology
Keywords:	Biophysics < BASIC SCIENCES, HEALTH ECONOMICS, Neurology < INTERNAL MEDICINE, Neurological oncology < NEUROLOGY, Adult oncology < ONCOLOGY, Head & neck imaging < RADIOLOGY & IMAGING

For peer review only

Health economic evaluation of a serum based blood test for brain tumour diagnosis: Exploration of two clinical scenarios.

Ewan Gray[1†], Holly J. Butler[2,3†], Ruth Board[4], Paul M. Brennan[5], Anthony J Chalmers[6,7], Timothy Dawson[8], John Goodden[9], Willie Hamilton[10], Mark G. Hegarty[2,3], Allan James[11], Michael D. Jenkinson[12, 13], David Kernick[14], Elvira Lekka[8], Laurent J. Livermore[15], Samantha J. Mills[12], Kevin O'Neill[16], David S. Palmer[3,17], Babar Vaqas[16] Matthew J. Baker[2,3*]

[1] Health Improvement Scotland, NHS Scotland, Delta House, 50 West Nile Street, Glasgow, G1 2NP, UK; [2] WestCHEM, Department of Pure and Applied Chemistry, University of Strathclyde, Technology and Innovation Centre, 99 George Street, Glasgow, G1 1RD, UK; [3] ClinSpec Diagnostics Limited, Technology and Innovation Centre, 99 George St, G1 1RD, UK; [4] Rosemere Cancer Centre, Lancashire Teaching Hospitals NHS Trust, Royal Preston Hospital, Sharoe Green Lane, Preston, PR2 9HT, UK; [5] Department of Clinical Neurosciences, Western General Hospital, Edinburgh, EH4 2XU, UK; [6] Beatson West of Scotland Cancer Centre, 1053 Great Western Road, Glasgow, G12 0YN, UK; [7] Institute of Cancer Sciences, Wolfson Wohl Cancer Research Centre, University of Glasgow, Glasgow, G61 1QH; [8] Neurosurgery Department, Lancashire Teaching Hospitals NHS Trust, Royal Preston Hospital, Sharoe Green Lane, Preston, Lancashire, PR2 9HT, UK; [9] Neurosurgery Department, Leeds General Infirmary, Leeds, LS1 3EX, UK; [10] Primary Care Diagnostics, University of Exeter Medical School, College House, University of Exeter, Exeter, Devon, EX1 2LU, UK; [11] Institute of Molecular Cell and Systems Biology, Bower Building, Glasgow, G12 8QQ, UK; [12] The Walton Centre NHS Foundation Trust, Lower Lane, Fazakerley, Liverpool, L9 7LJ, UK; [13] Institute of Translational Medicine, Clinical Science Centre, University of Liverpool, Liverpool, L9 7LJ; [14] St Thomas' Medical Group, Cowick Street, Exeter, EX4 1HJ, UK; [15] Department of Neurosurgery, Oxford University Hospitals, NHS Foundation Trust, John Radcliffe Hospital, Oxford, OX3 9DU, UK; [16] John Fulcher Neuro-Oncology Laboratory, Imperial College, London, SW7 2AZ, UK; [17] WestCHEM, Department of Pure and Applied Chemistry, University of Strathclyde, Thomas Graham Building, 295 Cathedral Street, Glasgow, G1 1XL, UK

*Corresponding author: Dr Matthew J Baker, matthew.baker@strath.ac.uk, +44(0)141 548 4700, @ChemistryBaker

†These authors contributed equally to the paper

For peer review only

Copyright/Licence for publication

The Corresponding Author has the right to grant on behalf of all authors and does grant on behalf of all *authors,* a worldwide licence *to the Publishers and its licensees in perpetuity, in all forms, formats and media (whether known now or* created in the future), to i) publish, reproduce, distribute, display and store the Contribution, ii) translate the Contribution into other languages, create adaptations, reprints, include within collections and create summaries, extracts and/or, abstracts of the Contribution, iii) create any other derivative work(s) based on the Contribution, iv) to exploit all subsidiary rights in the Contribution, v) the inclusion of electronic links from the Contribution to third party material where-ever it may be located; and, vi) licence any third party to do any or all of the above

Authors' Contributions

EG conducted the health economic assessment including data analysis, data interpretation, and writing. HJB contributed to data interpretation, writing and providing details regarding clinical spectroscopy. MJB is the principal investigator of this research, and initiated this interdisciplinary project and team. RB, PMB, AJC, TD, JG, WH, AJ, MDJ, DK, EL, LJL, SJM, KO, and BV are members of the clinical focus group involved in spearheading translation; all of whom provided feedback on the manuscript. MGH, DSP, HJB and MJB are involved in the ClinSpec Diagnostics Limited project.

Competing Interest Declaration

MJB, MGH, HJB and DSP are all involved in ClinSpec Diagnostics Limited a prospective spin out company from the University of Strathclyde focusing on the translation of serum spectroscopic diagnostics (Company Number SC535447)

Role of the Funding Source

Scottish Enterprise is Scotland's main economic development agency and non-departmental public body of the Scottish Government. They work with partners in both the private and public sectors to exploit the best opportunities to deliver a significant, lasting effect on the Scottish economy. The High-Growth Spinout Programme helps researchers take their ideas and inventions from the lab to the global marketplace.

Acknowledgements

The authors acknowledge the support from Rosemere Cancer Foundation, Brain Tumour North West, EPSRC, and the Sydney Driscoll Neuroscience Foundation for funding.

For peer review only

Data Sharing Statement

No additional data available

For peer review only

Abstract

Objectives

To determine the costs and health benefits of a serum-based spectroscopic triage tool for brain tumours, that could potentially reduce diagnostic delays in the current clinical pathway.

Design

A model-based health economic assessment. Decision tree models were constructed based on simplified diagnostic pathways. Models were populated with parameters identified from rapid reviews of the literature and clinical expert opinion.

Setting

Explored as a test in both primary and secondary care (neuroimaging) in the UK health service, as well as application to the US.

Participants

Calculations based upon an initial cohort of 10,000 patients. In primary care, it is estimated that the volume of tests would approach 75,000 per annum. The volume of tests in secondary care is estimated at 53,000 per annum.

Main Outcome Measures

The primary outcome measure was quality adjusted life years (QALYs), which were employed to derive incremental cost-effectiveness ratios (ICERs) in a cost-effectiveness analysis.

Results

Results indicate that using a blood-based spectroscopic test in both scenarios may be considered highly cost-effective in a health technology assessment (HTA) agency decision making process, as ICERs were well below standard threshold values of £20,000 to £30,000 per QALY. This test may be cost-effective in both scenarios with test sensitivities and specificities as low as 80%; however, the price of the test would need to be lower (less than approximately £40).

Conclusion

Use of this test as triage tool in primary care has the potential to be both more effective and cost saving for the health service. In secondary care, this test would also be deemed more effective than the current diagnostic pathway.

For peer review only

Strengths and Limitations of this Study

- Simplified models of clinical pathways were mapped with input from, and consensus among, a wide range of clinical experts including neurosurgeons, neurooncologists, neuropathologists, neuroradiologists, and primary care experts.
- The spectroscopic blood test was highly sensitive and specific in retrospective data, with performances of 91.5 and 83.0% respectively. There is potential for this to contribute towards improved prognosis for patients, as well as healthcare savings.
- This study is based upon proof-of-concept studies, in advance of a pending prospective clinical trial. As these samples are retrospective, there is the possibility the diagnostic performance will not be as high in prospective studies.
- A lack of clinical trial evidence necessitates the estimate of long-term benefits of improved diagnostic protocols based on disease natural history models. This creates additional uncertainties.
- The precise patient population for whom the test may be suitable in the primary care setting is difficult to establish at this stage in development. This study considers a limited definition of eligibility that may need revision in light of future evidence.

For peer review only

Introduction

At an average of 20 years, patients with malignant brain tumours have the highest number of years of life lost, compared to all other primary cancers.[1] This, at least in part, may relate to diagnostic delays, reflecting the non-specific early symptoms, such as headache and dizziness, from which general practitioners (GPs) must identify patients at risk for further investigation. The lack of a low-cost diagnostic and/or screening tools available within the health service contributes to this delay. We have recently demonstrated that a spectroscopic test using blood-serum is able to effectively identify brain tumours in patients with sensitivities and specificities as high as 91·5% and 83% respectively.[2,3] This approach is based upon Fourier-transform infrared (FTIR) spectroscopy and can detect disease-specific signatures, which are extracted mathematically using pattern recognition and machine learning algorithms.

Current Diagnostic Pathway

Currently, patients who are symptomatic with a brain tumour visit their GP on average five times before being referred to secondary care.[4] Partly as a result of this diagnostic delay up to 61% of brain tumour diagnoses occur in an emergency setting, often following a seizure.[5] Patients diagnosed by the emergency route have a poorer prognosis.[6,7] For some patients this may be because the disease is at a more advanced stage at diagnosis. The complications precipitating the emergency admission make an additional contribution to mortality.

Screening programmes for breast, prostate, cervical, and colorectal cancer have proved effective for diagnosing patients at an earlier stage, which can result in a better prognosis.[8-11] These screening programmes have had a significant impact in reducing the number of patients presenting as an emergency. To date there has been no accessible and economically viable diagnostic tool for early detection of asymptomatic and symptomatic brain tumours.[12] The addition of a rapid and accurate blood test for patients with suspected primary brain tumours (symptomatic patients) therefore has the potential to improve outcomes by allowing prioritisation of patients most at risk of a brain tumour for further investigation. Under the current patient pathway it is not feasible to provide fast-track diagnostic imaging because the number of patients with non-specific headache symptoms is very large and the positive predictive value (PPV) on the basis of symptoms alone is less than 3% for all symptoms other than a new-onset seizure.[13,14]

Magnetic resonance imaging (MRI) and computed tomography (CT) are the current gold standard for identifying structural brain lesions including tumours. Treatment decisions made at the neuro-oncology multidisciplinary team (MDT) meeting are often based on the imaging alone. Following surgical resection or biopsy with histopathology and molecular analysis, definitive treatment can be planned.[15] Surgery to secure the tissue diagnosis has a small risk to the patient of neurological deterioration and death.[16]

The diagnostic pathway also represents a significant cost burden to the health service, with a single MRI and CT scan in the UK costing around £164 and £85, respectively (National Schedule of Reference Costs [2014-15], Medicare Physician Fee Schedule 2016). A typical

For peer review only

timeframe of the diagnostic pathway for brain tumours, specifically primary gliomas, is illustrated in Figure 1, and effectively highlights the significant wait that a symptomatic patient may have before receiving brain imaging. Even from this stage, regardless of the time to GP referral or emergency presentation, full diagnosis may take a further 5 weeks.

Serum Spectroscopic Diagnostics

This novel blood test for early brain tumour detection is based upon the interaction of infrared (IR) light with biological components of blood serum (**Figure 2**). Specifically, using attenuated total reflectance (ATR)-FTIR spectroscopy, specific bond vibrations of given molecules can be elucidated from serum samples, thus providing a unique insight into the composition via an absorbance spectrum.[17] Benefits of an ATR-FTIR based approach include a robust, user-friendly methodology without extensive sample preparation, that would readily fit into a clinical setting.[18] In short, serum is obtained according to standard protocols and can be snap frozen and stored at -80° until the point of analysis. A small volume of serum is required for analysis (1-5 μL), which is pipetted onto a crystal, known as an internal reflection element (IRE), where IR non-destructively interacts with the sample and produces an IR spectrum, with peaks representative of known bond vibrations and hence biomolecular constituents.

Blood serum is a complex medium that contains a variety of biomolecules, including around 20,000 proteins, that may be employed as diagnostic biomarkers.[19] In the case of brain tumours, such blood based technologies are limited, due to a lack of an established brain tumour specific diagnostic biomarker.[20] With our spectroscopic approach, rather than derive single biomarker specific information, a global signature is obtained, which encompasses the entire biomolecular makeup. This is epitomised as an equally complex biological absorbance spectrum, which contains a wealth of diagnostic information (an example may be seen in **Figure 3**).

Pattern recognition and machine learning algorithms using spectral features from FTIR data have been demonstrated as rapid and accurate for separating primary brain cancer and non-cancer cases.[2,21] When these algorithms are applied to this information rich dataset, the relationship between all biological components of the sample is ascertained, providing a multi-dimensional analysis of the sample.

This approach has been able to not only detect between cancer and non-cancer, but also stratify based upon cancer pathology.[2] For further information and in-depth description of the methodology, we direct the readers to the following fundamental review and recent research papers.[2,3,17,21] The ability to triage patients likely to have a brain tumour based on serum sample alone raises the possibility of systematic triaging prior to investigation with more expensive (MRI/CT imaging) and invasive (biopsy) tests. One major impact of having a serum test available would be a clear reduction in the number of unnecessary brain scans; however, as this test is also able to differentiate between primary and secondary tumours, there could also be a knock-on reduction of chest and abdomen scans which are conducted to rule out primary disease elsewhere. There is also the possibility that this approach will reduce

For peer review only

the incidence of incidental abnormalities which in themselves can cause considerable distress. Ultimately, it is expected that this could allow earlier and potentially more effective treatment of brain tumours.

It is important to note that this study is based upon proof-of-concept studies, in advance of a pending clinical trial. As these samples are retrospective, there is the possibility the diagnostic performance will not be as high in prospective studies. Additional to determining the true diagnostic accuracy of the technique, the planned clinical trial held in primary care will also reveal the suitable patient population for the test, as well as the long-term benefits of an improved diagnostic pathway.

Aims & Objectives

The aim of the economic evaluation is to assess the potential cost-effectiveness of this spectroscopic technology, in advance of any prospective study results being available. There are three main objectives for the evaluation. The first objective is to create a map of where the test could be used in the clinical pathway. The second is to assess the potential cost-effectiveness of the technology, if the performance shown in the case-control study is replicated prospectively. This will give an indication of whether the technology would meet the criteria for acceptance for use in the National Health Service that are applied by Health Technology Assessment agencies (HTA). Related to this, the third objective is to define the level of performance in prospective trials, and any additional evidence that would be needed to meet the cost-effectiveness criteria of an HTA decision-making process. This can include diagnostic performance and also effects on long-term outcomes such as survival and resource use. To achieve these objectives a simplified economic model of two important clinical scenarios is used to explore cost effectiveness.

For peer review only

Methodology

Mapping the Clinical Pathway

In order to appreciate the current clinical pathway and determine an appropriate entry point for a serum spectroscopic test, a pan-UK clinical focus group was established. This cohort included neurosurgeons, clinical and medical oncologists, neuropathologists, neuroradiologists, academic GPs with special interests, and experts in primary care diagnostics (see Appendix 1).

Cost Effectiveness Analysis

A cost-effectiveness analysis (CEA) was conducted to calculate the effects on health outcomes and health service costs of introducing spectroscopic testing in each of two scenarios. The health outcomes considered were life-years and quality-adjusted life-years (QALYs).

A decision tree was used to model the pathway for patients presenting with symptoms warranting a referral for MRI/CT imaging for suspected brain tumour (**Figure 4**). Separate models were considered for primary and secondary care. The time horizon of the model is 2 years and the perspective is that of the health care service. A two-year horizon was selected because of the short duration of survival in this patient group: median survival is approximately 1 year for high grade gliomas, which are the commonest malignant primary brain tumour. In all scenarios the comparator is the current diagnostic pathway (i.e. imaging alone). Further details regarding the node probabilities, simplifications and assumptions of the model can be found in **Appendix 2**.

Diagnostic Performance

The sensitivity and specificity of the test for detecting brain tumours has been demonstrated in a series of case-control studies using historical samples from the Brain Tumour North West and Walton Centre NHS bio-banks.[2,3] In the key case-control study nine FTIR spectra were collected from each of 433 patients.[3] Of these 134 were from patients with primary brain tumours (64 high grade glioma), 177 were from patients with cerebral metastases and 122 were from non-cancer controls. FTIR spectra were analysed using the random forest method to fit a classification model. Classification performance was estimated by applying the fitted model to a test set containing 20% of the patients from the original data set that were not used in the model fitting step (hold-out test set). Classification statistics are computed as averages of this process, iterated 96 times using random training and test sets. Under the best available model, sensitivity estimated by this method was 92·8% and specificity was 91·5% for the analysis of cancerous vs. non-cancerous serum.[3] These classification statistics were established using 96 independent iterations of a Random Forest model, and resulted in standard deviations of 1.1% and 1.9% respectively.

Establishing whether the performance demonstrated case-control data from historical samples translates to equivalent performance when applied prospectively in clinical practice is the subject of a planned clinical trial. This is critical to demonstrating the clinical and cost-

For peer review only

effectiveness of a serum spectroscopy test as part of the diagnostic pathway for brain tumours.

Prevalence of disease

Prevalence data were sourced from the literature based on clinical expert guidance. Brain tumour prevalence in scenario 2 (secondary care) was assumed to be 3% based on observed rates of primary brain tumour diagnosis among patients referred for brain imaging for suspected cancer in secondary care.[22-24] In scenario 1 (primary care) an estimated prevalence of 0·5% is used based on case-control evidence and expert opinion of the prevalence among patients who would be considered for direct access imaging, using MRI or CT, where this is available and referral to neurology where it is not.[25,26] An alternative prevalence of 1% was explored for scenario 1 based on unpublished data from a direct access imaging service in the UK (P. Brennan, personal communication).

The effect of serum spectroscopy testing on the time-to-diagnosis and time-to-treatment is discussed in Appendix 3, additional to the effect of testing on the use of imaging studies, and also on the patient outcomes. The primary assumptions in this model are that first, the expected time-to-diagnosis would match the current median time-to-diagnosis for patients presenting with brain tumour in emergency care as observed in Aggarwal et al.[5] Furthermore, based upon expert opinion, it is assumed that in secondary care all patients would continue to imaging, while in primary care 50% would continue to imaging following a negative spectroscopy result. This is a conservative estimate associated with the possibility that an imaging test will still be required in some cases based on interpretation of a patient's symptoms and the other non-tumour diagnoses being considered by the clinician. Finally, the effects of early diagnosis on the outcome of brain tumours is estimated using literature describing fitting natural history models to observational datasets of high grade glioma patients.[27,28]

Utility Weights

Health state utility weights are applied to life-years to generate quality adjusted life years (QALYs). A systematic review of health state utility weights for high grade glioma, the most common and aggressive primary brain tumour, was conducted to identify suitable utility weights. Due to heterogeneity it was not considered suitable to pool the estimates. The most appropriate health state utility weight was taken from a previous UK economic evaluation of glioblastoma treatment.[29] A value of 0·89 was used in the base case.

Resource Use and Costs

Resource use includes the application of a spectroscopic serum test to all patients prior to imaging, the imaging studies used in the diagnostic process, outpatient neurology clinic visits and general practitioner visits. In the UK analysis, unit costs for imaging studies are taken from UK NHS reference costs (2014/15), clinic and GP visits from the PSSRU costs schedule (Table 1). In the USA analysis, unit costs are taken from Medicare reimbursement schedules. Unit costs of the test were applied at an upper bound and lower bound rather than a single value as these products have not yet been commercialized. Bounds were set by consultation

For peer review only - http://bmjopen.bmj.com/site/about/guidelines.xhtml

with scientists developing the tests. Additional resource use and cost assumptions are described in Appendix 3.

Table 1. Unit costs and comparison for the brain tumour diagnostic pathway

Item	UK Cost per unit (£) (2015 prices)	USA cost per unit ($) (2016 prices)	Source(s)
CT imaging study [CT head]	85	163	National Schedule of Reference Costs (2014 – 15), Medicare Physician Fee Schedule 2016
MRI imaging study [MRI brain with contrast]	164	380	National Schedule of Reference Costs (2014 – 15), Medicare Physician Fee Schedule 2016
Neurology outpatient appointment	35	76	PSSRU 2016, Medicare Physician Fee Schedule 2016
GP visit	47·25	76	PSSRU 2016, Medicare Physician Fee Schedule 2016
Stable disease monitoring costs	116 per 3 months	154	[29], exchange rate adjusted
Serum spectroscopy test	Lower limit: 50<br>Upper limit: 100	Lower limit: 100<br>Upper limit: 200	Assumed prices

Incremental cost effectiveness ratios (ICER)

The comparative cost-effectiveness of spectroscopic testing compared to no testing is summarized by the ICER defined as:

$$ICER = \frac{C_s - C_n}{H_s - H_n}$$

C_s and C_n are the total costs with spectroscopic testing and no testing respectively. Equivalently H_s and H_n are the total QALYs with and without spectroscopic testing. The ICER can be interpreted as the additional cost per QALY gained.

For peer review only

Base Case and Sensitivity Analysis

ICERs were calculated for scenarios 1 and 2 using the base case parameter estimates. Base case analysis was repeated for UK and USA settings. Additional sensitivity analyses are reported for the UK setting only.

Sensitivity analyses included one-way sensitivity analysis (OWSA), systematically varying a single parameter in the model, and scenario analysis in which specific model assumptions were altered. OWSA were conducted for test sensitivity, specificity and test cost. Scenario analyses included assuming an additional consultation cost for discussion of test results, assuming a higher proportion of patients continue to imaging following a negative spectroscopy result in primary care, and using mean survival rather than median survival.

A probabilistic sensitivity analysis (PSA) was conducted to explore the effects of joint uncertainty in the parameter estimates on the model results.[30]

For peer review only

Results

Mapping the clinical pathway

For the first study objective, initial discussion with clinical experts indicated that there are potential uses for the test in both primary and secondary care. The main advantage of employing a cost-effective spectroscopic blood test in the diagnostic pathway is to use it as a triage test. This prioritises more urgent cases for access to services, and acts as a gate-keeper (requiring a positive result in some conditions to give access to services) for imaging studies.

Two clinical scenarios are mapped out below and subsequently explored in this early economic evaluation of the serum spectroscopy test for aiding diagnosis of brain tumours:

1. Triage tool in primary care

The primary care scenario explores a population of patients with a clinical presentation that warrants further investigation of possible brain tumour. This would include some patients with headaches and some with focal neurological deficits. This is the group of patients who would be considered for direct access imaging, using MRI or CT, where this is available and referral to neurology where it is not.[31] The blood test is used to provide rapid information, within 24 hours, where a positive result would lead to patients receiving more timely access to imaging. It may also be the case that negative test results, in addition to establishing the low probability of a brain tumour, could also provide some reassurance for those patients that must wait for imaging. The total volume of tests would be approximately 75,000 per year in the UK (see Appendix 4 for further details).

2. Triage tool in secondary care

In this scenario the population is the group of patients that are currently referred for imaging studies from secondary care for suspected brain tumour, typically via neurology clinics. This is the patient group for whom the clinical presentation has the highest positive predictive value (PPV). However, even in this high risk group, the odds of a brain tumour being present are approximately 1:33.[22-24] Again, the spectroscopy test is used to provide rapid information to allow a subset of these patients to access immediate imaging and provide reassurance to other patients who may have to wait longer for definitive imaging studies and diagnosis. The extent of the benefits of triage in this scenario is likely to vary by locality depending on the capacity constraints on imaging and pathology services. This evaluation uses estimates of the delays in diagnosis, and potential improvements in the speed of diagnosis, from a consecutive patient case series in London, UK.[5] The total volume of tests if this scenario occurred would be approximately 53,000 per year in the UK (see Appendix 4 for further details).

For peer review only

Cost Effectiveness Assessment

The standard threshold value per QALY gained in the UK, is considered to be between £20,000 to £30,000. Below this value, a healthcare intervention may be considered cost effective, whereas a negative ICER value would be deemed cost saving. Base case results for primary care (scenario 1) and secondary care (scenario 2) are presented in Table 2. Note results are reported for cohorts of 10,000 patients.

Table 2. Incremental QALYs, costs and ICERs for Scenarios 1 and 2, UK and USA

	**Scenario 1 – Primary Care**			**Scenario 2 – Secondary Care**		
**Serum spectroscopy test cost (£)**	**ΔQALY**	**ΔCost**	**ICER**	**ΔQALY**	**ΔCost**	**ICER**
**UK**						
**50**	8·81	-422,116	-47,913 (dominates)	52·86	527,646	9,982
**100**	8·81	77,884	8,840	52·86	1,027,646	19,441
**USA**						
**100**	8·81	-1,718,475	-195,058 (dominates)	52·86	536,702	10,153
**200**	8·81	-218,475	-24,798 (dominates)	52·86	2,036,702	38,530

ΔQALY, ΔCost: difference is QALYs/Costs (with serum spectroscopy test – without test), 10K patients
ICER: Incremental cost-effectiveness ratio

Incremental cost-effectiveness ratios were well below standard threshold values of £20,000 to £30,000 per quality adjusted life year (QALY) gained used in the UK, and similar thresholds used internationally, provided the test cost did not exceed £100. The base case results demonstrate that the serum spectroscopy test dominates (more effective and less costly) standard care at the lower bound of test cost in the primary care setting in both the UK and USA. At the upper bound of test cost the ICERs may be within commonly used thresholds or, in the case of the USA, remain dominant to standard care. In the secondary care setting ICERs of £9,982 and $10,153 at the lower bounds of test cost indicate that this test is potentially cost-effective in this setting. At the upper bounds ICERs may still be within commonly used thresholds for cost-effectiveness.

Sensitivity Analysis Results

The performance of the test with regards to levels of sensitivity and specificity are addressed using sensitivity analysis. OWSA results for a range of test specificities are displayed in Figure 5 and 6, for primary and secondary care respectively, displaying the ICER with

For peer review only

varying test specificity. Note that the estimated QALYs do not change with specificity in the model therefore changes in the ICER are due solely to changes in incremental costs. Varying sensitivity changes both estimated QALYs and estimated costs therefore results of the OWSA for test sensitivity are presented on the cost-effectiveness plane (Appendix 5). In primary care, using the upper cost limit of £100 it is evident that the test is deemed cost effective at specificities of around 0·9 and above, where the ICER is below standard thresholds. In contrast, at the lower cost limit, the test is cost effective at specificities above 0·8. Although the serum spectroscopy test is not cost saving at low cost (or near perfect specificities), the test is still considered cost effective at specificity levels around 0·7 and 0·8 for £50 and £100 pricing respectively.

The OWSA results highlight how ICERs in scenario 2 (secondary care) are strongly influenced by test sensitivity while ICERs in scenario 1 (primary care) are more strongly influenced by test specificity. These features are a result of the varying prevalence of disease and the assumption in scenario 2 that 100% of patients are referred for imaging following negative result. It should be noted that test specificity is important in both scenarios. Relatively small improvements in test specificity can substantially change the ICER, while larger improvements in test sensitivity are required to substantially alter the ICERs.

Additional scenario analyses are also reported in Appendix 5. These demonstrate that results are robust to using mean survival estimates rather than median survival estimates and including additional consultation costs for positive test results. If the prevalence of brain tumours in Scenario 1 is 1% rather than 0·5% the incremental QALYs increase substantially and the ICERs are reduced.

The PSA results reported in Figure 7 and 8 indicate that at a test cost of £50 and an ICER threshold of £30,000 per QALY there is a near 100% probability the serum spectroscopy test is cost-effective in scenario 1 and approximately 90% probability it is cost-effective in scenario 2. The corresponding probabilities at the upper bound cost of £100 are approximately 85% and 75%.

For peer review only

Discussion

This economic evaluation establishes the potential for serum spectroscopy to have a role in the diagnosis of both benign and malignant brain tumours in both primary and secondary care. The potential costs and health benefits of testing using a spectroscopic method prior to CT/MRI tests (or in some scenarios to avoid imaging) have been estimated based on a mathematical model with parameter values taken from published studies and expert opinion. This diagnostic tool is sensitive to all brain tumours (benign or malignant), however, this assessment is closely aligned with the diagnosis of primary gliomas, where there is a maximum potential benefit to the health service.

The major limitations of this analysis relate to the use of proof-of-concept studies and a disease natural history model rather than direct clinical trial evidence. This creates additional uncertainties. Results should be interpreted as indicative and used primarily to guide future evidence generation. Furthermore, the scenarios explored were limited in scope; future studies should continue to refine understanding of the role of the test in real-world clinical decision making.

When used as a triage tool in primary care, this novel test has the potential to deliver improvements in health outcomes and also to reduce costs. At the lower end of test costs, the technology would be cost-saving for the health service. At higher test costs the technology is still likely to be considered cost-effective in HTA agency decision processes.

In Scenario 2, in which serum spectroscopy is used as a triage tool in secondary care, the technology will create additional costs but also produce sizable health benefits. At test costs of under £100 ($200) the technology would be likely to be considered as a cost-effective use of resources in HTA agency decision processes in the UK (and USA). It is assumed that in both scenarios, the uptake of the test in the USA would mirror that of the UK; however, this would need to be explored further, alongside clinical experts of the USA care pathways.

Sensitivity analyses have demonstrated the importance of diagnostic performance on the cost-effectiveness of the test. In particular, test specificity is important in the primary care setting. If test specificity is 87·5% or worse the technology may not be considered cost-effective at higher values of assumed test cost. This is due to the increased number of false positive results in this low prevalence population, generating a greatly increased proportion of fast-track imaging studies which increases costs.

To strengthen the case that this approach represents a cost-effective use of healthcare resources it is necessary to establish the diagnostic performance of the test prospectively. This can be accomplished by a suitably large cohort study in which serum spectroscopy is used alongside current clinical practice in one of the patient groups included in this evaluation. It would be appropriate to initially target the secondary care patient population, because the higher prevalence of disease in this group will reduce the sample size needed to accurately estimate diagnostic performance.

For peer review only

Decision makers are often most interested in patient outcomes, such as survival, rather than intermediate outcomes, such as accuracy or speed of diagnosis (although this latter point is vital for treatment of high grade gliomas). From this perspective, a randomized trial, or a prospective cohort study with extended follow-up, may be required to fully establish the size of survival and quality-of-life benefits of including a serum spectroscopy test in the diagnostic pathway. A trial with primary outcomes relating to survival and quality-of-life would be specific to either the primary or secondary care setting (rather than generalisable to both), would need a large sample size, and would also require a follow-up period to capture survival benefits. In the case of malignant glioma this would require a period of at least 24 months. Such a trial would clearly be expensive, time consuming and may be unfeasible. Decision makers may be willing to make a decision on implementation of the blood test based on the modeled effects of improvements in intermediate outcomes on later patient outcomes. In this situation, the model proposed in this evaluation, populated with diagnostic performance and other data from a prospective trial, could be used to inform decisions about the wider adoption of the technology.

Future developments beyond trials such as emerging epidemiological evidence and new technologies should also be included in any future evaluations. It was not possible to foresee and include all such possible scenarios in this early evaluation but that should not preclude assessment in the light of new evidence. Updated analysis should inform any decisions about system wide implementation.

Several results in this analysis suggest cost savings through reduced use of imaging for patients with a negative test result. To make the case that a serum spectroscopic test can improve the efficiency of the diagnostic pathway prospective studies will also need to explore the impact of these test results on clinician and patient imaging study decisions. The possibility remains that the test may triage patients, but may not reduce the number of scans being conducted, and could potentially increase the demand on imaging. However, this triaging of patients would still benefit each patient that is provided with an early diagnosis.

This evaluation has explored the potential for serum spectroscopy to be a cost-effective addition to the diagnostic pathway for brain tumours. It has demonstrated that in specific scenarios this novel test may be an effective and cost-effective technology in reducing the delay to diagnosis for patients with brain tumours. Prospective trials are required to provide definitive evidence.

For peer review only

References

1 Burnet, N., Jefferies, S., Benson, R., Hunt, D. & Treasure, F. Years of life lost (YLL) from cancer is an important measure of population burden—and should be considered when allocating research funds. *Brit J Cancer* 2005; **92**: 241–5.

2 Hands, J. R., Clemens, G., Stables, R. *et al.* Brain tumour differentiation: rapid stratified serum diagnostics via attenuated total reflection Fourier-transform infrared spectroscopy. *J Neurooncol* 2016; **127**: 463–72.

3 Smith, B. R., Ashton, K. M., Brodbelt, A. *et al.* Combining random forest and 2D correlation analysis to identify serum spectral signatures for neuro-oncology. *Analyst* 2016; **141**: 3668–78.

4 Finding a better way? Improving the quality of life for people affected by brain tumours, Report Published by The Brain Tumour Charity, 2014.

5 Aggarwal, A., Herz, N., Campbell, P. *et al.* Diagnostic delay and survival in high-grade gliomas - evidence of the 'waiting time paradox'? *Br J Neurosurg* 2015; **29**: 520–3.

6 Zhou, Y., Abel, G. A., Hamilton, W. *et al.* Diagnosis of cancer as an emergency: a critical review of current evidence. *Nat Rev Clin Oncol* 2017; **14**: 45–56.

7 McPhail, S., Elliss-Brookes, L., Shelton, J. *et al.* Emergency presentation of cancer and short-term mortality. *Brit J Cancer* 2013; **109**: 2027–34.

8 Blanks, R., Moss, S., McGahan, C., Quinn, M. & Babb, P. Effect of NHS breast screening programme on mortality from breast cancer in England and Wales, 1990-8: comparison of observed with predicted mortality. *BMJ* 2000; **321**: 665–9.

9 UK Colorectal Cancer Screening Pilot Group. Results of the first round of a demonstration pilot of screening for colorectal cancer in the United Kingdom. *BMJ* 2004; **329**: 133.

10 Quinn, M., Babb, P., Jones, J. & Allen, E. Papers Effect of screening on incidence of and mortality from cancer of cervix in England: evaluation based on routinely collected statistics. *BMJ* 1999; **318**: 904.

11 Frankel, S., Smith, G. D., Donovan, J. & Neal, D. Screening for prostate cancer. *The Lancet* 2003; **361**: 1122–8.

12 Gadalla, S., Pfeiffer, R., Kristinsson, S. *et al.* Brain tumors in patients with myotonic dystrophy: a population□based study. *Eur J Neurol* 2016; **23**: 542–47.

13 Callaghan, B. C., Kerber, K. A., Pace, R. J., Skolarus, L. E. & Burke, J. F. Headaches and neuroimaging: high utilization and costs despite guidelines. *JAMA Intern Med* 2014; **174**: 819–21.

14 National Institute for Health and Care Excellence, 2015. Suspected cancer: recognition and referral. NICE Guideline (MG12).

15 Livermore, L. J., Ma, R., Bojanic, S. & Pereira, E. A. Yield and complications of frame-based and frameless stereotactic brain biopsy–the value of intra-operative histological analysis. *Brit J Neurosurg* 2014; **28**: 637–44.

16 Weber, M. A., Zoubaa, S., Schlieter, M. *et al.* Diagnostic performance of spectroscopic and perfusion MRI for distinction of brain tumors. *Cancer Imaging* 2006; **6**: S32–S41.

17 Baker, M. J., Trevisan, J., Bassan, P. *et al.* Using Fourier transform IR spectroscopy to analyze biological materials. *Nature Protoc* 2014; **9**: 1771–91.

18 Lovergne, L., Bouzy, P., Untereiner, V. *et al.* Biofluid infrared spectro-diagnostics: pre-analytical considerations for clinical applications. *Faraday Discuss* 2016; **187**: 521–37.

For peer review only

19 Baker, M. J., Hussain, S. R., Lovergne, L. *et al.* Developing and understanding biofluid vibrational spectroscopy: a critical review. *Chem Soc Rev* 2016; **45**: 1803–18.

20 Hochberg, F. H., Atai, N. A., Gonda, D. *et al.* Glioma diagnostics and biomarkers: an ongoing challenge in the field of medicine and science. *Expert Rev Mol Diagn* 2014; **14**: 439–52.

21 Hands, J. R., Abel, P., Ashton, K. *et al.* Investigating the rapid diagnosis of gliomas from serum samples using infrared spectroscopy and cytokine and angiogenesis factors. *Anal Bioanal Chem* 2013; **405**: 7347–55.

22 Wang, H. Z., Simonson, T. M., Greco, W. R. & Yuh, W. T. C. Brain MR Imaging in the Evaluation of Chronic Headache in Patients without Other Neurologic Symptoms. *Acad Radiol* 2001; **8**: 405–8.

23 Sempere, A. P., Porta-Etessam, J., Medrano, V. *et al.* Neuroimaging in the evaluation of patients with non-acute headache. *Cephalalgia* 2005; **25**: 30–5.

24 Clarke, C. E., Edwards, J., Nicholl, D. J. & Sivaguru, A. Imaging results in a consecutive series of 530 new patients in the Birmingham Headache Service. *J Neurol* 2010; **257**: 1274–9.

25 Kernick, D., Stapley, S., Goadsby, P. J. & Hamilton, W. What happens to new-onset headache presented to primary care? A case-cohort study using electronic primary care records. *Cephalalgia* 2008; **28**: 1188–95.

26 Hamilton, W. & Kernick, D. Clinical features of primary brain tumours: a case–control study using electronic primary care records. *Brit J Gen Pract* 2007; **57**: 695–9.

27 Do, V., Gebski, V. & Barton, M. The effect of wating for radiotherapy for grade II/IV gliomas. *Radiother Oncol* 2000; **57**: 131–6.

28 Burnet, N., Jefferies, S., Benson, R., Hunt, D. & Treasure, F. Years of life lost (YLL) from cancer is an important measure of population burden—and should be considered when allocating research funds. *Brit J Cancer* 2005; **92**: 241–45.

29 Garside, R., Pitt, M., Anderson, R. *et al.* The effectiveness and cost-effectiveness of carmustine implants and temozolomide for the treatment of newly diagnosed high-grade glioma: a systematic review and economic evaluation. *Health Technology Assessment* 2007; **11**.

30 National Institute for Health and Care Excellence, 2013. Guide to the methods of technology appraisal. NICE Guideline (PMG9).

31 Simpson, G. C., Forbes, K., Teasdale, E., Tyagi, A. & Santosh, C. Impact of GP direct-access computerised tomography for the investigation of chronic daily headache. *Br J Gen Pract* 2010; **60**: 897–901.

For peer review only

FIGURE LEGENDS

Figure 1. The diagnostic pathway of brain tumours. Timings relate to the diagnosis of high grade gliomas and are based upon discussion with the Clinical Focus Team and Aggarwal et al. (2015)[5]. (*OPD – Outpatient department; MDT – Multidisciplinary team*)

Figure 2. Attenuated total reflectance (ATR)-Fourier transform Infrared (FTIR) spectroscopic test of blood serum for the diagnosis and stratification of brain tumours using machine learning algorithms.

Figure 3. An unprocessed spectrum derived from human blood serum using ATR-FTIR spectroscopy. Spectral regions correspond to known bond vibrations and can therefore be associated with groups of biomolecules such as protein, lipid, phosphate and carbohydrates. Broad examples of blood serum constituents are listed.

Figure 4. A decision tree model describing the integration of a serum spectroscopy test in the current diagnostic pathway, and the effect on MRI/CT imaging for suspected brain tumour.

Figure 5 – Incremental cost effectiveness ratios (ICERs) at various specificities in primary care (‘Scenario 1’). The £30,000 ICER threshold is displayed as a dashed horizontal line.

Figure 6 - Incremental cost effectiveness ratios (ICERs) various specificities in Secondary Care (‘Scenario 2’). The £30,000 ICER threshold is displayed as a dashed horizontal line.

Figure 7 – Cost effectiveness acceptability curve (CEAC) at £50 and £100 per test – Primary care (‘Scenario 1’)

Figure 8 – Cost effectiveness acceptability curve (CEAC) at £50 and £100 per test – Secondary care (“Scenario 2”).

For peer review only

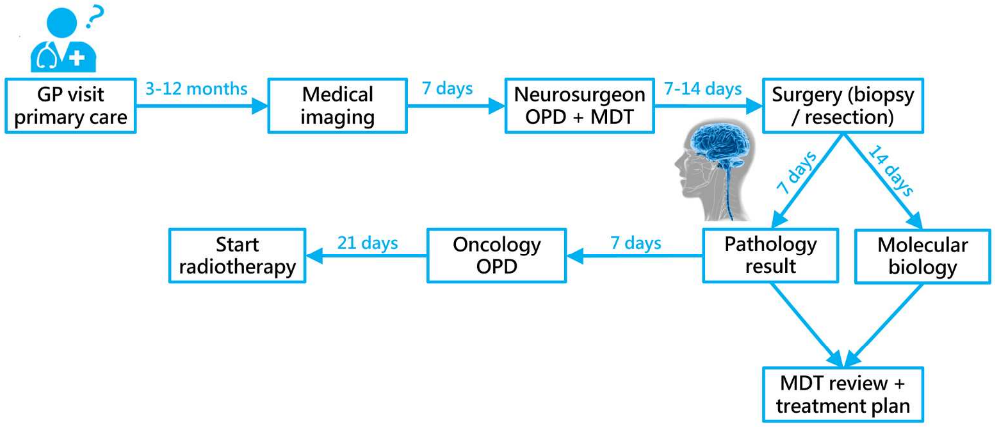

Figure 1. The diagnostic pathway of brain tumours. Timings relate to the diagnosis of high grade gliomas and are based upon discussion with the Clinical Focus Team and Aggarwal et al. (2015)5. (OPD – Outpatient department; MDT – Multidisciplinary team)

169x77mm (300 x 300 DPI)

For peer review only

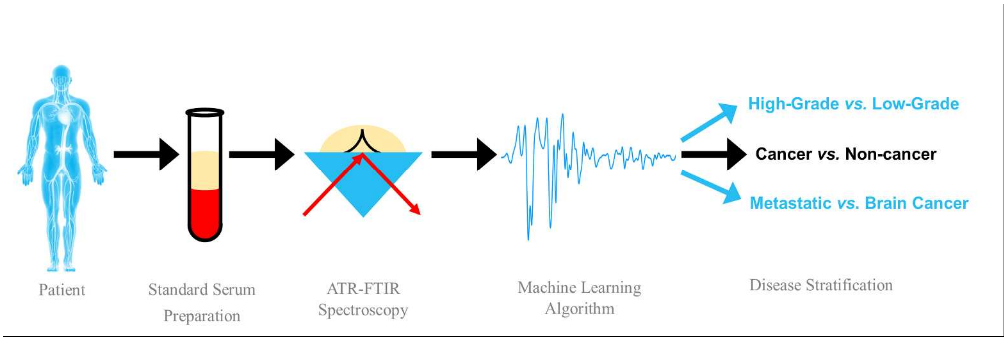

Figure 2. Attenuated total reflectance (ATR)-Fourier transform Infrared (FTIR) spectroscopic test of blood serum for the diagnosis and stratification of brain tumours using machine learning algorithms.

210x70mm (300 x 300 DPI)

peer review only

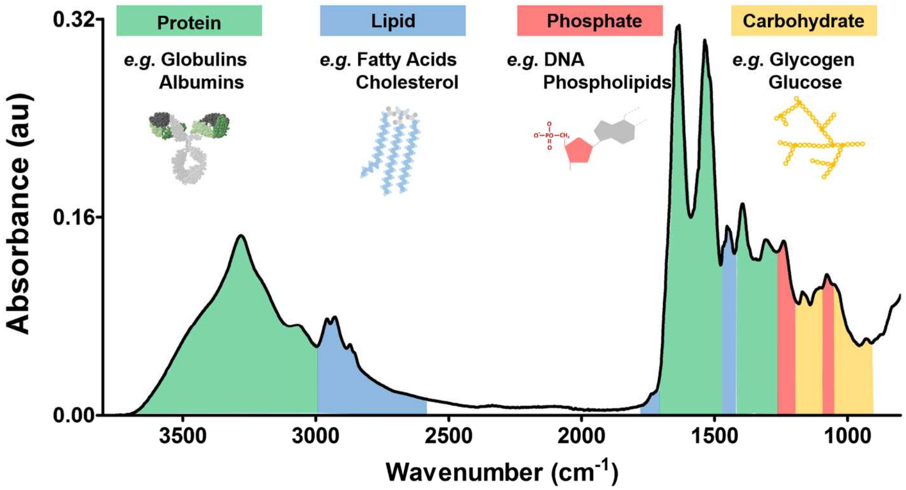

Figure 3. An unprocessed spectrum derived from human blood serum using ATR-FTIR spectroscopy. Spectral regions correspond to known bond vibrations and can therefore be associated with groups of biomolecules such as protein, lipid, phosphate and carbohydrates. Broad examples of blood serum constituents are listed.

169x95mm (300 x 300 DPI)

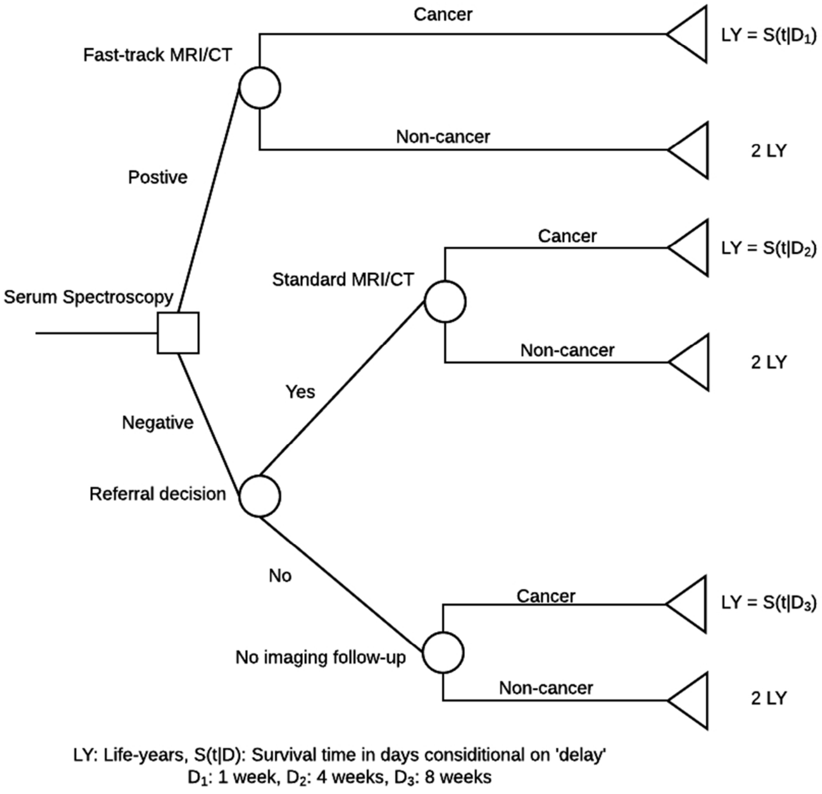

Figure 4. A decision tree model describing the integration of a serum spectroscopy test in the current diagnostic pathway, and the effect on MRI/CT imaging for suspected brain tumour.

82x74mm (300 x 300 DPI)

For peer review only - http://bmjopen.bmj.com/site/about/guidelines.xhtml

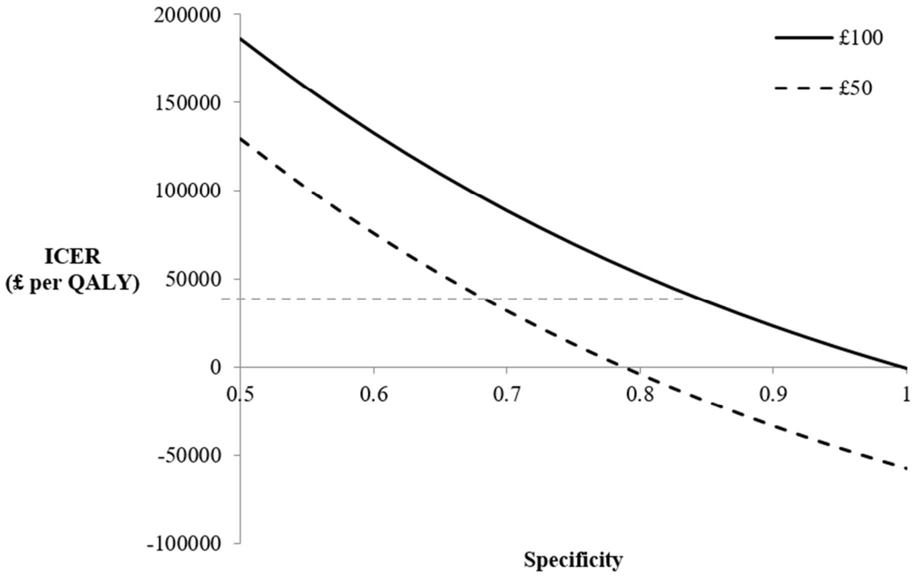

Figure 5 – Incremental cost effectiveness ratios (ICERs) at various specificities in primary care (‘Scenario 1’). The £30,000 ICER threshold is displayed as a dashed horizontal line.

78x49mm (300 x 300 DPI)

view only

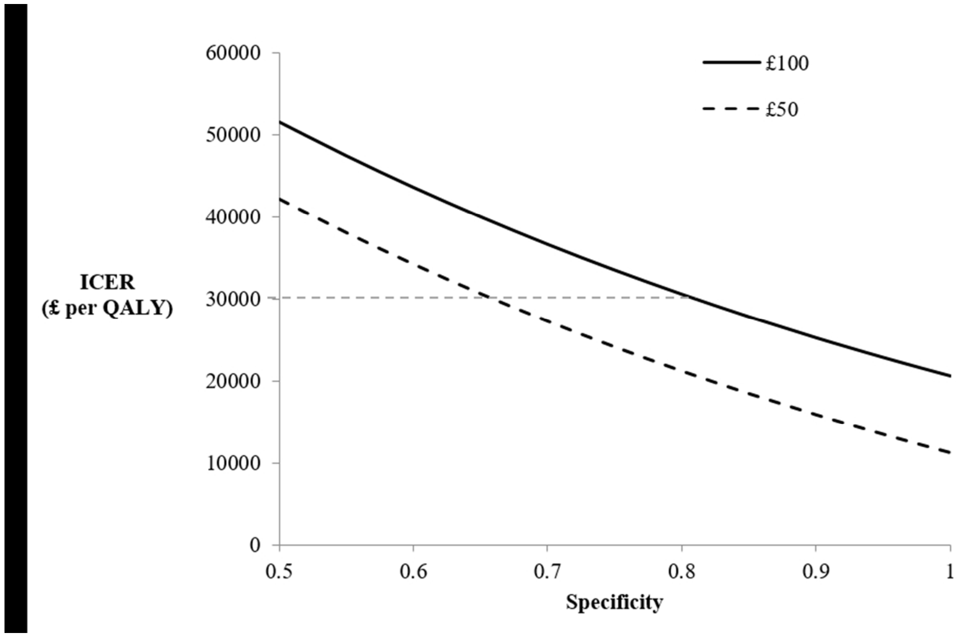

Figure 6 - Incremental cost effectiveness ratios (ICERs) various specificities in Secondary Care ('Scenario 2'). The £30,000 ICER threshold is displayed as a dashed horizontal line.

80x50mm (300 x 300 DPI)

view only

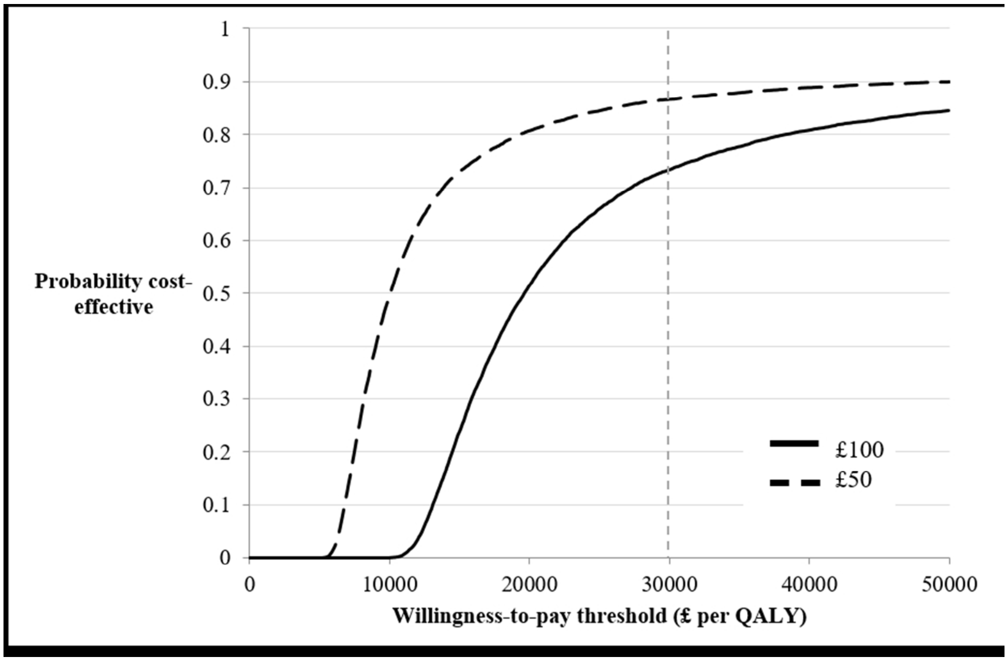

Figure 7 – Cost effectiveness acceptability curve (CEAC) at £50 and £100 per test – Primary care ('Scenario 1')

78x51mm (300 x 300 DPI)

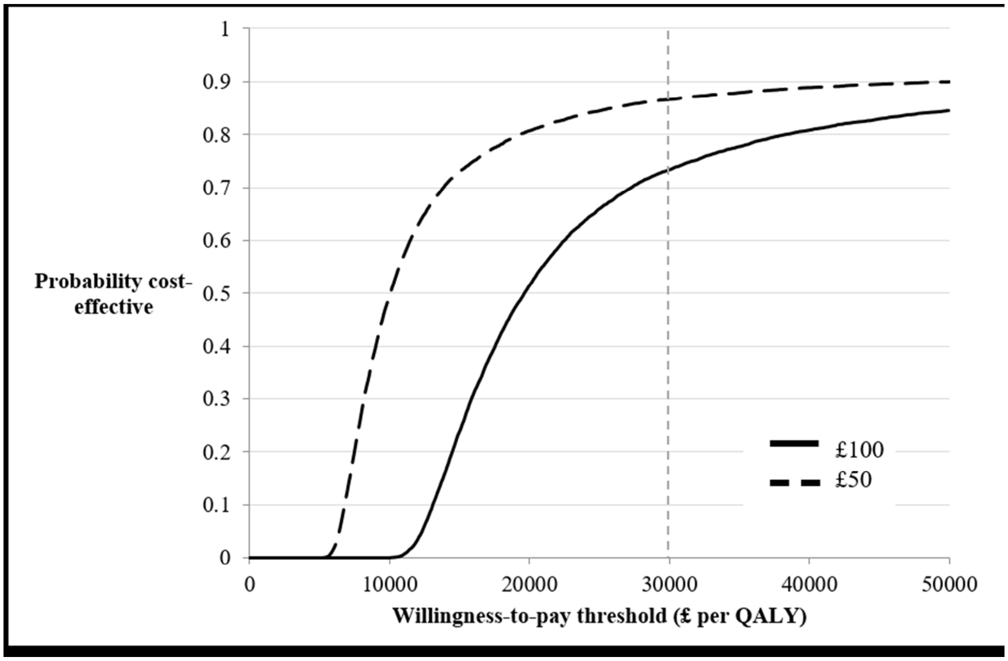

Figure 8 – Cost effectiveness acceptability curve (CEAC) at £50 and £100 per test – Secondary care ("Scenario 2").

78x51mm (300 x 300 DPI)

SUPPLEMENTARY MATERIAL

Appendix 1 – Clinical expert group

Clinical expert group participants: Mr Michael D Jenkinson (Walton Centre NHS Trust & University of Liverpool), Dr Paul Brennan (University of Edinburgh), Prof. Anthony Chalmers (University of Glasgow & Beatson Centre), Prof. Willie Hamilton (University of Exeter Medical School), Mr Kevin O'Neill (Imperial College Healthcare NHS Trust), Dr Allan Janes (Beatson West of Scotland Cancer Centre), Dr Babar Vaqas (Imperial College Healthcare NHS Trust), Dr David Kernick (St Thomas Health Centre, Exeter), Dr James Livermore (University of Oxford), Dr Ruth Board (Lancashire Teaching Hospitals NHS Trust), Prof. Timothy Dawson (Lancashire Teaching Hospitals NHS Trust), Miss Elvira Lekka (Lancashire Teaching Hospitals NHS Trust), Mr John Goodden (Leeds Teaching Hospital NHS Trust) and Dr. Samantha Mills (Walton Centre NHS Trust)

For peer review only

Appendix 2 – Decision tree model

The decision node probabilities that populate the decision tree model are reported in Table S1. The model is simplified by assuming that the reference test (MRI/CT) has perfect accuracy (100% sensitivity and 100% specificity). This is desirable if we wish to evaluate the test against an MRI/CT only diagnostic pathway in which these imaging studies are considered as the 'gold standard'. It is also difficult to obtain valid estimates of MRI/CT sensitivity and specificity for this indication as these are not reported in a comparable manner in the literature. This simplification will not substantially alter the economic results compared to using more realistic values for the scenarios under consideration. This is because it is assumed confirmation of the diagnosis always requires imaging and therefore outcomes for patients that would hypothetically be classified correctly using this test but incorrectly by MRI/CT will remain the same whether or not the test is used. The effect of less than perfect accuracy of the reference test is therefore the same as a (small) decrease in prevalence as a proportion of patients with the disease cannot benefit from the additional test due to incorrect subsequent testing. Outcomes are calculated by 'rolling-back' the tree for both cancer cases and non-cases and taking an average of the two groups weighted by the prevalence of brain tumours in the scenario population.

Table S1. Decision node probabilities for decision tree model describing the integration of a serum spectroscopy test in the current diagnostic pathway

**Cancer cases:**	**Probability to upper branch**	**Probability to lower branch**
Serum Spectroscopy Test (test sensitivity)	0·928	0·072
Standard MRI/CT (reference test sensitivity)	1	0
Fast-track MRI/CT (reference test sensitivity)	1	0
Referral decision	S1:0·5, S2: 1	S1:0·5, S2: 0
Non-cases:		
Serum Spectroscopy Test (test specificity)	0·085	0·915
Standard MRI/CT (reference test specificity)	0	1
Fast-track MRI/CT (reference test specificity)	0	1
Referral decision	S1:0·5, S2: 1	S1:0·5, S2: 0

S1 & S2: Scenarios 1 &2

For peer review only

Appendix 3 – Assumptions and details of cost effective analysis model

Effect of testing on time-to-diagnosis and time-to-treatment
To estimate the expected time-to-diagnosis under a fast-track pathway it is assumed that this would match the current median time-to-diagnosis for patients presenting with brain tumour in emergency care as observed in Aggarwal et al [5]. This study reported a cohort of high grade glioma patients from a single hospital in London, UK. No other suitable estimates of time-to-treatment or time-to-diagnosis were identified in the literature. This is the most common primary brain tumour in the indications considered in this evaluation. Median time-to-diagnosis for the standard pathway is also taken from the same source.

Effect of testing on use of imaging studies
The effect of spectroscopic testing on imaging study decisions of clinicians and patients is uncertain. Based on clinical expert input it was assumed, in the base case, that in secondary care all patients would continue to imaging, while in primary care 50% would continue to imaging following a negative spectroscopy result.

Effect of early diagnosis on patient outcomes
A systematic review of the literature failed to identify any studies that directly estimated the effect of earlier (or later) diagnosis on outcomes for primary brain tumours. Instead, based clinical expert guidance, estimates of the effects of time-to-treatment were sourced from the literature. The estimated effects are based on fitting natural history models to observational datasets of high grade glioma patients.[27,28] It is necessary to use a natural history model to produce survival estimates rather than using the survival data stratified by time-to-treatment directly, in order to adjust for potential confounding factors influencing both time-to-treatment and survival.

A one-to-one correspondence is assumed for the effects of an additional day added to the time-to-diagnosis and an additional day added to the time-to-treatment. Median survival by weeks between diagnosis and treatment are displayed in Table 2. The hazard ratio per additional day delay calculated in Do et al of 1·015 was applied to each day between diagnosis and treatment up to 28 days to calculate median survival time. Time between diagnosis and treatment beyond 28 days were fixed at the same median survival as 28 days.

Table S2. Relationship between time-to-diagnosis and survival

Time-to-diagnosis (weeks)	Median survival (weeks)
0	46
1	41·4
2	37·3
3	33·6
4	30·3

Source: Do et al 2000

Resource use and cost

It is assumed that there is no additional cost for converting a small proportion of standard referral to additional urgent referrals. This could be justified on the basis that patients receiving negative results allows more flexibility in the scheduling for these patients. This could create more opportunity to schedule patients with positive spectroscopy results sooner. As a larger proportion of patients are referred for immediate imaging it was assumed costs would increase because achieving this would require additional capacity. It was assumed that unit costs for MRI and CT grow exponentially with increasing proportion of immediate referrals such that costs double if 50% of patients are referred for immediate imaging.

Treatment costs are assumed to be the same for both fast-track and standard referrals that are diagnosed as cases. This may be a conservative assumption as treatment costs are likely to increase as disease progresses and therefore earlier treatment may, on average, be less costly than later treatment. However, it is also possible that

For peer review only

treatment costs could be increased if some cases are converted from palliative care to more aggressive treatment. The balance of these effects is unknown given the lack of appropriate data to estimate the effects. Secondary treatment and end-of-life costs are similarly assumed to be equal between fast track and standard referrals. This reflects the model assumption that all patients ultimately progress.

The time intervals considered (0-4 weeks) are too short for discounting to have an important impact on cost estimates therefore the effects of moving forward treatment on present value is not considered.

For peer review only

Appendix 4 - Total demand for serum spectroscopy tests in UK in two clinical scenarios

1. Primary care

An assumption that patients currently referred in direct-access imaging service would be selected for spectroscopic testing can provide a conservative estimate of the total number of patients likely to be tested. Three sources from the literature were considered:

1. A direct-access brain MRI service in Nottingham (UK) reported 130 patients per quarter (520 per year) among a population of 342,000.[32,33] The population of the UK is approximately 61 million therefore, assuming Nottingham is representative of the UK general population demand for brain MRI, there would be approximately 93,000 per year in the UK.
2. A direct access CT service for chronic headache in Glasgow (UK) reported 4404 patients referred over 8 years (551 per year) among a population of approximately 1 million.[31] Assuming this was representative of the UK generally then the demand for this type of service would be approximately 34,000 for the UK.
3. A direct access CT neuroimaging service in Lothian (UK) reported 389 exams per year for a population of approximately 442,000.[34] Assuming this was representative of the UK generally then the demand for this type of service would be approximately 54,000 for the UK.

The Glasgow estimate is particularly low and may result from limited uptake in the early part of the 8 year period for which data were reported. As it was not possible to determine if uptake increased over the period or was stable this estimate was not considered reliable. The Nottingham and Lothian estimates report potentially more stable and established services and are therefore more likely to reliably estimate demand. The likely demand for this novel test, if the indication is considered identical to neuroimaging, is therefore between 54,000 and 93,000. This is likely to be conservative as the test may well have a slightly broader indication than current neuroimaging. A reasonable point estimate may be approximately 75,000.

2. Secondary Care

An estimate of the total demand for serum spectroscopic tests in the UK can be made based on the reported incidence of brain tumour in the UK and the proportion of cases currently diagnosed through secondary (non-emergency) care. Annual incidence of malignant brain tumours in the UK is approximately 4,700.[35] Approximately one third of these are likely to present through secondary, non-emergency care. Using the positive predictive value for patients currently referred to neuroimaging in this setting of 3% suggests that for each case there will be 33 non-case patients receiving neuroimaging. In total, in the UK, this would imply 53,000 patients per year in secondary care may be suitable for spectroscopic testing.

For peer review only

Appendix 5 - Sensitivity Analysis

One-way sensitivity analysis (OWSA)

OWSA results for a range of test sensitivity are displayed in S1 and S2 as a series of points on a cost-effectiveness (CE) plane with incremental QALYs (intervention-control) on the x-axis and incremental costs (intervention - control) on the y-axis. Corresponding OWSA results for a range of test specificities in primary and secondary care are shown in Figure 3 and 4 in the main article text respectively. These are displayed with the ICER on the y-axis and the test specificity on the x-axis. Note that the estimated QALYs do not change with specificity in the model therefore changes in the ICER are due solely to changes in incremental costs.**Error! Reference source not found.** All OWSA analyses show results assuming the spectroscopy based test costs of £50 and £100.

Figure S1 – Results on CE plane over a range of test sensitivities (0·5-0·99) – Primary care

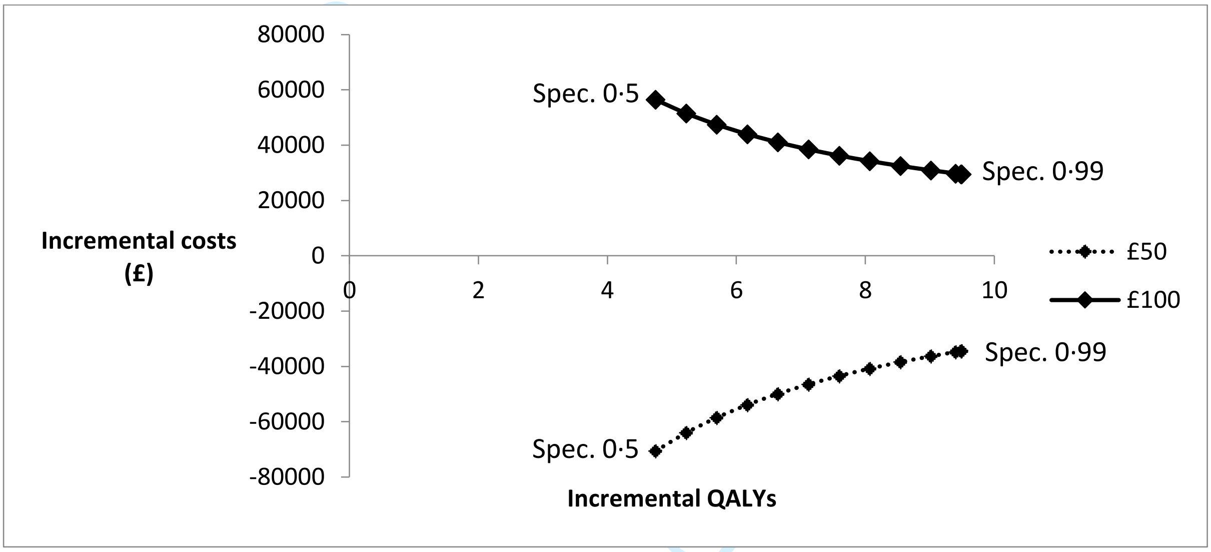

Figure S2 - Results on CE plane over a range of test sensitivities (0·5-0·99) – Secondary care

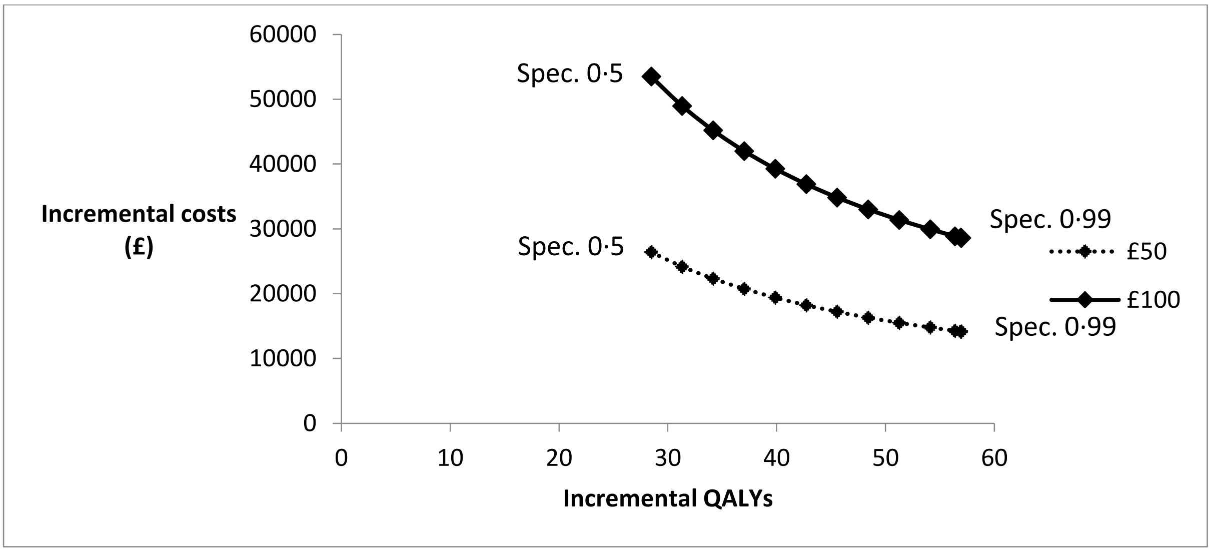

Scenario analysis

Table S3 – Scenario analysis: Mean survival estimates

	Scenario 1			Scenario 2		
Serum spectroscopy test cost (£)	ΔQALY	ΔCost	ICER	ΔQALY	ΔCost	ICER
50	12·83	-212808	-16582 (dominates)	77	540273	7016
100	12·83	287192	22377	77	1040273	13509

Table S4 - Additional costs scenario analysis

	Scenario 1			Scenario 2		
Serum spectroscopy test cost (£)	ΔQALY	ΔCost	ICER	ΔQALY	ΔCost	ICER
50	8·81	-379962	-43128 (dominates)	52·86	566247	10712
100	8·81	120038	13625	52·86	1066247	20171

Table S5 - Alternative prevalence estimates (1% prevalence) in primary care setting (Scenario 1)

	Scenario 1		
Serum spectroscopy test cost (£)	ΔQALY	ΔCost	ICER
50	17·62	-413220	-23452 (dominates)
100	17·62	86780	4925

Table S6– Alternative proportion of test negative cases referred to imaging (Scenario 1 only)

		Scenario 1		
Serum spectroscopy test cost (£)	% of test negative cases referred	ΔQALY	ΔCost	ICER
50	50 (base)	8·81	-422,116	-47,913
	75	17·62	-131,050	-7,438
	90	17·62	38,252	2,171
	100	17·62	151,120	8,577
100	50 (base)	8·81	77,884	8,840
	75	17·62	368,950	20,939
	90	17·62	538,252	30,548
	100	17·62	651,120	36,953

For peer review only

References

1 Burnet, N., Jefferies, S., Benson, R., Hunt, D. & Treasure, F. Years of life lost (YLL) from cancer is an important measure of population burden—and should be considered when allocating research funds. *Brit J Cancer* 2005; **92**: 241–5.

2 Hands, J. R., Clemens, G., Stables, R. *et al.* Brain tumour differentiation: rapid stratified serum diagnostics via attenuated total reflection Fourier-transform infrared spectroscopy. *J Neurooncol* 2016; **127**: 463–72.

3 Smith, B. R., Ashton, K. M., Brodbelt, A. *et al.* Combining random forest and 2D correlation analysis to identify serum spectral signatures for neuro-oncology. *Analyst* 2016; **141**: 3668–78.

4 Finding a better way? Improving the quality of life for people affected by brain tumours, Report Published by The Brain Tumour Charity, 2014.

5 Aggarwal, A., Herz, N., Campbell, P. *et al.* Diagnostic delay and survival in high-grade gliomas - evidence of the 'waiting time paradox'? *Br J Neurosurg* 2015; **29**: 520–3.

6 Zhou, Y., Abel, G. A., Hamilton, W. *et al.* Diagnosis of cancer as an emergency: a critical review of current evidence. *Nat Rev Clin Oncol* 2017; **14**: 45–56.

7 McPhail, S., Elliss-Brookes, L., Shelton, J. *et al.* Emergency presentation of cancer and short-term mortality. *Brit J Cancer* 2013; **109**: 2027–34.

8 Blanks, R., Moss, S., McGahan, C., Quinn, M. & Babb, P. Effect of NHS breast screening programme on mortality from breast cancer in England and Wales, 1990-8: comparison of observed with predicted mortality. *BMJ* 2000; **321**: 665–9.

9 UK Colorectal Cancer Screening Pilot Group. Results of the first round of a demonstration pilot of screening for colorectal cancer in the United Kingdom. *BMJ* 2004; **329**: 133.

10 Quinn, M., Babb, P., Jones, J. & Allen, E. Papers Effect of screening on incidence of and mortality from cancer of cervix in England: evaluation based on routinely collected statistics. *BMJ* 1999; **318**: 904.

11 Frankel, S., Smith, G. D., Donovan, J. & Neal, D. Screening for prostate cancer. *The Lancet* 2003; **361**: 1122–8.

12 Gadalla, S., Pfeiffer, R., Kristinsson, S. *et al.* Brain tumors in patients with myotonic dystrophy: a population-based study. *Eur J Neurol* 2016; **23**: 542–47.

13 Callaghan, B. C., Kerber, K. A., Pace, R. J., Skolarus, L. E. & Burke, J. F. Headaches and neuroimaging: high utilization and costs despite guidelines. *JAMA Intern Med* 2014; **174**: 819–21.

14 National Institute for Health and Care Excellence, 2015. Suspected cancer: recognition and referral. NICE Guideline (MG12).

15 Livermore, L. J., Ma, R., Bojanic, S. & Pereira, E. A. Yield and complications of frame-based and frameless stereotactic brain biopsy–the value of intra-operative histological analysis. *Brit J Neurosurg* 2014; **28**: 637–44.

16 Weber, M. A., Zoubaa, S., Schlieter, M. *et al.* Diagnostic performance of spectroscopic and perfusion MRI for distinction of brain tumors. *Cancer Imaging* 2006; **6**: S32–S41.

17 Baker, M. J., Trevisan, J., Bassan, P. *et al.* Using Fourier transform IR spectroscopy to analyze biological materials. *Nature Protoc* 2014; **9**: 1771–91.

18 Lovergne, L., Bouzy, P., Untereiner, V. *et al.* Biofluid infrared spectro-diagnostics: pre-analytical considerations for clinical applications. *Faraday Discuss* 2016; **187**: 521–37.

For peer review only

19 Baker, M. J., Hussain, S. R., Lovergne, L. *et al.* Developing and understanding biofluid vibrational spectroscopy: a critical review. *Chem Soc Rev* 2016; **45**: 1803–18.
20 Hochberg, F. H., Atai, N. A., Gonda, D. *et al.* Glioma diagnostics and biomarkers: an ongoing challenge in the field of medicine and science. *Expert Rev Mol Diagn* 2014; **14**: 439–52.
21 Hands, J. R., Abel, P., Ashton, K. *et al.* Investigating the rapid diagnosis of gliomas from serum samples using infrared spectroscopy and cytokine and angiogenesis factors. *Anal Bioanal Chem* 2013; **405**: 7347–55.
22 Wang, H. Z., Simonson, T. M., Greco, W. R. & Yuh, W. T. C. Brain MR Imaging in the Evaluation of Chronic Headache in Patients without Other Neurologic Symptoms. *Acad Radiol* 2001; **8**: 405–8.
23 Sempere, A. P., Porta-Etessam, J., Medrano, V. *et al.* Neuroimaging in the evaluation of patients with non-acute headache. *Cephalalgia* 2005; **25**: 30–5.
24 Clarke, C. E., Edwards, J., Nicholl, D. J. & Sivaguru, A. Imaging results in a consecutive series of 530 new patients in the Birmingham Headache Service. *J Neurol* 2010; **257**: 1274–9.
25 Kernick, D., Stapley, S., Goadsby, P. J. & Hamilton, W. What happens to new-onset headache presented to primary care? A case-cohort study using electronic primary care records. *Cephalalgia* 2008; **28**: 1188–95.
26 Hamilton, W. & Kernick, D. Clinical features of primary brain tumours: a case–control study using electronic primary care records. *Brit J Gen Pract* 2007; **57**: 695–9.
27 Do, V., Gebski, V. & Barton, M. The effect of wating for radiotherapy for grade II/IV gliomas. *Radiother Oncol* 2000; **57**: 131–6.
28 Burnet, N., Jefferies, S., Benson, R., Hunt, D. & Treasure, F. Years of life lost (YLL) from cancer is an important measure of population burden—and should be considered when allocating research funds. *Brit J Cancer* 2005; **92**: 241–45.
29 Garside, R., Pitt, M., Anderson, R. *et al.* The effectiveness and cost-effectiveness of carmustine implants and temozolomide for the treatment of newly diagnosed high-grade glioma: a systematic review and economic evaluation. *Health Technology Assessment* 2007; **11**.
30 National Institute for Health and Care Excellence, 2013. Guide to the methods of technology appraisal. NICE Guideline (PMG9).
31 Simpson, G. C., Forbes, K., Teasdale, E., Tyagi, A. & Santosh, C. Impact of GP direct-access computerised tomography for the investigation of chronic daily headache. *Br J Gen Pract* 2010; **60**: 897–901.
32 Taylor, T., Evangelou, N., Porter, H., Hamilton, W. & Kernick, D. Headache: two views on the right approach in general practice. *Br J Gen Pract* 2014; **64**: 475–6.
33 Taylor, T. R., Evangelou, N., Porter, H. & Lenthall, R. Primary care direct access MRI for the investigation of chronic headache. *Clin Radiol* 2012; **67**: 24–7.
34 White, P., Halliday-Pegg, J. & Collie, D. Open access neuroimaging for general practitioners - diagnostic yield and influence on patient management. *Brit J Gen Pract* 2002; **52**: 33–35.
35 UK Statistics Authority. Cancer Registration Statistics, England, 2014. Office of National Statistics

For peer review only

CHEERS Checklist
Items to include when reporting economic evaluations of health interventions

The **ISPOR CHEERS Task Force Report**, *Consolidated Health Economic Evaluation Reporting Standards (CHEERS)—Explanation and Elaboration: A Report of the ISPOR Health Economic Evaluations Publication Guidelines Good Reporting Practices Task Force*, provides examples and further discussion of the 24-item CHEERS Checklist and the CHEERS Statement. It may be accessed via the *Value in Health* or via the ISPOR Health Economic Evaluation Publication Guidelines – CHEERS: Good Reporting Practices webpage: http://www.ispor.org/TaskForces/EconomicPubGuidelines.asp

Section/item	Item No	Recommendation	Reported on page No/ line No
**Title and abstract**			
Title	1	Identify the study as an economic evaluation or use more specific terms such as “cost-effectiveness analysis”, and describe the interventions compared.	1 / 1
Abstract	2	Provide a structured summary of objectives, perspective, setting, methods (including study design and inputs), results (including base case and uncertainty analyses), and conclusions.	3 / 1-29
**Introduction**			
Background and objectives	3	Provide an explicit statement of the broader context for the study.	5/2-12
		Present the study question and its relevance for health policy or practice decisions.	8/17-29
**Methods**			
Target population and subgroups	4	Describe characteristics of the base case population and subgroups analysed, including why they were chosen.	14/ 11-37
Setting and location	5	State relevant aspects of the system(s) in which the decision(s) need(s) to be made.	14/11-37
Study perspective	6	Describe the perspective of the study and relate this to the costs being evaluated.	26/3-32
Comparators	7	Describe the interventions or strategies being compared and state why they were chosen.	11-12/33-2
Time horizon	8	State the time horizon(s) over which costs and consequences are being evaluated and say why appropriate.	12/14-22
Discount rate	9	Report the choice of discount rate(s) used for costs and outcomes and say why appropriate.	12/2-3
Choice of health outcomes	10	Describe what outcomes were used as the measure(s) of benefit in the evaluation and their relevance for the type of analysis performed.	11/26-32
Measurement of effectiveness	11a	*Single study-based estimates:* Describe fully the design features of the single effectiveness study and why the single study was a sufficient source of clinical effectiveness data.	11/9-22

For peer review only

	11b	*Synthesis-based estimates:* Describe fully the methods used for identification of included studies and synthesis of clinical effectiveness data.	
Measurement and valuation of preference based outcomes	12	If applicable, describe the population and methods used to elicit preferences for outcomes.	9/1
Estimating resources and costs	13a	*Single study-based economic evaluation:* Describe approaches used to estimate resource use associated with the alternative interventions. Describe primary or secondary research methods for valuing each resource item in terms of its unit cost. Describe any adjustments made to approximate to opportunity costs.	11/33-40
	13b	*Model-based economic evaluation:* Describe approaches and data sources used to estimate resource use associated with model health states. Describe primary or secondary research methods for valuing each resource item in terms of its unit cost. Describe any adjustments made to approximate to opportunity costs.	12-13/4-4<br>24/1-17
Currency, price date, and conversion	14	Report the dates of the estimated resource quantities and unit costs. Describe methods for adjusting estimated unit costs to the year of reported costs if necessary. Describe methods for converting costs into a common currency base and the exchange rate.	11/38-40
Choice of model	15	Describe and give reasons for the specific type of decision-analytical model used. Providing a figure to show model structure is strongly recommended.	9/1
Assumptions	16	Describe all structural or other assumptions underpinning the decision-analytical model.	23-24/1-6
Analytical methods	17	Describe all analytical methods supporting the evaluation. This could include methods for dealing with skewed, missing, or censored data; extrapolation methods; methods for pooling data; approaches to validate or make adjustments (such as half cycle corrections) to a model; and methods for handling population heterogeneity and uncertainty.	12-13/4-4
**Results**			15/3-20
Study parameters	18	Report the values, ranges, references, and, if used, probability distributions for all parameters. Report reasons or sources for distributions used to represent uncertainty where appropriate. Providing a table to show the input values is strongly recommended.	
Incremental costs and outcomes	19	For each intervention, report mean values for the main categories of estimated costs and outcomes of interest, as well as mean differences between the comparator groups. If applicable, report incremental cost-effectiveness ratios.	15/3-20
Characterising uncertainty	20a	*Single study-based economic evaluation:* Describe the effects of sampling uncertainty for the estimated incremental cost and incremental effectiveness parameters, together with the impact	

For peer review only

		of methodological assumptions (such as discount rate, study perspective).	
	20b	*Model-based economic evaluation:* Describe the effects on the results of uncertainty for all input parameters, and uncertainty related to the structure of the model and assumptions.	17-18/2-2
Characterising heterogeneity	21	If applicable, report differences in costs, outcomes, or cost-effectiveness that can be explained by variations between subgroups of patients with different baseline characteristics or other observed variability in effects that are not reducible by more information.	
**Discussion**			
Study findings, limitations, generalisability, and current knowledge	22	Summarise key study findings and describe how they support the conclusions reached. Discuss limitations and the generalisability of the findings and how the findings fit with current knowledge.	19-20/4-20
**Other**			
Source of funding	23	Describe how the study was funded and the role of the funder in the identification, design, conduct, and reporting of the analysis. Describe other non-monetary sources of support.	2/28-33
Conflicts of interest	24	Describe any potential for conflict of interest of study contributors in accordance with journal policy. In the absence of a journal policy, we recommend authors comply with International Committee of Medical Journal Editors recommendations.	2/23-26

For consistency, the CHEERS Statement checklist format is based on the format of the CONSORT statement checklist

The **ISPOR CHEERS Task Force Report** provides examples and further discussion of the 24-item CHEERS Checklist and the CHEERS Statement. It may be accessed via the *Value in Health* link or via the ISPOR Health Economic Evaluation Publication Guidelines – CHEERS: Good Reporting Practices webpage: http://www.ispor.org/TaskForces/EconomicPubGuidelines.asp

The citation for the CHEERS Task Force Report is:
Husereau D, Drummond M, Petrou S, et al. Consolidated health economic evaluation reporting standards (CHEERS)—Explanation and elaboration: A report of the ISPOR health economic evaluations publication guidelines good reporting practices task force. Value Health 2013;16:231-50.

For peer review only

BMJ Open

Health economic evaluation of a serum based blood test for brain tumour diagnosis: Exploration of two clinical scenarios.

Journal:	*BMJ Open*
Manuscript ID	bmjopen-2017-017593.R3
Article Type:	Research
Date Submitted by the Author:	20-Apr-2018
Complete List of Authors:	Gray, Ewan; Health Improvement Scotland<br>Butler, Holly; Pure and Applied Chemistry; Technology & Innovation Building<br>Board, Ruth; Lancashire Teaching Hospitals NHS Foundation Trust, Rosemere Cancer Centre<br>Brennan, Paul; Western General Hospital, Department of Clinical Neurosciences<br>Chalmers, Anthony; University of Glasgow, Institute of Cancer Sciences; Beatson West of Scotland Cancer Centre<br>Dawson, Timothy; Lancashire Teaching Hospitals NHS Foundation Trust, Neurosurgery Department<br>Gooden, John; Leeds General Infirmary, Neurosurgery Department<br>Hamilton, Willie; University of Exeter Medical School, Primary Care Diagnostics<br>Hegarty, Mark; University of Strathclyde, Pure & Applied Chemistry<br>James, Allan; University of Glasgow Institute Of Molecular Cell And Systems Biology<br>Jenkinson, Michael; The Walton Centre NHS Foundation Trust<br>Kernick, David; St Thomas' Medical Group<br>Lekka, Elvira; Lancashire Teaching Hospitals NHS Foundation Trust, Neurosurgery Department<br>Livermore, Laurent; Oxford University Hospitals, NHS Foundation Trust, Department of Neurosurgery<br>Mills, Samantha; The Walton Centre NHS Foundation Trust<br>O'Neill , Kevin; Imperial College London<br>Palmer, David; University of Strathclyde, Pure & Applied Chemistry<br>Vaqas, Babar; Imperial College London, John Fulcher Neuro-Oncology Laboratory<br>Baker, Matthew; University of Strathclyde
**Primary Subject Heading**:	Health economics
Secondary Subject Heading:	Diagnostics, Health economics, Oncology
Keywords:	Biophysics < BASIC SCIENCES, HEALTH ECONOMICS, Neurology < INTERNAL MEDICINE, Neurological oncology < NEUROLOGY, Adult oncology < ONCOLOGY, Head & neck imaging < RADIOLOGY & IMAGING

For peer review only

Health economic evaluation of a serum based blood test for brain tumour diagnosis: Exploration of two clinical scenarios.

Ewan Gray[1†], Holly J. Butler[2,3†], Ruth Board[4], Paul M. Brennan[5], Anthony J Chalmers[6,7], Timothy Dawson[8], John Goodden[9], Willie Hamilton[10], Mark G. Hegarty[2,3], Allan James[11], Michael D. Jenkinson[12, 13], David Kernick[14], Elvira Lekka[8], Laurent J. Livermore[15], Samantha J. Mills[12], Kevin O'Neill[16], David S. Palmer[3,17], Babar Vaqas[16] Matthew J. Baker[2,3*]

[1] Health Improvement Scotland, NHS Scotland, Delta House, 50 West Nile Street, Glasgow, G1 2NP, UK; [2] WestCHEM, Department of Pure and Applied Chemistry, University of Strathclyde, Technology and Innovation Centre, 99 George Street, Glasgow, G1 1RD, UK; [3] ClinSpec Diagnostics Limited, Technology and Innovation Centre, 99 George St, G1 1RD, UK; [4] Rosemere Cancer Centre, Lancashire Teaching Hospitals NHS Trust, Royal Preston Hospital, Sharoe Green Lane, Preston, PR2 9HT, UK; [5] Department of Clinical Neurosciences, Western General Hospital, Edinburgh, EH4 2XU, UK; [6] Beatson West of Scotland Cancer Centre, 1053 Great Western Road, Glasgow, G12 0YN, UK; [7] Institute of Cancer Sciences, Wolfson Wohl Cancer Research Centre, University of Glasgow, Glasgow, G61 1QH; [8] Neurosurgery Department, Lancashire Teaching Hospitals NHS Trust, Royal Preston Hospital, Sharoe Green Lane, Preston, Lancashire, PR2 9HT, UK; [9] Neurosurgery Department, Leeds General Infirmary, Leeds, LS1 3EX, UK; [10] Primary Care Diagnostics, University of Exeter Medical School, College House, University of Exeter, Exeter, Devon, EX1 2LU, UK; [11] Institute of Molecular Cell and Systems Biology, Bower Building, Glasgow, G12 8QQ, UK; [12] The Walton Centre NHS Foundation Trust, Lower Lane, Fazakerley, Liverpool, L9 7LJ, UK; [13] Institute of Translational Medicine, Clinical Science Centre, University of Liverpool, Liverpool, L9 7LJ; [14] St Thomas' Medical Group, Cowick Street, Exeter, EX4 1HJ, UK; [15] Department of Neurosurgery, Oxford University Hospitals, NHS Foundation Trust, John Radcliffe Hospital, Oxford, OX3 9DU, UK; [16] John Fulcher Neuro-Oncology Laboratory, Imperial College, London, SW7 2AZ, UK; [17] WestCHEM, Department of Pure and Applied Chemistry, University of Strathclyde, Thomas Graham Building, 295 Cathedral Street, Glasgow, G1 1XL, UK

*Corresponding author: Dr Matthew J Baker, matthew.baker@strath.ac.uk, +44(0)141 548 4700, @ChemistryBaker

†These authors contributed equally to the paper

For peer review only

Copyright/Licence for publication

The Corresponding Author has the right to grant on behalf of all authors and does grant on behalf of all *authors,* a worldwide licence *to the Publishers and its licensees in perpetuity, in all forms, formats and media (whether known now or* created in the future), to i) publish, reproduce, distribute, display and store the Contribution, ii) translate the Contribution into other languages, create adaptations, reprints, include within collections and create summaries, extracts and/or, abstracts of the Contribution, iii) create any other derivative work(s) based on the Contribution, iv) to exploit all subsidiary rights in the Contribution, v) the inclusion of electronic links from the Contribution to third party material where-ever it may be located; and, vi) licence any third party to do any or all of the above

Authors' Contributions

EG conducted the health economic assessment including data analysis, data interpretation, and writing. HJB contributed to data interpretation, writing and providing details regarding clinical spectroscopy. MJB is the principal investigator of this research, and initiated this interdisciplinary project and team. RB, PMB, AJC, TD, JG, WH, AJ, MDJ, DK, EL, LJL, SJM, KO, and BV are members of the clinical focus group involved in spearheading translation; all of whom provided feedback on the manuscript. MGH, DSP, HJB and MJB are involved in the ClinSpec Diagnostics Limited project.

Competing Interest Declaration

MJB, MGH, HJB and DSP are all involved in ClinSpec Diagnostics Limited a prospective spin out company from the University of Strathclyde focusing on the translation of serum spectroscopic diagnostics (Company Number SC535447)

Role of the Funding Source

Scottish Enterprise is Scotland's main economic development agency and non-departmental public body of the Scottish Government. They work with partners in both the private and public sectors to exploit the best opportunities to deliver a significant, lasting effect on the Scottish economy. The High-Growth Spinout Programme helps researchers take their ideas and inventions from the lab to the global marketplace.

Acknowledgements

The authors acknowledge the support from Rosemere Cancer Foundation, Brain Tumour North West, EPSRC, and the Sydney Driscoll Neuroscience Foundation for funding.

For peer review only

Data Sharing Statement

No additional data available

For peer review only

Abstract

Objectives

To determine the potential costs and health benefits of a serum-based spectroscopic triage tool for brain tumours, that could be developed to reduce diagnostic delays in the current clinical pathway.

Design

A model-based health pre-trial economic assessment. Decision tree models were constructed based on simplified diagnostic pathways. Models were populated with parameters identified from rapid reviews of the literature and clinical expert opinion.

Setting

Explored as a test in both primary and secondary care (neuroimaging) in the UK health service, as well as application to the US.

Participants

Calculations based upon an initial cohort of 10,000 patients. In primary care, it is estimated that the volume of tests would approach 75,000 per annum. The volume of tests in secondary care is estimated at 53,000 per annum.

Main Outcome Measures

The primary outcome measure was quality adjusted life years (QALYs), which were employed to derive incremental cost-effectiveness ratios (ICERs) in a cost-effectiveness analysis.

Results

Results indicate that using a blood-based spectroscopic test in both scenarios has the potential to be highly cost-effective in a health technology assessment (HTA) agency decision making process, as ICERs were well below standard threshold values of £20,000 to £30,000 per QALY. This test may be cost-effective in both scenarios with test sensitivities and specificities as low as 80%; however, the price of the test would need to be lower (less than approximately £40).

Conclusion

Use of this test as triage tool in primary care has the potential to be both more effective and cost saving for the health service. In secondary care, this test would also be deemed more effective than the current diagnostic pathway.

For peer review only

Strengths and Limitations of this Study

- Simplified models of clinical pathways were mapped with input from, and consensus among, a wide range of clinical experts including neurosurgeons, neurooncologists, neuropathologists, neuroradiologists, and primary care experts.
- The spectroscopic blood test was highly sensitive and specific in retrospective data, with performances of 91.5 and 83.0% respectively. There is potential for this to contribute towards improved prognosis for patients, as well as healthcare savings.
- This study is based upon proof-of-concept studies, in advance of a pending prospective clinical trial. As these samples are retrospective, there is the possibility the diagnostic performance will not be as high in prospective studies.
- A lack of clinical trial evidence necessitates the estimate of long-term benefits of improved diagnostic protocols based on disease natural history models. This creates additional uncertainties.
- The precise patient population for whom the test may be suitable in the primary care setting is difficult to establish at this stage in development. This study considers a limited definition of eligibility that may need revision in light of future evidence.

For peer review only

Introduction

At an average of 20 years, patients with malignant brain tumours have the highest number of years of life lost, compared to all other primary cancers.[1] This, at least in part, may relate to diagnostic delays, reflecting the non-specific early symptoms, such as headache and dizziness, from which general practitioners (GPs) must identify patients at risk for further investigation. The lack of a low-cost diagnostic and/or screening tools available within the health service contributes to this delay. We have recently demonstrated that a spectroscopic test using blood-serum is able to effectively identify brain tumours in patients with sensitivities and specificities as high as 91·5% and 83% respectively, in a tissue bank case-control series.[2,3] This approach is based upon Fourier-transform infrared (FTIR) spectroscopy and can detect disease-specific signatures, which are extracted mathematically using pattern recognition and machine learning algorithms.

Current Diagnostic Pathway

Currently, patients who are symptomatic with a brain tumour visit their GP on average five times before being referred to secondary care.[4] Partly as a result of this diagnostic delay up to 61% of brain tumour diagnoses occur in an emergency setting, often following a seizure.[5] Patients diagnosed by the emergency route have a poorer prognosis.[6,7] For some patients this may be because the disease is at a more advanced stage at diagnosis. The complications precipitating the emergency admission make an additional contribution to mortality.

Screening programmes for breast, prostate, cervical, and colorectal cancer have proved effective for diagnosing patients at an earlier stage, which can result in a better prognosis.[8-11] These screening programmes have had a significant impact in reducing the number of patients presenting as an emergency. To date there has been no accessible and economically viable diagnostic tool for early detection of asymptomatic and symptomatic brain tumours.[12] The addition of a rapid and accurate blood test for patients with suspected primary brain tumours (symptomatic patients) therefore has the potential to improve outcomes by allowing prioritisation of patients most at risk of a brain tumour for further investigation. Under the current patient pathway it is not feasible to provide fast-track diagnostic imaging because the number of patients with non-specific headache symptoms is very large and the positive predictive value (PPV) on the basis of symptoms alone is less than 3% for all symptoms other than a new-onset seizure.[13,14]

Magnetic resonance imaging (MRI) and computed tomography (CT) are the current gold standard for identifying structural brain lesions including tumours. Treatment decisions made at the neuro-oncology multidisciplinary team (MDT) meeting are often based on the imaging alone. Following surgical resection or biopsy with histopathology and molecular analysis, definitive treatment can be planned.[15] Surgery to secure the tissue diagnosis has a small risk to the patient of neurological deterioration and death.[16]

The diagnostic pathway also represents a significant cost burden to the health service, with a single MRI and CT scan in the UK costing around £164 and £85, respectively (National Schedule of Reference Costs [2014-15], Medicare Physician Fee Schedule 2016). A typical

For peer review only - http://bmjopen.bmj.com/site/about/guidelines.xhtml

timeframe of the diagnostic pathway for brain tumours, specifically primary gliomas, is illustrated in Figure 1, and effectively highlights the significant wait that a symptomatic patient may have before receiving brain imaging. Even from this stage, regardless of the time to GP referral or emergency presentation, full diagnosis may take a further 5 weeks.

Serum Spectroscopic Diagnostics

This novel blood test for early brain tumour detection is based upon the interaction of infrared (IR) light with biological components of blood serum (**Figure 2**). Specifically, using attenuated total reflectance (ATR)-FTIR spectroscopy, specific bond vibrations of given molecules can be elucidated from serum samples, thus providing a unique insight into the composition via an absorbance spectrum.[17] Benefits of an ATR-FTIR based approach include a robust, user-friendly methodology without extensive sample preparation, that would readily fit into a clinical setting.[18] In short, serum is obtained according to standard protocols and can be snap frozen and stored at -80° until the point of analysis. A small volume of serum is required for analysis (1-5 μL), which is pipetted onto a crystal, known as an internal reflection element (IRE), where IR non-destructively interacts with the sample and produces an IR spectrum, with peaks representative of known bond vibrations and hence biomolecular constituents.

Blood serum is a complex medium that contains a variety of biomolecules, including around 20,000 proteins, that may be employed as diagnostic biomarkers.[19] In the case of brain tumours, such blood based technologies are limited, due to a lack of an established brain tumour specific diagnostic biomarker.[20] With our spectroscopic approach, rather than derive single biomarker specific information, a global signature is obtained, which encompasses the entire biomolecular makeup. This is epitomised as an equally complex biological absorbance spectrum, which contains a wealth of diagnostic information (an example may be seen in **Figure 3**).

Pattern recognition and machine learning algorithms using spectral features from FTIR data have been demonstrated as rapid and accurate for separating primary brain cancer and non-cancer cases.[2,21] When these algorithms are applied to this information rich dataset, the relationship between all biological components of the sample is ascertained, providing a multi-dimensional analysis of the sample.

In the case-control setting, this approach has been able to not only detect between cancer and non-cancer, but also stratify based upon cancer pathology.[2] For further information and in-depth description of the methodology, we direct the readers to the following fundamental review and recent research papers.[2,3,17,21] The ability to triage patients likely to have a brain tumour based on serum sample alone raises the possibility of systematic triaging prior to investigation with more expensive (MRI/CT imaging) and invasive (biopsy) tests. One major impact of having a serum test available would be a possible reduction in the number of unnecessary brain scans; however, as this test is also able to differentiate between primary and secondary tumours, there could also be a knock-on reduction of chest and abdomen scans which are conducted to rule out primary disease elsewhere. There is also the possibility that

For peer review only

this approach will reduce the incidence of incidental abnormalities which in themselves can cause considerable distress. Ultimately, it is expected that this could allow earlier and potentially more effective treatment of brain tumours.

It is important to note that this study is based upon proof-of-concept studies, in advance of a pending clinical trial. As these samples are retrospective, there is the possibility the diagnostic performance will not be as high in prospective studies. Additional to determining the true diagnostic accuracy of the technique, the planned clinical trial held in primary care will also reveal the suitable patient population for the test, as well as the long-term benefits of an improved diagnostic pathway.

Aims & Objectives

The aim of the economic evaluation is to assess the potential cost-effectiveness of this spectroscopic technology, in advance of any prospective study results being available. There are three main objectives for the evaluation. The first objective is to create a map of where the test could be used in the clinical pathway. The second is to assess the potential cost-effectiveness of the technology, if the performance shown in the case-control study is replicated prospectively. This will give an indication of whether the technology would meet the criteria for acceptance for use in the National Health Service that are applied by Health Technology Assessment agencies (HTA). Related to this, the third objective is to define the level of performance in prospective trials, and any additional evidence that would be needed to meet the cost-effectiveness criteria of an HTA decision-making process. This can include diagnostic performance and also effects on long-term outcomes such as survival and resource use. To achieve these objectives a simplified economic model of two important clinical scenarios is used to explore cost effectiveness.

For peer review only

Methodology

Mapping the Clinical Pathway

In order to appreciate the current clinical pathway and determine an appropriate entry point for a serum spectroscopic test, a pan-UK clinical focus group was established. This cohort included neurosurgeons, clinical and medical oncologists, neuropathologists, neuroradiologists, academic GPs with special interests, and experts in primary care diagnostics (see Appendix 1).

Cost Effectiveness Analysis

A cost-effectiveness analysis (CEA) was conducted to calculate the effects on health outcomes and health service costs of introducing spectroscopic testing in each of two scenarios. The health outcomes considered were life-years and quality-adjusted life-years (QALYs).

A decision tree was used to model the pathway for patients presenting with symptoms warranting a referral for MRI/CT imaging for suspected brain tumour (**Figure 4**). Separate models were considered for primary and secondary care. The time horizon of the model is 2 years and the perspective is that of the health care service. A two-year horizon was selected because of the short duration of survival in this patient group: median survival is approximately 1 year for high grade gliomas, which are the commonest malignant primary brain tumour. In all scenarios the comparator is the current diagnostic pathway (i.e. imaging alone). Further details regarding the node probabilities, simplifications and assumptions of the model can be found in **Appendix 2**.

Diagnostic Performance

The sensitivity and specificity of the test for detecting brain tumours has been demonstrated in a series of case-control studies using historical samples from the Brain Tumour North West and Walton Centre NHS bio-banks.[2,3] In the key case-control study nine FTIR spectra were collected from each of 433 patients.[3] Of these 134 were from patients with primary brain tumours (64 high grade glioma), 177 were from patients with cerebral metastases and 122 were from non-cancer controls. FTIR spectra were analysed using the random forest method to fit a classification model. Classification performance was estimated by applying the fitted model to a test set containing 20% of the patients from the original data set that were not used in the model fitting step (hold-out test set). Classification statistics are computed as averages of this process, iterated 96 times using random training and test sets. Under the best available model, sensitivity estimated by this method was 92·8% and specificity was 91·5% for the analysis of cancerous vs. non-cancerous serum.[3] These classification statistics were established using 96 independent iterations of a Random Forest model, and resulted in standard deviations of 1.1% and 1.9% respectively.

Establishing whether the performance demonstrated case-control data from historical samples translates to equivalent performance when applied prospectively in clinical practice is the subject of a planned clinical trial. This is critical to demonstrating the clinical and cost-

For peer review only

effectiveness of a serum spectroscopy test as part of the diagnostic pathway for brain tumours.

Prevalence of disease

Prevalence data were sourced from the literature based on clinical expert guidance. Brain tumour prevalence in scenario 2 (secondary care) was assumed to be 3% based on observed rates of primary brain tumour diagnosis among patients referred for brain imaging for suspected cancer in secondary care.[22-24] In scenario 1 (primary care) an estimated prevalence of 0·5% is used based on case-control evidence and expert opinion of the prevalence among patients who would be considered for direct access imaging, using MRI or CT, where this is available and referral to neurology where it is not.[25,26] An alternative prevalence of 1% was explored for scenario 1 based on unpublished data from a direct access imaging service in the UK (P. Brennan, personal communication).

The effect of serum spectroscopy testing on the time-to-diagnosis and time-to-treatment is discussed in Appendix 3, additional to the effect of testing on the use of imaging studies, and also on the patient outcomes. The primary assumptions in this model are that first, the expected time-to-diagnosis would match the current median time-to-diagnosis for patients presenting with brain tumour in emergency care as observed in Aggarwal et al.[5] Furthermore, based upon expert opinion, it is assumed that in secondary care all patients would continue to imaging, while in primary care 50% would continue to imaging following a negative spectroscopy result. This is a conservative estimate associated with the possibility that an imaging test will still be required in some cases based on interpretation of a patient's symptoms and the other non-tumour diagnoses being considered by the clinician. Finally, the effects of early diagnosis on the outcome of brain tumours is estimated using literature describing fitting natural history models to observational datasets of high grade glioma patients.[27,28]

Utility Weights

Health state utility weights are applied to life-years to generate quality adjusted life years (QALYs). A systematic review of health state utility weights for high grade glioma, the most common and aggressive primary brain tumour, was conducted to identify suitable utility weights. Due to heterogeneity it was not considered suitable to pool the estimates. The most appropriate health state utility weight was taken from a previous UK economic evaluation of glioblastoma treatment.[29] A value of 0·89 was used in the base case.

Resource Use and Costs

Resource use includes the application of a spectroscopic serum test to all patients prior to imaging, the imaging studies used in the diagnostic process, outpatient neurology clinic visits and general practitioner visits. In the UK analysis, unit costs for imaging studies are taken from UK NHS reference costs (2014/15), clinic and GP visits from the PSSRU costs schedule (Table 1). In the USA analysis, unit costs are taken from Medicare reimbursement schedules. Unit costs of the test were applied at an upper bound and lower bound rather than a single value as these products have not yet been commercialized. Bounds were set by consultation

For peer review only

with scientists developing the tests. Additional resource use and cost assumptions are described in Appendix 3.

Table 1. Unit costs and comparison for the brain tumour diagnostic pathway

Item	UK Cost per unit (£) (2015 prices)	USA cost per unit ($) (2016 prices)	Source(s)
CT imaging study [CT head]	85	163	National Schedule of Reference Costs (2014 – 15), Medicare Physician Fee Schedule 2016
MRI imaging study [MRI brain with contrast]	164	380	National Schedule of Reference Costs (2014 – 15), Medicare Physician Fee Schedule 2016
Neurology outpatient appointment	35	76	PSSRU 2016, Medicare Physician Fee Schedule 2016
GP visit	47·25	76	PSSRU 2016, Medicare Physician Fee Schedule 2016
Stable disease monitoring costs	116 per 3 months	154	[29], exchange rate adjusted
Serum spectroscopy test	Lower limit: 50<br>Upper limit: 100	Lower limit: 100<br>Upper limit: 200	Assumed prices

Incremental cost effectiveness ratios (ICER)

The comparative cost-effectiveness of spectroscopic testing compared to no testing is summarized by the ICER defined as:

$$ICER = \frac{C_s - C_n}{H_s - H_n}$$

C_s and C_n are the total costs with spectroscopic testing and no testing respectively. Equivalently H_s and H_n are the total QALYs with and without spectroscopic testing. The ICER can be interpreted as the additional cost per QALY gained.

For peer review only

Base Case and Sensitivity Analysis

ICERs were calculated for scenarios 1 and 2 using the base case parameter estimates. Base case analysis was repeated for UK and USA settings. Additional sensitivity analyses are reported for the UK setting only.

Sensitivity analyses included one-way sensitivity analysis (OWSA), systematically varying a single parameter in the model, and scenario analysis in which specific model assumptions were altered. OWSA were conducted for test sensitivity, specificity and test cost. Scenario analyses included assuming an additional consultation cost for discussion of test results, assuming a higher proportion of patients continue to imaging following a negative spectroscopy result in primary care, and using mean survival rather than median survival.

A probabilistic sensitivity analysis (PSA) was conducted to explore the effects of joint uncertainty in the parameter estimates on the model results.[30]

Patient and Public Involvement

Patients and the public were not actively involved in the formation of this study. The impact of the test on clinical decision making was the priority in this instance; however, the involvement of patients going forward will be fundamental to understanding the tests uptake into the health services.

For peer review only

Results

Mapping the clinical pathway

For the first study objective, initial discussion with clinical experts indicated that there are potential uses for the test in both primary and secondary care. The main advantage of employing a cost-effective spectroscopic blood test in the diagnostic pathway is to use it as a triage test. This prioritises more urgent cases for access to services, and acts as a gate-keeper (requiring a positive result in some conditions to give access to services) for imaging studies.

Two clinical scenarios are mapped out below and subsequently explored in this early economic evaluation of the serum spectroscopy test for aiding diagnosis of brain tumours:

1. Triage tool in primary care

The primary care scenario explores a population of patients with a clinical presentation that warrants further investigation of possible brain tumour. This would include some patients with headaches and some with focal neurological deficits. This is the group of patients who would be considered for direct access imaging, using MRI or CT, where this is available and referral to neurology where it is not.[31] The blood test is used to provide rapid information, within 24 hours, where a positive result would lead to patients receiving more timely access to imaging. It may also be the case that negative test results, in addition to establishing the low probability of a brain tumour, could also provide some reassurance for those patients that must wait for imaging. The total volume of tests would be approximately 75,000 per year in the UK (see Appendix 4 for further details).

2. Triage tool in secondary care

In this scenario the population is the group of patients that are currently referred for imaging studies from secondary care for suspected brain tumour, typically via neurology clinics. This is the patient group for whom the clinical presentation has the highest positive predictive value (PPV). However, even in this high risk group, the odds of a brain tumour being present are approximately 1:33.[22-24] Again, the spectroscopy test is used to provide rapid information to allow a subset of these patients to access immediate imaging and provide reassurance to other patients who may have to wait longer for definitive imaging studies and diagnosis. The extent of the benefits of triage in this scenario is likely to vary by locality depending on the capacity constraints on imaging and pathology services. This evaluation uses estimates of the delays in diagnosis, and potential improvements in the speed of diagnosis, from a consecutive patient case series in London, UK.[5] The total volume of tests if this scenario occurred would be approximately 53,000 per year in the UK (see Appendix 4 for further details).

For peer review only

Cost Effectiveness Assessment

The standard threshold value per QALY gained in the UK, is considered to be between £20,000 to £30,000. Below this value, a healthcare intervention may be considered cost effective, whereas a negative ICER value would be deemed cost saving. Base case results for primary care (scenario 1) and secondary care (scenario 2) are presented in Table 2. Note results are reported for cohorts of 10,000 patients.

Table 2. Incremental QALYs, costs and ICERs for Scenarios 1 and 2, UK and USA

	**Scenario 1 – Primary Care**			**Scenario 2 – Secondary Care**		
**Serum spectroscopy test cost (£)**	**ΔQALY**	**ΔCost**	**ICER**	**ΔQALY**	**ΔCost**	**ICER**
**UK**						
**50**	8·81	-422,116	-47,913 (dominates)	52·86	527,646	9,982
**100**	8·81	77,884	8,840	52·86	1,027,646	19,441
**USA**						
**100**	8·81	-1,718,475	-195,058 (dominates)	52·86	536,702	10,153
**200**	8·81	-218,475	-24,798 (dominates)	52·86	2,036,702	38,530

ΔQALY, ΔCost: difference is QALYs/Costs (with serum spectroscopy test – without test), 10K patients
ICER: Incremental cost-effectiveness ratio

Incremental cost-effectiveness ratios were well below standard threshold values of £20,000 to £30,000 per quality adjusted life year (QALY) gained used in the UK, and similar thresholds used internationally, provided the test cost did not exceed £100. The base case results demonstrate that the serum spectroscopy test dominates (more effective and less costly) standard care at the lower bound of test cost in the primary care setting in both the UK and USA. At the upper bound of test cost the ICERs may be within commonly used thresholds or, in the case of the USA, remain dominant to standard care. In the secondary care setting ICERs of £9,982 and $10,153 at the lower bounds of test cost indicate that this test is potentially cost-effective in this setting. At the upper bounds ICERs may still be within commonly used thresholds for cost-effectiveness.

Sensitivity Analysis Results

The performance of the test with regards to levels of sensitivity and specificity are addressed using sensitivity analysis. OWSA results for a range of test specificities are displayed in Figure 5 and 6, for primary and secondary care respectively, displaying the ICER with

For peer review only

varying test specificity. Note that the estimated QALYs do not change with specificity in the model therefore changes in the ICER are due solely to changes in incremental costs. Varying sensitivity changes both estimated QALYs and estimated costs therefore results of the OWSA for test sensitivity are presented on the cost-effectiveness plane (Appendix 5). In primary care, using the upper cost limit of £100 it is evident that the test is deemed cost effective at specificities of around 0·9 and above, where the ICER is below standard thresholds. In contrast, at the lower cost limit, the test is cost effective at specificities above 0·8. Although the serum spectroscopy test is not cost saving at low cost (or near perfect specificities), the test is still considered cost effective at specificity levels around 0·7 and 0·8 for £50 and £100 pricing respectively.

The OWSA results highlight how ICERs in scenario 2 (secondary care) are strongly influenced by test sensitivity while ICERs in scenario 1 (primary care) are more strongly influenced by test specificity. These features are a result of the varying prevalence of disease and the assumption in scenario 2 that 100% of patients are referred for imaging following negative result. It should be noted that test specificity is important in both scenarios. Relatively small improvements in test specificity can substantially change the ICER, while larger improvements in test sensitivity are required to substantially alter the ICERs.

Additional scenario analyses are also reported in Appendix 5. These demonstrate that results are robust to using mean survival estimates rather than median survival estimates and including additional consultation costs for positive test results. If the prevalence of brain tumours in Scenario 1 is 1% rather than 0·5% the incremental QALYs increase substantially and the ICERs are reduced.

The PSA results reported in Figure 7 and 8 indicate that at a test cost of £50 and an ICER threshold of £30,000 per QALY there is a near 100% probability the serum spectroscopy test is cost-effective in scenario 1 and approximately 90% probability it is cost-effective in scenario 2. The corresponding probabilities at the upper bound cost of £100 are approximately 85% and 75%.

For peer review only

Discussion

This economic evaluation establishes the potential for serum spectroscopy to have a role in the diagnosis of both benign and malignant brain tumours in both primary and secondary care. The potential costs and health benefits of testing using a spectroscopic method prior to CT/MRI tests (or in some scenarios to avoid imaging) have been estimated based on a mathematical model with parameter values taken from published studies and expert opinion. This diagnostic tool is sensitive to all brain tumours (benign or malignant), however, this assessment is closely aligned with the diagnosis of primary gliomas, where there is a maximum potential benefit to the health service.

The major limitations of this analysis relate to the use of proof-of-concept studies and a disease natural history model rather than direct clinical trial evidence. This creates additional uncertainties. Results should be interpreted as indicative and used primarily to guide future evidence generation. Furthermore, the scenarios explored were limited in scope; future studies should continue to refine understanding of the role of the test in real-world clinical decision making.

When used as a triage tool in primary care, this novel test has the potential to deliver improvements in health outcomes and also to reduce costs. At the lower end of test costs, the technology would be cost-saving for the health service. At higher test costs the technology is still likely to be considered cost-effective in HTA agency decision processes.

In Scenario 2, in which serum spectroscopy is used as a triage tool in secondary care, the technology will create additional costs but also produce sizable health benefits. At test costs of under £100 ($200) the technology would be likely to be considered as a cost-effective use of resources in HTA agency decision processes in the UK (and USA). It is assumed that in both scenarios, the uptake of the test in the USA would mirror that of the UK; however, this would need to be explored further, alongside clinical experts of the USA care pathways.

Sensitivity analyses have demonstrated the importance of diagnostic performance on the cost-effectiveness of the test. In particular, test specificity is important in the primary care setting. If test specificity is 87·5% or worse the technology may not be considered cost-effective at higher values of assumed test cost. This is due to the increased number of false positive results in this low prevalence population, generating a greatly increased proportion of fast-track imaging studies which increases costs.

To strengthen the case that this approach represents a cost-effective use of healthcare resources it is necessary to establish the diagnostic performance of the test prospectively. This can be accomplished by a suitably large cohort study in which serum spectroscopy is used alongside current clinical practice in one of the patient groups included in this evaluation. It would be appropriate to initially target the secondary care patient population, because the higher prevalence of disease in this group will reduce the sample size needed to accurately estimate diagnostic performance.

For peer review only

Decision makers are often most interested in patient outcomes, such as survival, rather than intermediate outcomes, such as accuracy or speed of diagnosis (although this latter point is vital for treatment of high grade gliomas). From this perspective, a randomized trial, or a prospective cohort study with extended follow-up, may be required to fully establish the size of survival and quality-of-life benefits of including a serum spectroscopy test in the diagnostic pathway. A trial with primary outcomes relating to survival and quality-of-life would be specific to either the primary or secondary care setting (rather than generalisable to both), would need a large sample size, and would also require a follow-up period to capture survival benefits. In the case of malignant glioma this would require a period of at least 24 months. Such a trial would clearly be expensive, time consuming and may be unfeasible. Decision makers may be willing to make a decision on implementation of the blood test based on the modeled effects of improvements in intermediate outcomes on later patient outcomes. In this situation, the model proposed in this evaluation, populated with diagnostic performance and other data from a prospective trial, could be used to inform decisions about the wider adoption of the technology.

Future developments beyond trials such as emerging epidemiological evidence and new technologies should also be included in any future evaluations. It was not possible to foresee and include all such possible scenarios in this early evaluation but that should not preclude assessment in the light of new evidence. Updated analysis should inform any decisions about system wide implementation.

Several results in this analysis suggest cost savings through reduced use of imaging for patients with a negative test result. To make the case that a serum spectroscopic test can improve the efficiency of the diagnostic pathway prospective studies will also need to explore the impact of these test results on clinician and patient imaging study decisions. The possibility remains that the test may triage patients, but may not reduce the number of scans being conducted, and could potentially increase the demand on imaging. For example, if the test is applied to a wider population than intended in primary care due to the availability of such a non-invasive test effectively lowering the threshold for investigation. This highlights the need to stud decision making in this area prior to any implementation in primary care. Nevertheless, this triaging of patients would still benefit each patient that is provided with an early diagnosis.

This evaluation has explored the potential for serum spectroscopy to be a cost-effective addition to the diagnostic pathway for brain tumours. It has demonstrated that in specific scenarios this novel test may be an effective and cost-effective technology in reducing the delay to diagnosis for patients with brain tumours. Prospective trials are required to provide definitive evidence.

For peer review only

References

1 Burnet, N., Jefferies, S., Benson, R., Hunt, D. & Treasure, F. Years of life lost (YLL) from cancer is an important measure of population burden—and should be considered when allocating research funds. *Brit J Cancer* 2005; **92**: 241–5.

2 Hands, J. R., Clemens, G., Stables, R. *et al.* Brain tumour differentiation: rapid stratified serum diagnostics via attenuated total reflection Fourier-transform infrared spectroscopy. *J Neurooncol* 2016; **127**: 463–72.

3 Smith, B. R., Ashton, K. M., Brodbelt, A. *et al.* Combining random forest and 2D correlation analysis to identify serum spectral signatures for neuro-oncology. *Analyst* 2016; **141**: 3668–78.

4 Finding a better way? Improving the quality of life for people affected by brain tumours, Report Published by The Brain Tumour Charity, 2014.

5 Aggarwal, A., Herz, N., Campbell, P. *et al.* Diagnostic delay and survival in high-grade gliomas - evidence of the 'waiting time paradox'? *Br J Neurosurg* 2015; **29**: 520–3.

6 Zhou, Y., Abel, G. A., Hamilton, W. *et al.* Diagnosis of cancer as an emergency: a critical review of current evidence. *Nat Rev Clin Oncol* 2017; **14**: 45–56.

7 McPhail, S., Elliss-Brookes, L., Shelton, J. *et al.* Emergency presentation of cancer and short-term mortality. *Brit J Cancer* 2013; **109**: 2027–34.

8 Blanks, R., Moss, S., McGahan, C., Quinn, M. & Babb, P. Effect of NHS breast screening programme on mortality from breast cancer in England and Wales, 1990-8: comparison of observed with predicted mortality. *BMJ* 2000; **321**: 665–9.

9 UK Colorectal Cancer Screening Pilot Group. Results of the first round of a demonstration pilot of screening for colorectal cancer in the United Kingdom. *BMJ* 2004; **329**: 133.

10 Quinn, M., Babb, P., Jones, J. & Allen, E. Papers Effect of screening on incidence of and mortality from cancer of cervix in England: evaluation based on routinely collected statistics. *BMJ* 1999; **318**: 904.

11 Frankel, S., Smith, G. D., Donovan, J. & Neal, D. Screening for prostate cancer. *The Lancet* 2003; **361**: 1122–8.

12 Gadalla, S., Pfeiffer, R., Kristinsson, S. *et al.* Brain tumors in patients with myotonic dystrophy: a population□based study. *Eur J Neurol* 2016; **23**: 542–47.

13 Callaghan, B. C., Kerber, K. A., Pace, R. J., Skolarus, L. E. & Burke, J. F. Headaches and neuroimaging: high utilization and costs despite guidelines. *JAMA Intern Med* 2014; **174**: 819–21.

14 National Institute for Health and Care Excellence, 2015. Suspected cancer: recognition and referral. NICE Guideline (MG12).

15 Livermore, L. J., Ma, R., Bojanic, S. & Pereira, E. A. Yield and complications of frame-based and frameless stereotactic brain biopsy–the value of intra-operative histological analysis. *Brit J Neurosurg* 2014; **28**: 637–44.

16 Weber, M. A., Zoubaa, S., Schlieter, M. *et al.* Diagnostic performance of spectroscopic and perfusion MRI for distinction of brain tumors. *Cancer Imaging* 2006; **6**: S32–S41.

17 Baker, M. J., Trevisan, J., Bassan, P. *et al.* Using Fourier transform IR spectroscopy to analyze biological materials. *Nature Protoc* 2014; **9**: 1771–91.

18 Lovergne, L., Bouzy, P., Untereiner, V. *et al.* Biofluid infrared spectro-diagnostics: pre-analytical considerations for clinical applications. *Faraday Discuss* 2016; **187**: 521–37.

19 Baker, M. J., Hussain, S. R., Lovergne, L. *et al.* Developing and understanding biofluid vibrational spectroscopy: a critical review. *Chem Soc Rev* 2016; **45**: 1803–18.
20 Hochberg, F. H., Atai, N. A., Gonda, D. *et al.* Glioma diagnostics and biomarkers: an ongoing challenge in the field of medicine and science. *Expert Rev Mol Diagn* 2014; **14**: 439–52.
21 Hands, J. R., Abel, P., Ashton, K. *et al.* Investigating the rapid diagnosis of gliomas from serum samples using infrared spectroscopy and cytokine and angiogenesis factors. *Anal Bioanal Chem* 2013; **405**: 7347–55.
22 Wang, H. Z., Simonson, T. M., Greco, W. R. & Yuh, W. T. C. Brain MR Imaging in the Evaluation of Chronic Headache in Patients without Other Neurologic Symptoms. *Acad Radiol* 2001; **8**: 405–8.
23 Sempere, A. P., Porta-Etessam, J., Medrano, V. *et al.* Neuroimaging in the evaluation of patients with non-acute headache. *Cephalalgia* 2005; **25**: 30–5.
24 Clarke, C. E., Edwards, J., Nicholl, D. J. & Sivaguru, A. Imaging results in a consecutive series of 530 new patients in the Birmingham Headache Service. *J Neurol* 2010; **257**: 1274–9.
25 Kernick, D., Stapley, S., Goadsby, P. J. & Hamilton, W. What happens to new-onset headache presented to primary care? A case-cohort study using electronic primary care records. *Cephalalgia* 2008; **28**: 1188–95.
26 Hamilton, W. & Kernick, D. Clinical features of primary brain tumours: a case–control study using electronic primary care records. *Brit J Gen Pract* 2007; **57**: 695–9.
27 Do, V., Gebski, V. & Barton, M. The effect of wating for radiotherapy for grade II/IV gliomas. *Radiother Oncol* 2000; **57**: 131–6.
28 Burnet, N., Jefferies, S., Benson, R., Hunt, D. & Treasure, F. Years of life lost (YLL) from cancer is an important measure of population burden—and should be considered when allocating research funds. *Brit J Cancer* 2005; **92**: 241–45.
29 Garside, R., Pitt, M., Anderson, R. *et al.* The effectiveness and cost-effectiveness of carmustine implants and temozolomide for the treatment of newly diagnosed high-grade glioma: a systematic review and economic evaluation. *Health Technology Assessment* 2007; **11**.
30 National Institute for Health and Care Excellence, 2013. Guide to the methods of technology appraisal. NICE Guideline (PMG9).
31 Simpson, G. C., Forbes, K., Teasdale, E., Tyagi, A. & Santosh, C. Impact of GP direct-access computerised tomography for the investigation of chronic daily headache. *Br J Gen Pract* 2010; **60**: 897–901.

For peer review only

FIGURE LEGENDS

Figure 1. The diagnostic pathway of brain tumours. Timings relate to the diagnosis of high grade gliomas and are based upon discussion with the Clinical Focus Team and Aggarwal et al. (2015)[5]. (*OPD – Outpatient department; MDT – Multidisciplinary team*)

Figure 2. Attenuated total reflectance (ATR)-Fourier transform Infrared (FTIR) spectroscopic test of blood serum for the diagnosis and stratification of brain tumours using machine learning algorithms.

Figure 3. An unprocessed spectrum derived from human blood serum using ATR-FTIR spectroscopy. Spectral regions correspond to known bond vibrations and can therefore be associated with groups of biomolecules such as protein, lipid, phosphate and carbohydrates. Broad examples of blood serum constituents are listed.

Figure 4. A decision tree model describing the integration of a serum spectroscopy test in the current diagnostic pathway, and the effect on MRI/CT imaging for suspected brain tumour.

Figure 5 – Incremental cost effectiveness ratios (ICERs) at various specificities in primary care ('Scenario 1'). The £30,000 ICER threshold is displayed as a dashed horizontal line.

Figure 6 - Incremental cost effectiveness ratios (ICERs) various specificities in Secondary Care ('Scenario 2'). The £30,000 ICER threshold is displayed as a dashed horizontal line.

Figure 7 – Cost effectiveness acceptability curve (CEAC) at £50 and £100 per test – Primary care ('Scenario 1')

Figure 8 – Cost effectiveness acceptability curve (CEAC) at £50 and £100 per test – Secondary care ("Scenario 2").

For peer review only

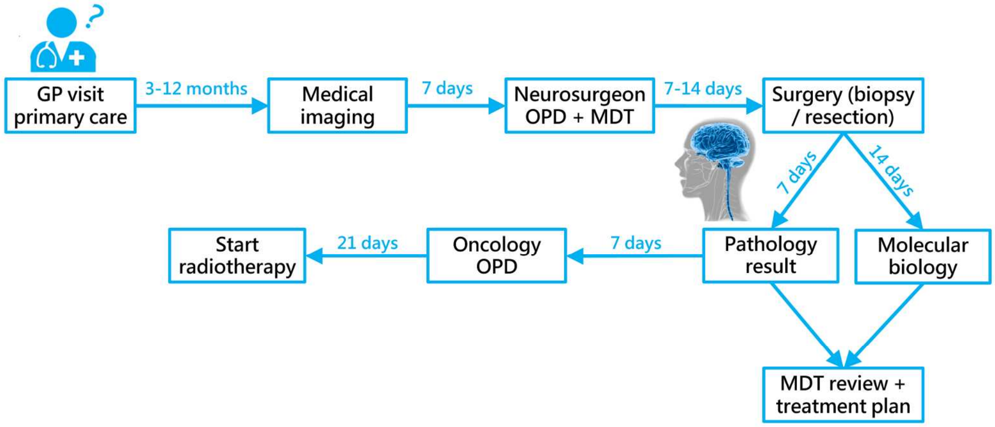

Figure 1. The diagnostic pathway of brain tumours. Timings relate to the diagnosis of high grade gliomas and are based upon discussion with the Clinical Focus Team and Aggarwal et al. (2015)5. (OPD – Outpatient department; MDT – Multidisciplinary team)

169x77mm (300 x 300 DPI)

For peer review only

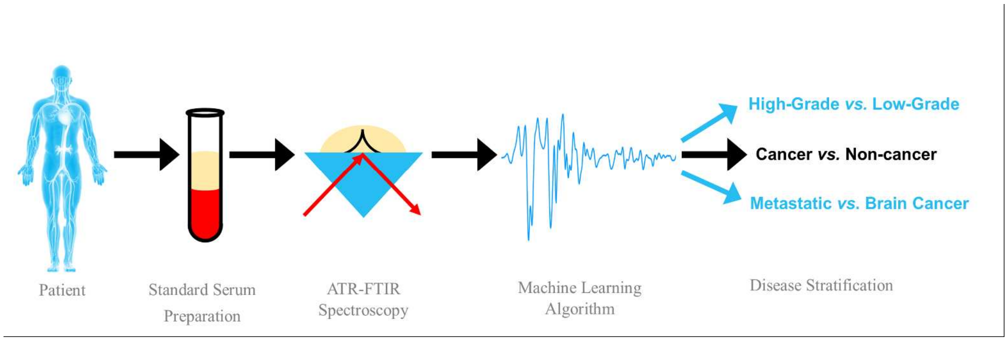

Figure 2. Attenuated total reflectance (ATR)-Fourier transform Infrared (FTIR) spectroscopic test of blood serum for the diagnosis and stratification of brain tumours using machine learning algorithms.

210x70mm (300 x 300 DPI)

For peer review only

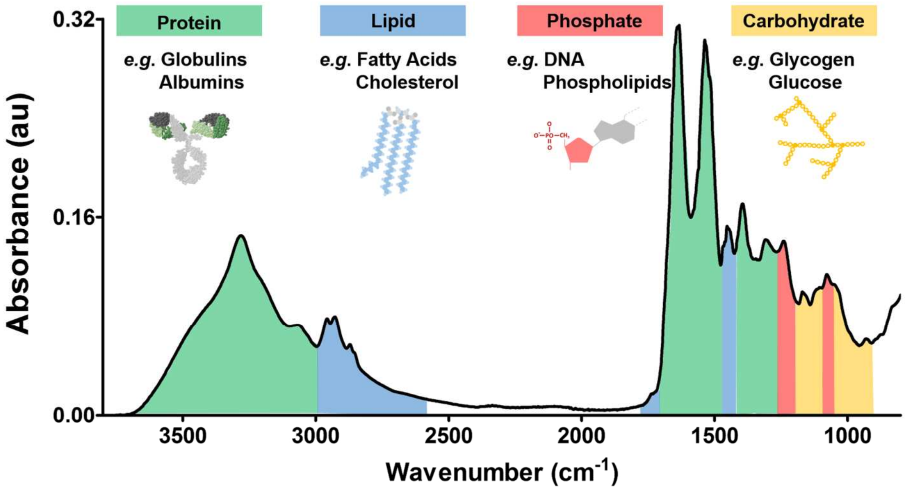

Figure 3. An unprocessed spectrum derived from human blood serum using ATR-FTIR spectroscopy. Spectral regions correspond to known bond vibrations and can therefore be associated with groups of biomolecules such as protein, lipid, phosphate and carbohydrates. Broad examples of blood serum constituents are listed.

169x95mm (300 x 300 DPI)

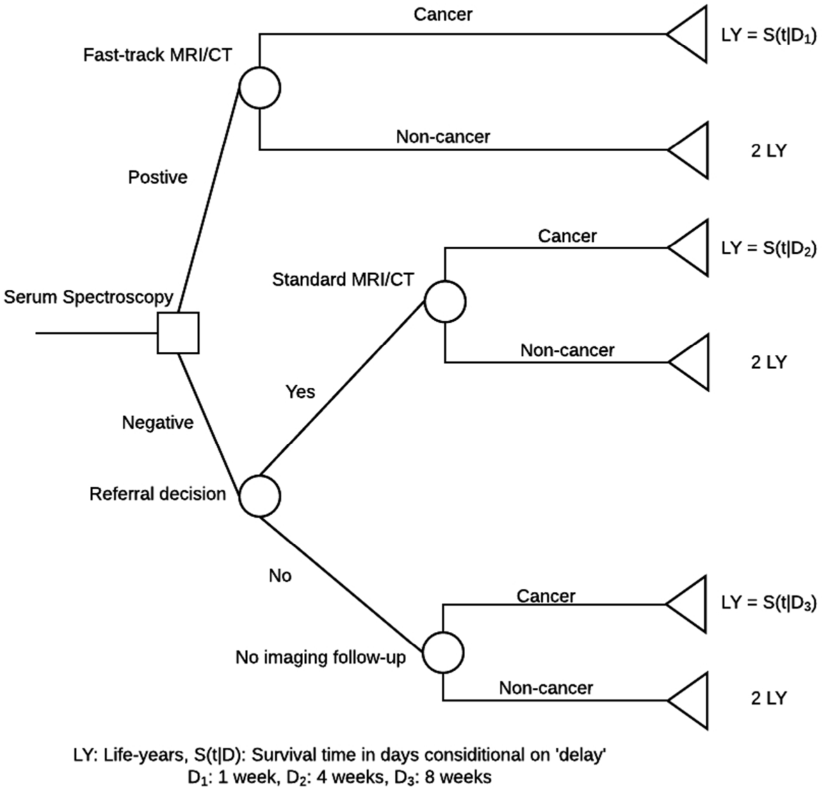

Figure 4. A decision tree model describing the integration of a serum spectroscopy test in the current diagnostic pathway, and the effect on MRI/CT imaging for suspected brain tumour.

82x74mm (300 x 300 DPI)

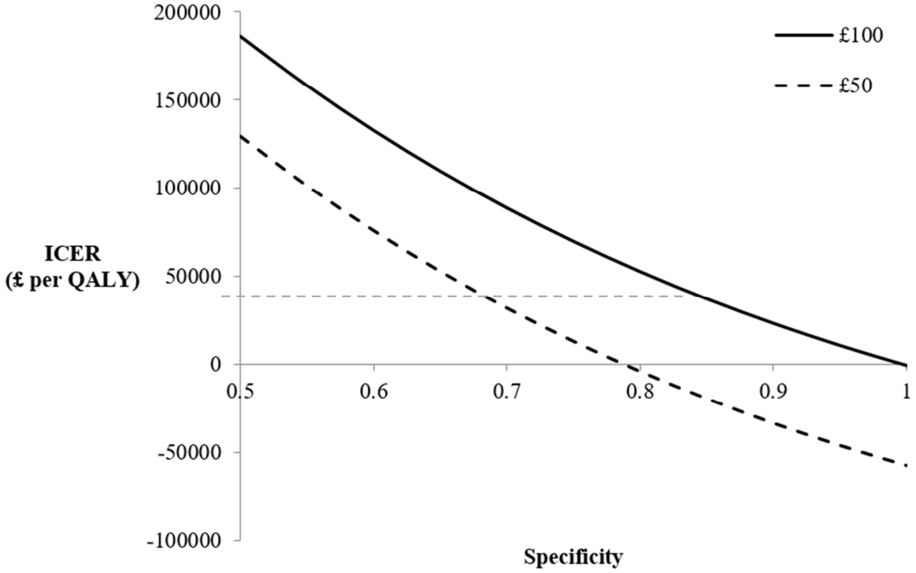

Figure 5 – Incremental cost effectiveness ratios (ICERs) at various specificities in primary care ('Scenario 1'). The £30,000 ICER threshold is displayed as a dashed horizontal line.

78x49mm (300 x 300 DPI)

view only

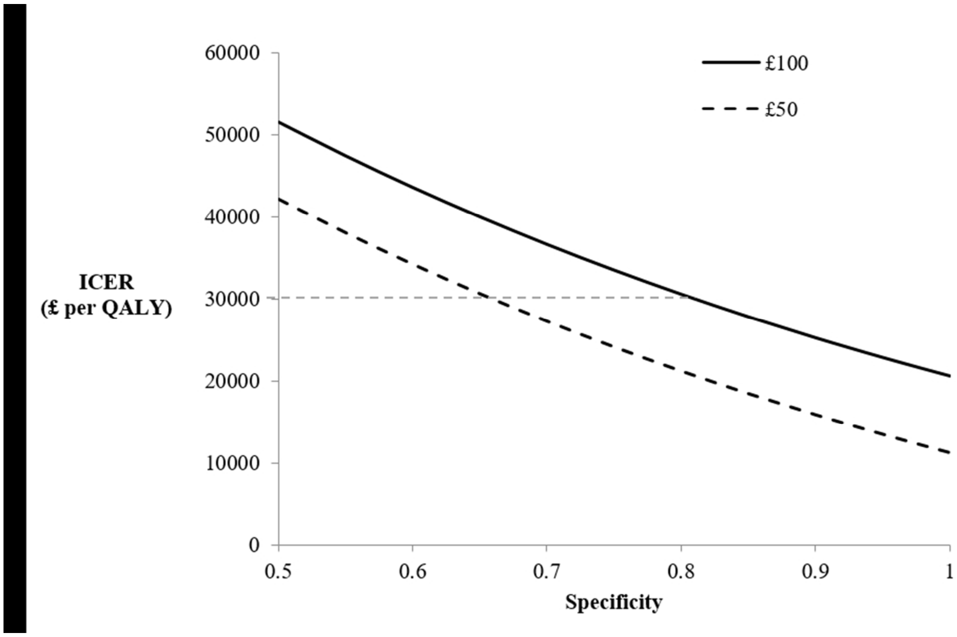

Figure 6 - Incremental cost effectiveness ratios (ICERs) various specificities in Secondary Care (‘Scenario 2’). The £30,000 ICER threshold is displayed as a dashed horizontal line.

80x50mm (300 x 300 DPI)

view only

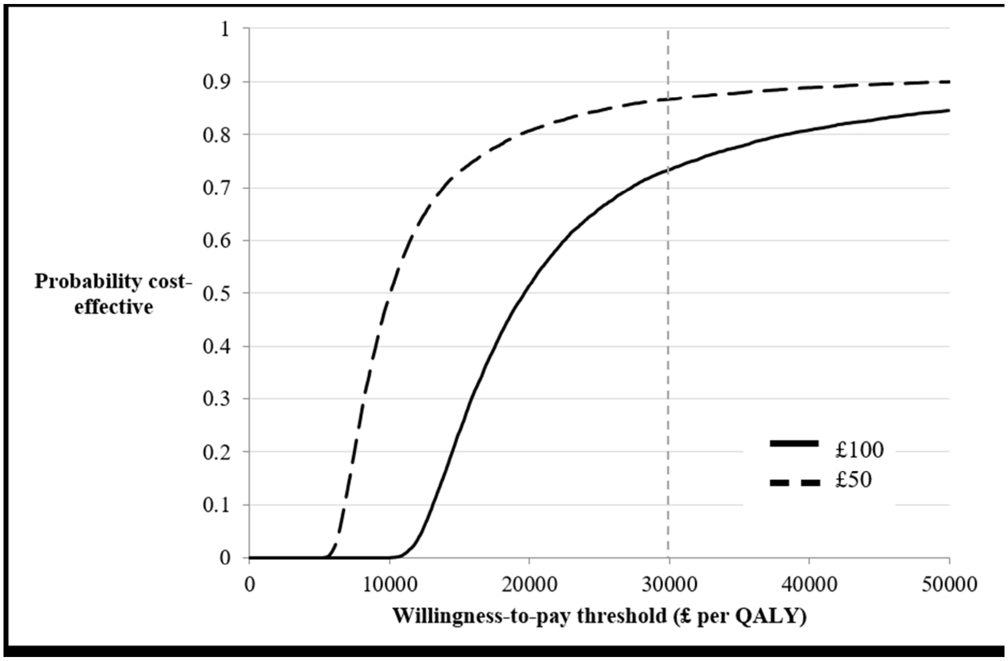

Figure 7 – Cost effectiveness acceptability curve (CEAC) at £50 and £100 per test – Primary care (‘Scenario 1’)

78x51mm (300 x 300 DPI)

For peer review only - http://bmjopen.bmj.com/site/about/guidelines.xhtml

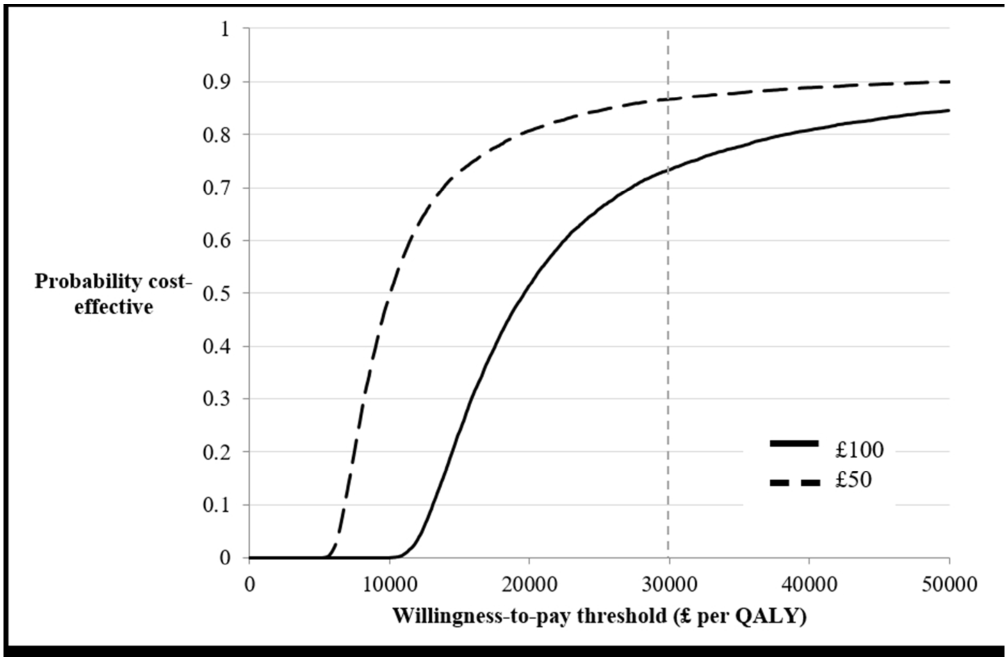

Figure 8 – Cost effectiveness acceptability curve (CEAC) at £50 and £100 per test – Secondary care ("Scenario 2").

78x51mm (300 x 300 DPI)

SUPPLEMENTARY MATERIAL

Appendix 1 – Clinical expert group

Clinical expert group participants: Mr Michael D Jenkinson (Walton Centre NHS Trust & University of Liverpool), Dr Paul Brennan (University of Edinburgh), Prof. Anthony Chalmers (University of Glasgow & Beatson Centre), Prof. Willie Hamilton (University of Exeter Medical School), Mr Kevin O'Neill (Imperial College Healthcare NHS Trust), Dr Allan Janes (Beatson West of Scotland Cancer Centre), Dr Babar Vaqas (Imperial College Healthcare NHS Trust), Dr David Kernick (St Thomas Health Centre, Exeter), Dr James Livermore (University of Oxford), Dr Ruth Board (Lancashire Teaching Hospitals NHS Trust), Prof. Timothy Dawson (Lancashire Teaching Hospitals NHS Trust), Miss Elvira Lekka (Lancashire Teaching Hospitals NHS Trust), Mr John Goodden (Leeds Teaching Hospital NHS Trust) and Dr. Samantha Mills (Walton Centre NHS Trust)

For peer review only

Appendix 2 – Decision tree model

The decision node probabilities that populate the decision tree model are reported in Table S1. The model is simplified by assuming that the reference test (MRI/CT) has perfect accuracy (100% sensitivity and 100% specificity). This is desirable if we wish to evaluate the test against an MRI/CT only diagnostic pathway in which these imaging studies are considered as the 'gold standard'. It is also difficult to obtain valid estimates of MRI/CT sensitivity and specificity for this indication as these are not reported in a comparable manner in the literature. This simplification will not substantially alter the economic results compared to using more realistic values for the scenarios under consideration. This is because it is assumed confirmation of the diagnosis always requires imaging and therefore outcomes for patients that would hypothetically be classified correctly using this test but incorrectly by MRI/CT will remain the same whether or not the test is used. The effect of less than perfect accuracy of the reference test is therefore the same as a (small) decrease in prevalence as a proportion of patients with the disease cannot benefit from the additional test due to incorrect subsequent testing. Outcomes are calculated by 'rolling-back' the tree for both cancer cases and non-cases and taking an average of the two groups weighted by the prevalence of brain tumours in the scenario population.

Table S1. Decision node probabilities for decision tree model describing the integration of a serum spectroscopy test in the current diagnostic pathway

**Cancer cases:**	**Probability to upper branch**	**Probability to lower branch**
Serum Spectroscopy Test (test sensitivity)	0·928	0·072
Standard MRI/CT (reference test sensitivity)	1	0
Fast-track MRI/CT (reference test sensitivity)	1	0
Referral decision	S1:0·5, S2: 1	S1:0·5, S2: 0
Non-cases:		
Serum Spectroscopy Test (test specificity)	0·085	0·915
Standard MRI/CT (reference test specificity)	0	1
Fast-track MRI/CT (reference test specificity)	0	1
Referral decision	S1:0·5, S2: 1	S1:0·5, S2: 0

S1 & S2: Scenarios 1 &2

For peer review only

Appendix 3 – Assumptions and details of cost effective analysis model

Effect of testing on time-to-diagnosis and time-to-treatment

To estimate the expected time-to-diagnosis under a fast-track pathway it is assumed that this would match the current median time-to-diagnosis for patients presenting with brain tumour in emergency care as observed in Aggarwal et al [5]. This study reported a cohort of high grade glioma patients from a single hospital in London, UK. No other suitable estimates of time-to-treatment or time-to-diagnosis were identified in the literature. This is the most common primary brain tumour in the indications considered in this evaluation. Median time-to-diagnosis for the standard pathway is also taken from the same source.

Effect of testing on use of imaging studies

The effect of spectroscopic testing on imaging study decisions of clinicians and patients is uncertain. Based on clinical expert input it was assumed, in the base case, that in secondary care all patients would continue to imaging, while in primary care 50% would continue to imaging following a negative spectroscopy result.

Effect of early diagnosis on patient outcomes

A systematic review of the literature failed to identify any studies that directly estimated the effect of earlier (or later) diagnosis on outcomes for primary brain tumours. Instead, based clinical expert guidance, estimates of the effects of time-to-treatment were sourced from the literature. The estimated effects are based on fitting natural history models to observational datasets of high grade glioma patients.[27,28] It is necessary to use a natural history model to produce survival estimates rather than using the survival data stratified by time-to-treatment directly, in order to adjust for potential confounding factors influencing both time-to-treatment and survival.

A one-to-one correspondence is assumed for the effects of an additional day added to the time-to-diagnosis and an additional day added to the time-to-treatment. Median survival by weeks between diagnosis and treatment are displayed in Table 2. The hazard ratio per additional day delay calculated in Do et al of 1·015 was applied to each day between diagnosis and treatment up to 28 days to calculate median survival time. Time between diagnosis and treatment beyond 28 days were fixed at the same median survival as 28 days.

Table S2. Relationship between time-to-diagnosis and survival

Time-to-diagnosis (weeks)	Median survival (weeks)
0	46
1	41·4
2	37·3
3	33·6
4	30·3

Source: Do et al 2000

Resource use and cost

It is assumed that there is no additional cost for converting a small proportion of standard referral to additional urgent referrals. This could be justified on the basis that patients receiving negative results allows more flexibility in the scheduling for these patients. This could create more opportunity to schedule patients with positive spectroscopy results sooner. As a larger proportion of patients are referred for immediate imaging it was assumed costs would increase because achieving this would require additional capacity. It was assumed that unit costs for MRI and CT grow exponentially with increasing proportion of immediate referrals such that costs double if 50% of patients are referred for immediate imaging.

Treatment costs are assumed to be the same for both fast-track and standard referrals that are diagnosed as cases. This may be a conservative assumption as treatment costs are likely to increase as disease progresses and therefore earlier treatment may, on average, be less costly than later treatment. However, it is also possible that

For peer review only

treatment costs could be increased if some cases are converted from palliative care to more aggressive treatment. The balance of these effects is unknown given the lack of appropriate data to estimate the effects. Secondary treatment and end-of-life costs are similarly assumed to be equal between fast track and standard referrals. This reflects the model assumption that all patients ultimately progress.

The time intervals considered (0-4 weeks) are too short for discounting to have an important impact on cost estimates therefore the effects of moving forward treatment on present value is not considered.

For peer review only

Appendix 4 - Total demand for serum spectroscopy tests in UK in two clinical scenarios

1. Primary care

An assumption that patients currently referred in direct-access imaging service would be selected for spectroscopic testing can provide a conservative estimate of the total number of patients likely to be tested. Three sources from the literature were considered:

1. A direct-access brain MRI service in Nottingham (UK) reported 130 patients per quarter (520 per year) among a population of 342,000.[32,33] The population of the UK is approximately 61 million therefore, assuming Nottingham is representative of the UK general population demand for brain MRI, there would be approximately 93,000 per year in the UK.
2. A direct access CT service for chronic headache in Glasgow (UK) reported 4404 patients referred over 8 years (551 per year) among a population of approximately 1 million.[31] Assuming this was representative of the UK generally then the demand for this type of service would be approximately 34,000 for the UK.
3. A direct access CT neuroimaging service in Lothian (UK) reported 389 exams per year for a population of approximately 442,000.[34] Assuming this was representative of the UK generally then the demand for this type of service would be approximately 54,000 for the UK.

The Glasgow estimate is particularly low and may result from limited uptake in the early part of the 8 year period for which data were reported. As it was not possible to determine if uptake increased over the period or was stable this estimate was not considered reliable. The Nottingham and Lothian estimates report potentially more stable and established services and are therefore more likely to reliably estimate demand. The likely demand for this novel test, if the indication is considered identical to neuroimaging, is therefore between 54,000 and 93,000. This is likely to be conservative as the test may well have a slightly broader indication than current neuroimaging. A reasonable point estimate may be approximately 75,000.

2. Secondary Care

An estimate of the total demand for serum spectroscopic tests in the UK can be made based on the reported incidence of brain tumour in the UK and the proportion of cases currently diagnosed through secondary (non-emergency) care. Annual incidence of malignant brain tumours in the UK is approximately 4,700.[35] Approximately one third of these are likely to present through secondary, non-emergency care. Using the positive predictive value for patients currently referred to neuroimaging in this setting of 3% suggests that for each case there will be 33 non-case patients receiving neuroimaging. In total, in the UK, this would imply 53,000 patients per year in secondary care may be suitable for spectroscopic testing.

For peer review only

Appendix 5 - Sensitivity Analysis

One-way sensitivity analysis (OWSA)
OWSA results for a range of test sensitivity are displayed in S1 and S2 as a series of points on a cost-effectiveness (CE) plane with incremental QALYs (intervention-control) on the x-axis and incremental costs (intervention - control) on the y-axis. Corresponding OWSA results for a range of test specificities in primary and secondary care are shown in Figure 3 and 4 in the main article text respectively. These are displayed with the ICER on the y-axis and the test specificity on the x-axis. Note that the estimated QALYs do not change with specificity in the model therefore changes in the ICER are due solely to changes in incremental costs.**Error! Reference source not found.** All OWSA analyses show results assuming the spectroscopy based test costs of £50 and £100.

Figure S1 – Results on CE plane over a range of test sensitivities (0·5-0·99) – Primary care

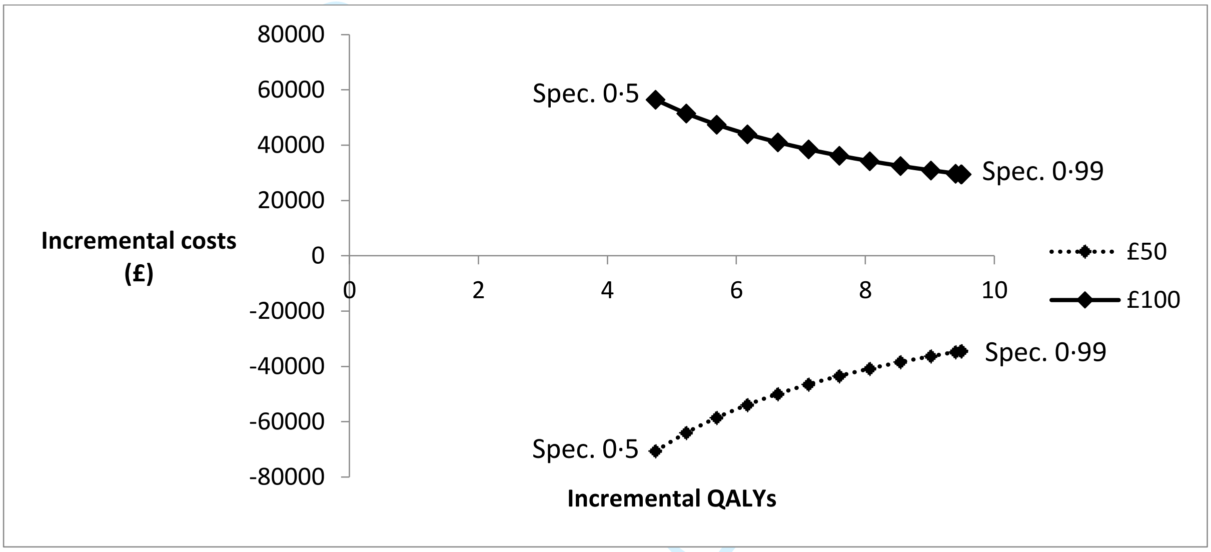

Figure S2 - Results on CE plane over a range of test sensitivities (0·5-0·99) – Secondary care

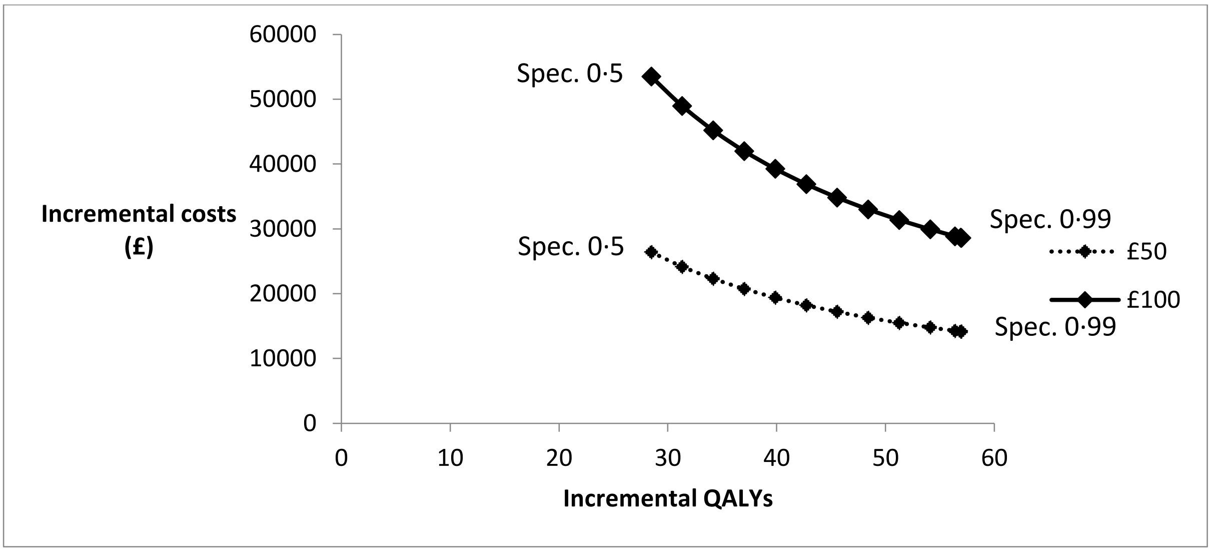

Scenario analysis

Table S3 – Scenario analysis: Mean survival estimates

	Scenario 1			Scenario 2		
Serum spectroscopy test cost (£)	ΔQALY	ΔCost	ICER	ΔQALY	ΔCost	ICER
50	12·83	-212808	-16582 (dominates)	77	540273	7016
100	12·83	287192	22377	77	1040273	13509

Table S4 - Additional costs scenario analysis

	Scenario 1			Scenario 2		
Serum spectroscopy test cost (£)	ΔQALY	ΔCost	ICER	ΔQALY	ΔCost	ICER
50	8·81	-379962	-43128 (dominates)	52·86	566247	10712
100	8·81	120038	13625	52·86	1066247	20171

Table S5 - Alternative prevalence estimates (1% prevalence) in primary care setting (Scenario 1)

	Scenario 1		
Serum spectroscopy test cost (£)	ΔQALY	ΔCost	ICER
50	17·62	-413220	-23452 (dominates)
100	17·62	86780	4925

Table S6– Alternative proportion of test negative cases referred to imaging (Scenario 1 only)

		Scenario 1		
Serum spectroscopy test cost (£)	% of test negative cases referred	ΔQALY	ΔCost	ICER
50	50 (base)	8·81	-422,116	-47,913
	75	17·62	-131,050	-7,438
	90	17·62	38,252	2,171
	100	17·62	151,120	8,577
100	50 (base)	8·81	77,884	8,840
	75	17·62	368,950	20,939
	90	17·62	538,252	30,548
	100	17·62	651,120	36,953

For peer review only

References

1 Burnet, N., Jefferies, S., Benson, R., Hunt, D. & Treasure, F. Years of life lost (YLL) from cancer is an important measure of population burden—and should be considered when allocating research funds. *Brit J Cancer* 2005; **92**: 241–5.

2 Hands, J. R., Clemens, G., Stables, R. *et al.* Brain tumour differentiation: rapid stratified serum diagnostics via attenuated total reflection Fourier-transform infrared spectroscopy. *J Neurooncol* 2016; **127**: 463–72.

3 Smith, B. R., Ashton, K. M., Brodbelt, A. *et al.* Combining random forest and 2D correlation analysis to identify serum spectral signatures for neuro-oncology. *Analyst* 2016; **141**: 3668–78.

4 Finding a better way? Improving the quality of life for people affected by brain tumours, Report Published by The Brain Tumour Charity, 2014.

5 Aggarwal, A., Herz, N., Campbell, P. *et al.* Diagnostic delay and survival in high-grade gliomas - evidence of the 'waiting time paradox'? *Br J Neurosurg* 2015; **29**: 520–3.

6 Zhou, Y., Abel, G. A., Hamilton, W. *et al.* Diagnosis of cancer as an emergency: a critical review of current evidence. *Nat Rev Clin Oncol* 2017; **14**: 45–56.

7 McPhail, S., Elliss-Brookes, L., Shelton, J. *et al.* Emergency presentation of cancer and short-term mortality. *Brit J Cancer* 2013; **109**: 2027–34.

8 Blanks, R., Moss, S., McGahan, C., Quinn, M. & Babb, P. Effect of NHS breast screening programme on mortality from breast cancer in England and Wales, 1990-8: comparison of observed with predicted mortality. *BMJ* 2000; **321**: 665–9.

9 UK Colorectal Cancer Screening Pilot Group. Results of the first round of a demonstration pilot of screening for colorectal cancer in the United Kingdom. *BMJ* 2004; **329**: 133.

10 Quinn, M., Babb, P., Jones, J. & Allen, E. Papers Effect of screening on incidence of and mortality from cancer of cervix in England: evaluation based on routinely collected statistics. *BMJ* 1999; **318**: 904.

11 Frankel, S., Smith, G. D., Donovan, J. & Neal, D. Screening for prostate cancer. *The Lancet* 2003; **361**: 1122–8.

12 Gadalla, S., Pfeiffer, R., Kristinsson, S. *et al.* Brain tumors in patients with myotonic dystrophy: a population-based study. *Eur J Neurol* 2016; **23**: 542–47.

13 Callaghan, B. C., Kerber, K. A., Pace, R. J., Skolarus, L. E. & Burke, J. F. Headaches and neuroimaging: high utilization and costs despite guidelines. *JAMA Intern Med* 2014; **174**: 819–21.

14 National Institute for Health and Care Excellence, 2015. Suspected cancer: recognition and referral. NICE Guideline (MG12).

15 Livermore, L. J., Ma, R., Bojanic, S. & Pereira, E. A. Yield and complications of frame-based and frameless stereotactic brain biopsy–the value of intra-operative histological analysis. *Brit J Neurosurg* 2014; **28**: 637–44.

16 Weber, M. A., Zoubaa, S., Schlieter, M. *et al.* Diagnostic performance of spectroscopic and perfusion MRI for distinction of brain tumors. *Cancer Imaging* 2006; **6**: S32–S41.

17 Baker, M. J., Trevisan, J., Bassan, P. *et al.* Using Fourier transform IR spectroscopy to analyze biological materials. *Nature Protoc* 2014; **9**: 1771–91.

18 Lovergne, L., Bouzy, P., Untereiner, V. *et al.* Biofluid infrared spectro-diagnostics: pre-analytical considerations for clinical applications. *Faraday Discuss* 2016; **187**: 521–37.

For peer review only

19 Baker, M. J., Hussain, S. R., Lovergne, L. *et al.* Developing and understanding biofluid vibrational spectroscopy: a critical review. *Chem Soc Rev* 2016; **45**: 1803–18.
20 Hochberg, F. H., Atai, N. A., Gonda, D. *et al.* Glioma diagnostics and biomarkers: an ongoing challenge in the field of medicine and science. *Expert Rev Mol Diagn* 2014; **14**: 439–52.
21 Hands, J. R., Abel, P., Ashton, K. *et al.* Investigating the rapid diagnosis of gliomas from serum samples using infrared spectroscopy and cytokine and angiogenesis factors. *Anal Bioanal Chem* 2013; **405**: 7347–55.
22 Wang, H. Z., Simonson, T. M., Greco, W. R. & Yuh, W. T. C. Brain MR Imaging in the Evaluation of Chronic Headache in Patients without Other Neurologic Symptoms. *Acad Radiol* 2001; **8**: 405–8.
23 Sempere, A. P., Porta-Etessam, J., Medrano, V. *et al.* Neuroimaging in the evaluation of patients with non-acute headache. *Cephalalgia* 2005; **25**: 30–5.
24 Clarke, C. E., Edwards, J., Nicholl, D. J. & Sivaguru, A. Imaging results in a consecutive series of 530 new patients in the Birmingham Headache Service. *J Neurol* 2010; **257**: 1274–9.
25 Kernick, D., Stapley, S., Goadsby, P. J. & Hamilton, W. What happens to new-onset headache presented to primary care? A case-cohort study using electronic primary care records. *Cephalalgia* 2008; **28**: 1188–95.
26 Hamilton, W. & Kernick, D. Clinical features of primary brain tumours: a case–control study using electronic primary care records. *Brit J Gen Pract* 2007; **57**: 695–9.
27 Do, V., Gebski, V. & Barton, M. The effect of wating for radiotherapy for grade II/IV gliomas. *Radiother Oncol* 2000; **57**: 131–6.
28 Burnet, N., Jefferies, S., Benson, R., Hunt, D. & Treasure, F. Years of life lost (YLL) from cancer is an important measure of population burden—and should be considered when allocating research funds. *Brit J Cancer* 2005; **92**: 241–45.
29 Garside, R., Pitt, M., Anderson, R. *et al.* The effectiveness and cost-effectiveness of carmustine implants and temozolomide for the treatment of newly diagnosed high-grade glioma: a systematic review and economic evaluation. *Health Technology Assessment* 2007; **11**.
30 National Institute for Health and Care Excellence, 2013. Guide to the methods of technology appraisal. NICE Guideline (PMG9).
31 Simpson, G. C., Forbes, K., Teasdale, E., Tyagi, A. & Santosh, C. Impact of GP direct-access computerised tomography for the investigation of chronic daily headache. *Br J Gen Pract* 2010; **60**: 897–901.
32 Taylor, T., Evangelou, N., Porter, H., Hamilton, W. & Kernick, D. Headache: two views on the right approach in general practice. *Br J Gen Pract* 2014; **64**: 475–6.
33 Taylor, T. R., Evangelou, N., Porter, H. & Lenthall, R. Primary care direct access MRI for the investigation of chronic headache. *Clin Radiol* 2012; **67**: 24–7.
34 White, P., Halliday-Pegg, J. & Collie, D. Open access neuroimaging for general practitioners - diagnostic yield and influence on patient management. *Brit J Gen Pract* 2002; **52**: 33–35.
35 UK Statistics Authority. Cancer Registration Statistics, England, 2014. Office of National Statistics

For peer review only

CHEERS Checklist

Items to include when reporting economic evaluations of health interventions

The **ISPOR CHEERS Task Force Report**, *Consolidated Health Economic Evaluation Reporting Standards (CHEERS)—Explanation and Elaboration: A Report of the ISPOR Health Economic Evaluations Publication Guidelines Good Reporting Practices Task Force*, provides examples and further discussion of the 24-item CHEERS Checklist and the CHEERS Statement. It may be accessed via the *Value in Health* or via the ISPOR Health Economic Evaluation Publication Guidelines – CHEERS: Good Reporting Practices webpage: http://www.ispor.org/TaskForces/EconomicPubGuidelines.asp

Section/item	Item No	Recommendation	Reported on page No/ line No
**Title and abstract**			
Title	1	Identify the study as an economic evaluation or use more specific terms such as "cost-effectiveness analysis", and describe the interventions compared.	1 / 1
Abstract	2	Provide a structured summary of objectives, perspective, setting, methods (including study design and inputs), results (including base case and uncertainty analyses), and conclusions.	3 / 1-29
**Introduction**			
Background and objectives	3	Provide an explicit statement of the broader context for the study.	5/2-12
		Present the study question and its relevance for health policy or practice decisions.	8/17-29
**Methods**			
Target population and subgroups	4	Describe characteristics of the base case population and subgroups analysed, including why they were chosen.	14/ 11-37
Setting and location	5	State relevant aspects of the system(s) in which the decision(s) need(s) to be made.	14/11-37
Study perspective	6	Describe the perspective of the study and relate this to the costs being evaluated.	26/3-32
Comparators	7	Describe the interventions or strategies being compared and state why they were chosen.	11-12/33-2
Time horizon	8	State the time horizon(s) over which costs and consequences are being evaluated and say why appropriate.	12/14-22
Discount rate	9	Report the choice of discount rate(s) used for costs and outcomes and say why appropriate.	12/2-3
Choice of health outcomes	10	Describe what outcomes were used as the measure(s) of benefit in the evaluation and their relevance for the type of analysis performed.	11/26-32
Measurement of effectiveness	11a	*Single study-based estimates:* Describe fully the design features of the single effectiveness study and why the single study was a sufficient source of clinical effectiveness data.	11/9-22

For peer review only

	11b	*Synthesis-based estimates:* Describe fully the methods used for identification of included studies and synthesis of clinical effectiveness data.	
Measurement and valuation of preference based outcomes	12	If applicable, describe the population and methods used to elicit preferences for outcomes.	9/1
Estimating resources and costs	13a	*Single study-based economic evaluation:* Describe approaches used to estimate resource use associated with the alternative interventions. Describe primary or secondary research methods for valuing each resource item in terms of its unit cost. Describe any adjustments made to approximate to opportunity costs.	11/33-40
	13b	*Model-based economic evaluation:* Describe approaches and data sources used to estimate resource use associated with model health states. Describe primary or secondary research methods for valuing each resource item in terms of its unit cost. Describe any adjustments made to approximate to opportunity costs.	12-13/4-4<br>24/1-17
Currency, price date, and conversion	14	Report the dates of the estimated resource quantities and unit costs. Describe methods for adjusting estimated unit costs to the year of reported costs if necessary. Describe methods for converting costs into a common currency base and the exchange rate.	11/38-40
Choice of model	15	Describe and give reasons for the specific type of decision-analytical model used. Providing a figure to show model structure is strongly recommended.	9/1
Assumptions	16	Describe all structural or other assumptions underpinning the decision-analytical model.	23-24/1-6
Analytical methods	17	Describe all analytical methods supporting the evaluation. This could include methods for dealing with skewed, missing, or censored data; extrapolation methods; methods for pooling data; approaches to validate or make adjustments (such as half cycle corrections) to a model; and methods for handling population heterogeneity and uncertainty.	12-13/4-4
**Results**			15/3-20
Study parameters	18	Report the values, ranges, references, and, if used, probability distributions for all parameters. Report reasons or sources for distributions used to represent uncertainty where appropriate. Providing a table to show the input values is strongly recommended.	
Incremental costs and outcomes	19	For each intervention, report mean values for the main categories of estimated costs and outcomes of interest, as well as mean differences between the comparator groups. If applicable, report incremental cost-effectiveness ratios.	15/3-20
Characterising uncertainty	20a	*Single study-based economic evaluation:* Describe the effects of sampling uncertainty for the estimated incremental cost and incremental effectiveness parameters, together with the impact	

For peer review only

		of methodological assumptions (such as discount rate, study perspective).	
	20b	*Model-based economic evaluation:* Describe the effects on the results of uncertainty for all input parameters, and uncertainty related to the structure of the model and assumptions.	17-18/2-2
Characterising heterogeneity	21	If applicable, report differences in costs, outcomes, or cost-effectiveness that can be explained by variations between subgroups of patients with different baseline characteristics or other observed variability in effects that are not reducible by more information.	
**Discussion**			
Study findings, limitations, generalisability, and current knowledge	22	Summarise key study findings and describe how they support the conclusions reached. Discuss limitations and the generalisability of the findings and how the findings fit with current knowledge.	19-20/4-20
**Other**			
Source of funding	23	Describe how the study was funded and the role of the funder in the identification, design, conduct, and reporting of the analysis. Describe other non-monetary sources of support.	2/28-33
Conflicts of interest	24	Describe any potential for conflict of interest of study contributors in accordance with journal policy. In the absence of a journal policy, we recommend authors comply with International Committee of Medical Journal Editors recommendations.	2/23-26

For consistency, the CHEERS Statement checklist format is based on the format of the CONSORT statement checklist

The **ISPOR CHEERS Task Force Report** provides examples and further discussion of the 24-item CHEERS Checklist and the CHEERS Statement. It may be accessed via the *Value in Health* link or via the ISPOR Health Economic Evaluation Publication Guidelines – CHEERS: Good Reporting Practices webpage: http://www.ispor.org/TaskForces/EconomicPubGuidelines.asp

The citation for the CHEERS Task Force Report is:
Husereau D, Drummond M, Petrou S, et al. Consolidated health economic evaluation reporting standards (CHEERS)—Explanation and elaboration: A report of the ISPOR health economic evaluations publication guidelines good reporting practices task force. Value Health 2013;16:231-50.

For peer review only
